# GhNAC83 inhibits corm dormancy release by regulating ABA signaling and cytokinin biosynthesis in *Gladiolus hybridus*

**DOI:** 10.1093/jxb/ery428

**Published:** 2018-12-05

**Authors:** Jian Wu, Yujie Jin, Chen Liu, Eliana Vonapartis, Jiahui Liang, Wenjing Wu, Sonia Gazzarrini, Junna He, Mingfang Yi

**Affiliations:** 1 Beijing Key Laboratory of Development and Quality Control of Ornamental Crops, Department of Ornamental Horticulture and Landscape Architecture, China Agricultural University, Beijing, China; 2 Department of Cell and Systems Biology, University of Toronto, ON, Canada; 3 Department of Biological Sciences, University of Toronto Scarborough, ON, Canada

**Keywords:** ABA, corm, cytokinins, dormancy, gladiolus, NAC

## Abstract

Corm dormancy is an important trait for breeding in many bulb flowers, including the most cultivated *Gladiolus hybridus*. *Gladiolus* corms are modified underground stems that function as storage organs and remain dormant to survive adverse environmental conditions. Unlike seed dormancy, not much is known about corm dormancy. Here, we characterize the mechanism of corm dormancy release (CDR) in *Gladiolus*. We identified an important ABA (abscisic acid) signaling regulator, GhPP2C1 (protein phosphatase 2C1), by transcriptome analysis of CDR. *GhPP2C1* expression increased during CDR, and silencing of *GhPP2C1* expression in dormant cormels delayed CDR. Furthermore, we show that *GhPP2C1* expression is directly regulated by GhNAC83, which was identified by yeast one-hybrid library screening. *In planta* assays show that GhNAC83 is a negative regulator of *GhPP2C1*, and silencing of *GhNAC83* promoted CDR. As expected, silencing of *GhNAC83* decreased the ABA level, but also dramatically increased cytokinin (CK; zeatin) content in cormels. Binding assays demonstrate that GhNAC83 associates with the *GhIPT* (*ISOPENTENYLTRANSFERASE*) promoter and negatively regulates zeatin biosynthesis. Taken together, our results reveal that GhNAC83 promotes ABA signaling and synthesis, and inhibits CK biosynthesis pathways, thereby inhibiting CDR. These findings demonstrate that GhNAC83 regulates the ABA and CK pathways, and therefore controls corm dormancy.

## Teaser

GhNAC83 inhibits corm dormancy release in *Gladiolus hybridus* by promoting ABA signaling and inhibiting CK biosynthesis.

## Introduction

In most countries, summer-flowering *Gladiolus* cultivars are widely planted and are among the most important cut flowers. Summer-flowering *Gladiolus* shows great diversity in plant height, flower color, number of florets, and flower size. During the *Gladiolus* growing season, a new corm is produced over the mother corm. Afterwards, cormels are formed at the tips of branched stolons that develop from buds located at the base of the new corm (Le Nard, 1993). In autumn, the corms and cormels are lifted out of the ground and placed in a cold storage house to accelerate corm dormancy release (CDR; ~2–3 months) before the next planting (Wu *et al.*, 2015). Understanding the mechanism of CDR to shorten the growth season is of great interest to the flower industry.

In *Gladiolus*, ABA (abscisic acid) is the key inhibitor of CDR, and *GhABI5* (*ABA INSENSITIVE 5*) has been shown to delay CDR. GA (gibberellic acid) plays a minor role in this process (Ginzburg, 1973; Wu *et al.*, 2015). Moreover, 6-BA [6-benzylaminopurine; an exogenous aromatic cytokinin (CK)] increases dark CO_2_ fixation rates in dormant *Gladiolus* cormels, indicating that 6-BA has a positive role in CDR (Ginzburg, 1981). However, the molecular mechanisms of ABA’s and CK’s antagonistic regulation of CDR are unknown.

In Arabidopsis, ABA controls seed dormancy by inhibiting the activities of clade A PP2Cs, a group of protein phosphatases (PPs) including ABI1/2 (ABA INSENSITIVE 1/2) and HAB1/2 (HYPERSENSITIVE TO ABA 1/2), which act as co-receptors with PYR1/PYL/RCAR (PYRABACTIN RESISTANT/PR1-LIKE/REGULATORY COMPONENT OF ABA RECEPTOR) in ABA signaling (Ma *et al.*, 2009; X. Wang *et al.*, 2018). These protein phosphatases play important roles in seed germination and abiotic stress responses (Gosti *et al.*, 1999; Kong *et al.*, 2015). When ABA levels increase, clade A PP2Cs lose the ability to inhibit the activity of SnRK2s (class II SNF1-related protein kinase 2) activating downstream ABA responses (Hubbard *et al.*, 2010). In strawberries, silencing of *FaABI1* promotes fruit ripening, indicating that ABI1 has an inhibitory role in fruit ripening (Jia *et al.*, 2013). In recent years, upstream regulators of PP2Cs have been identified and shown to function in salt stress (MYB20), leaf senescence (AtNAP; NON-INTRINSIC ABC PROTEIN), drought response (AtHB7/12; HOMEOBOX 7/12), and water stress (ORA47; octadecanoid-responsive AP2/ERF-domain transcription factor 47) ( Valdes *et al*., 2012; Zhang and Gan, 2012; Cui *et al.*, 2013; Chen *et al.*, 2016).

CKs are involved in delaying leaf senescence, promoting differentiation of the shoot and root meristems, seed germination, and stress responses (Werner *et al.*, 2003; Dong *et al.*, 2008; Choi *et al.*, 2010; Wang *et al.*, 2011; Verslues, 2016). The relationship between ABA and CKs varies depending on the species and biological process (Bozhkov *et al.*, 1992; Zubo *et al.*, 2008; Wang *et al.*, 2011). CKs have been shown to antagonize ABA’s role in seed dormancy by inhibiting ABI5 expression (Wang *et al.*, 2011). Adenosine phosphate-isopentenyltransferase (IPT) and CYP735As (CYTOCHROME P450, FAMILY 735, SUBFAMILY As) catalyze important steps of CK biosynthesis to produce *trans*-zeatin (Takei *et al.*, 2004; Sakakibara, 2006; Tarkowska and Strnad, 2018). Endogenous CKs activate the receptor gene *Cytokinin Response 1* (*CRE1*) to initiate phosphorelay signaling (Inoue *et al.*, 2001). In Arabidopsis, the core signaling pathway consists of His kinases (AHKs), His phosphotransfer proteins (AHPs), and response regulators (ARRs). ARR4/5/6 interact with, and negatively regulate, *ABI5* expression during seed germination (Wang *et al.*, 2011). The antagonistic roles of ABA and CKs/GA have also been shown in potato tuber sprouting and are possibly linked to altering SnRK1 (Sucrose non fermenting Related Kinase1)/T6P (TREHALOSE-6-PHOSPHATE) signaling (Subbaraj *et al.*, 2010; Sonnewald and Sonnewald, 2014). However, the molecular mechanisms of CK–ABA interaction in dormancy release are still unclear.

NAC transcription factors (TFs) form one of the largest TF families in plants, and are classified into 24 groups (Jensen *et al.*, 2010). The NACs recognize the consensus *cis*-acting elements CGT(G/A) and CACG (Cao *et al.*, 2017). In Arabidopsis, NACs play roles in plant development (Ko *et al.*, 2007), senescence (Yang *et al.*, 2011), drought (Park *et al.*, 2009; You *et al.*, 2014), cold tolerance (Shan *et al.*, 2014), and biotic stress (Seo *et al.*, 2010). Some NACs (ATAF1) mediate signaling in response to both pathogen and abiotic stresses (Wu *et al.*, 2009). NACs have been implicated in the regulation of an ABA biosynthesis gene (*NCED*; *9-CIS-EPOXYCAROTENOID DIOXYGENASE*) and an ABA response gene (*RD29*; *RESPONSIVE TO DESICCATION 29*), further modulating drought stress (Wu *et al.*, 2009; Jensen *et al.*, 2013; Xu *et al.*, 2013). In addition, a membrane-bound NAC (NTM1; NAC WITH TRANSMEMBRANE MOTIF1) has been reported to mediate CK signaling, specifically during cell division (Kim *et al.*, 2006).

Currently, not much is known about how hormones control CDR, particularly the mechanisms behind the antagonistic role that ABA and CKs play in this process. In this study, transcriptome sequencing and functional analysis revealed that *GhPP2C1* positively regulates the CDR. GhNAC83 was found to bind the *GhPP2C1* promoter directly by yeast one-hybrid screening, and further evidence suggests that GhNAC83 is a negative regulator of *GhPP2C1*. We also show that GhNAC83 decreases zeatin content by inhibiting the expression of CK biosynthesis genes (*GhCYP735A* and *GhIPT*). Thus, GhNAC83 positively regulates ABA signaling through down-regulation of *GhPP2C1* and inhibits CK biosynthesis through down-regulation of *GhIPT*, ultimately delaying CDR. Our findings uncover GhNAC83’s role in regulating ABA and CK pathways in the control of corm dormancy.

## Materials and methods

### Plant material and growth conditions

*Gladiolus* ‘Rose Supreme’ was planted and harvested as described previously (Wu *et al.*, 2015). Harvested cormels were dried at 25 °C for 6 weeks, and then were kept in a cold storage room at 4 °C with 60–70% relative humidity.

For expression pattern analysis, tissues and organs were collected at the flowering stage with seven leaves. Cormels at different dormant stages were sampled after harvest (desiccation and cold storage) every 2 weeks.

Sprouting tests were used to determine dormancy release patterns under different hormone treatments. Dormant cormels used in 6-benzylaminopurine (6-BA) treatments measured 0.5 cm in diameter. These cormels were sterilized first and then were embedded in 0.6% (w/v) agar plates which contained different concentrations of 6-BA (0, 25, 50, and 100 μM) before being placed in a plant growth chamber at 25 °C with 12 h/12 h light/dark. The sprouting percentage was counted on the 20th day after plating. Sprouting was defined as a bud on the top of the cormel elongated >5 mm (Luo *et al.*, 2012). Thirty cormels per sample were used for each sprouting test. Error bars in the figures represent the SD of three biological replicates. Non-dormant cormels were used for ABA treatments (0, 25, 50, and 100 μM), and the sprouting test was the same as explained above.

### Transcriptome analysis

Samples for RNA sequencing (RNA-seq) were collected at deep dormancy (DD; 19 December 2012), weak dormancy (WD; 17 January 2013), and ecodormancy (ED; 9 May 2013) stages (Wu *et al.*, 2015). Three biological samples were collected for each stage, frozen immediately in liquid nitrogen, and stored in a freezer at –80 °C until RNA extraction. The sprouting percentage was counted on the 20th day after planting on soil. Sprouting was defined as a bud on the top of the cormel elongated >5 mm (Luo *et al.*, 2012). Thirty cormels per stage were used for each sprouting test. Error bars in the figures represent the SD of three biological repeats.

Total RNA from *Gladiolus* cormels was extracted using the Tiangen RNA extraction reagent kit (Tiangen, Beijing, China). RNA was quantified using a NanoDrop 2000 (Thermo Scientific, DE, USA) and its quality was determined by an Agilent 2100 Bioanalyzer (Agilent Technologies, CA, USA). High-quality RNA (RNA integrity number >9.0) was selected for cDNA library preparation. Strand-specific RNA libraries were constructed as previously described (Zhang *et al.*, 2015).

The RNA-seq libraries were sequenced in a single lane of a Hiseq 2500 platform at the Novogene Company (Beijing, China) and 150 bp paired-end reads were generated (10-fold depth of RNA sequencing). The raw sequence reads were deposited in the NCBI Sequence Read Archive (SRA) database under the accession number PRJNA491310.

Raw data were filtered to remove low-quality reads, and adaptor sequences were trimmed using Trimmonmatic (Bolger *et al.*, 2014). The resulting data were then aligned to the rRNA sequence databases (Quast *et al.*, 2013) and the GenBank virus database using Burrows–Wheeler aligner (BWA) with default parameters (Li and Durbin, 2010). Mapped reads in these two databases were discarded. Only high-quality clean reads were used in the following analysis.

*De novo* transcriptome assembly was performed using the Trinity program (Grabherr *et al.*, 2011). To delete the redundant Trinity-assembled contigs, the contigs were further assembled using iAssembler (Zheng *et al.*, 2011). All assembled unigenes were subjected to the NCBI non-redudant protein (Nr) database, Swiss-prot database, Nucleotide database (Nt), Cluster of Orthologous Groups (COG) database, Gene Ontology (GO), and Kyoto Encyclopedia of Genes and Genomes (KEGG) database with a typical cut-off E-value of 1E^−5^. Based on the annotation, BLAST2GO (Conesa *et al.*, 2005) was assigned to obtain the GO annotation for describing the biological processes, cellular components, and molecular functions. The COG database was used to classify unigene functions (Tatusov *et al.*, 2000). The KEGG pathway of unigenes was annotated by mapping the resulting sequences from BLAST2GO to the contents of the KEGG metabolism pathway database (Kanehisa and Goto, 2000).

### Isolation of full-length *GhPP2C1* and *GhNAC83* cDNAs, and sequence analysis

The full-length *GhPP2C1* sequence was cloned by RACE according to the manufacturer’s instructions (Clontech). The full-length *GhNAC83* sequence was directly isolated from our transcriptome database by PCR (Supplementary Table S1 at *JXB* online).

Multiple amino acid alignments were performed using ClustalX1.8 and BioEdit7.0 (Chenna *et al.*, 2003; Hall, 2005), and phylogenetic trees were constructed by the maximum likelihood method using the MEGA5.0 software (Tamura *et al.*, 2011).

### Quantitative real-time-PCR

Total RNA was extracted using the Tiangen RNA extraction reagent kit. A 1 μg aliquot of DNase-treated RNA was used to synthesize cDNA by M-MLV (Takara). About 400 ng of cDNA was used as the template for real-time PCRs (RT-PCRs) and was run by the Step One Plus real-time PCR system (Applied Biosystems) using the SYBR Premix Ex Taq kit (Takara). *GhActin* (accession no. JF831193) was used as the internal control. The PCR procedure was performed according the manufacturer’s instructions. Primers used are listed in Supplementary Table S1.

### Virus-induced gene silencing

Silencing of *GhPP2C1* or *GhNAC83* in dormant cormels was conducted as previously described (Zhong *et al.*, 2014; Wu *et al.*, 2015), with some modifications. Freshly grown *Agrobacterium tumefaciens* GV3101 cells harboring pTRV1, pTRV2, pTRV2-GhPP2C1, or pTRV2-GhNAC83 vectors were collected and suspended in infiltration buffer (10 mM MgCl_2_, 200 mM acetosyringone, 10 mM MES, pH 5.6) to a final OD_600_ of 2.0. A mixture containing equal volumes of pTRV1 and pTRV2-GhPP2C1 or pTRV2-GhNAC83 cultures were used for the *GhPP2C1-TRV2* and *GhNAC83-TRV2* experiments, respectively. A mixture containing an equal volume of pTRV1 and pTRV2 cultures was used as the control (TRV2). The mixtures were stored at 25 °C for 3 h in darkness. Vacuum infiltration of dormant cormels and later growth stages was performed as previously described (Wu *et al.*, 2015). Three independent experiments were conducted with 24 silenced cormels in each experiment. The silenced plantlets were imaged and analyzed after 10 d on soil.

### Promoter analysis, cloning, and transient expression assay in *Nicotiana benthamiana*

The upstream regulatory sequence (URS) of *GhPP2C1* was cloned using high-efficiency thermal asymmetric interlaced PCR (Hi-TAIL) (Liu and Chen, 2007). The *cis*-regulatory elements were annotated using PlantCARE (Lescot *et al.*, 2002), and potential TF-binding sites were analyzed using PlantPan 2.0 (Chow *et al.*, 2016).

The URS and truncated URSs were inserted into the pCAMBIA1391 binary vector. *GhPP2C1*:*GUS* was then introduced into GV3101 for *N. benthamiana* infiltration. *Agrobacterium tumefaciens* cells harboring the truncated promoter fragments were suspended in infiltration buffer (10 mM MgCl_2_, 200 mM acetosyringone, 10 mM MES, pH 5.6) to an OD_600_ of 0.8, then each suspension was infiltrated into different regions of the same *N. benthamiana* leaf. After 3 d, the infiltrated leaves were immersed in GUS (β-glucuronoidase) staining solution overnight and were decolorized using 70% ethanol (Chen *et al.*, 2013). Three independent experiments were conducted with 12 leaves from six plants in each experiment.

### Yeast one-hybrid screening

Yeast one-hybrid library screening was performed as previously described (Deplancke *et al.*, 2006), with some modifications. The *GhPP2C1* truncated promoter (base pairs –833 to –615) was recombined into the pDEST-HISi-2 vector by Gateway cloning. Then the linearized vector was transformed into yeast strain YM4271(a) using the PEG/LiAc method. Transformed yeast colonies were tested for background expression of the *HIS3* reporter, and the appropriate 3-aminotriazole (3-AT) concentration was selected. An *Arabidopsis thaliana* TF library (Mitsuda *et al.*, 2010) was transformed into yeast strain EGY48(α) by electroporation. Mutagenesis of the *GhPP2C1* promoter was generated by PCR-driven overlap extension (Heckman and Pease, 2007). The same method of mutagenesis was used to generate the mutant *GhIPT* promoter used below. Primers are listed in Supplementary Table S1.

To test the interaction between GhNAC83 and the *GhIPT* promoter truncations, the *GhIPT* promoters (T1, T2, T3, and T2^mut^) and GhNAC83 were recombined into pDEST-HISi-2 and pDEST-GAD424, respectively, by Gateway technology. The recombined vectors were then transformed into yeast strain YM4271(a) (for pDEST-HISi-2) and EGY48(α) (for pDEST-GAD424). Transformed YM4271(a) containing the various truncated *GhIPT* promoter regions were first tested for the background *HIS3* expression using increasing 3-AT concentrations (0, 5, 10, 20, and 40 mM). A single transformed YM4271(a) colony requiring the lowest 3-AT concentration (10 mM) from each transformed yeast (T1, T2, T3, and T2^mut^) was used for mating with EGY48(α) containing GhNAC83. Following mating on YPD plates for 16 h, the yeast cells were washed off with water and spread on yeast plates (SD-Ura-His-Leu). The plates were cultured at 28 °C for 3 d to select for diploids. Yeast cultures (OD_600_ diluted to 0.08) were spotted on selection plates (SD-Ura-His-Leu+10 mM 3-AT) and cultured at 28 °C for 3 d. The interaction was judged by the growth of yeast on selection media.

### GUS/LUC assay in *N. benthamiana*

The transient GUS/luciferase (LUC) assay was performed as previously described (Zhao *et al.*, 2016). The constructs (*35S:GhNAC83*/pSuper1300, pSuper1300, *GhPP2C1:GUS*/pCAMBIA1391, and *35S:LUC*) were independently transformed into *A. tumefaciens* strain GV3101. Then, *35S:GhNAC83*, *GhPP2C1:GUS*, and *35S:LU*C (OD_600_=0.8 each; 1000:1000:5 v/v/v) were co-agroinfiltrated into *N. benthamiana*. After 3 d, GUS and LUC activities were measured using methyl umbelliferyl glucuronide (Sigma-Aldrich; 881005-91-0) and the Bio-Glo™ Luciferase Assay System kit (Promega; G7940), respectively. The LUC activity (35S:LUC) was used as an internal control and pSuper1300 was used as a negative control. The GUS/LUC ratio was used to reflect the promoter activity. Three biological replicates were conducted in this assay (*n*=5 leaves).

### Subcellular localization assay

The *GhNAC83* ORF was cloned into pCAMBIA1300-GFP (green fluorescent protein). Both the fusion construct (*GhNAC83-GFP*) and the control (*GFP*) were transformed into *A. tumefaciens* GV3101 and used to agroinfiltrate *N. benthamiana* leaves. GFP fluorescence was observed using confocal microscopy (Nikon Inc., Melville, NY, USA) at 3 d post-infiltration.

### Transactivation domain analysis in yeast

For the transactivation assay in *Saccharomyces cerevisiae* strain AH109, different truncations of the *GhNAC83* coding region were PCR amplified and inserted into the pGBKT7 vector (Clontech, Mountain View, CA, USA) with *Nde*I and *Xma*I sites. The different portions of GhNAC83 fused with the GAL4 DNA-binding domain are as follows: full length (FL; amino acids 1–219), C-terminal part (CP; amino acids 111–219), N-terminal part (NP; amino acids 1–110), and the C-terminus (CT; amino acids 161–219). The primers are listed in Supplementary Table S1. The positive control (pBD-AD; +) and the negative control (pBD; –) were also introduced into AH109 according to the manufacturer’s protocol (Stratagene). Transcriptional activation was tested as described in the yeast protocols handbook (PT3024-1; Clontech).

### Extraction and quantification of phytohormones

The extraction of ABA from *Gladiolus* cormels was performed according to Wu *et al.* (2016). *Gladiolus* cormels (50 mg) were homogenized, and added to an extraction solvent (500 μl; isopropanol/H_2_O/concentrated HCl with a volume ratio of 2:1:2E-3) with 10 ng of internal standard (d_6_-ABA). Samples were inverted at 4 °C (100 rpm, 30 min), and then 1 ml of dichloromethane was added for a second round of inversion. After centrifugation (14000 rpm, 30 min), the lower phase of solvent was transferred to a new tube. The solvent was dried using a DNC-2000 concentrator (Beijing IDES Technology) and was re-dissolved in 100 μl of methanol. The extraction of CKs from cormels was based on the procedure described previously (Antoniadi *et al.*, 2015) with some modifications. Samples (500 mg) were homogenized and extracted using a 5 ml mixture of methanol/water/methanoic acid (15:4:1, v/v/v) containing 20 mg l^–1^ sodium diethyldithiocarbamate. Deuterium-labeled CKs were added to serve as internal standards. Extractions were purified with a Sep-Pak Plus C18 cartridge and Oasis MCX column as described previously (Chen *et al.*, 2010). Then, the column was washed with 1 M methanoic acid (5 ml), and pre-concentrated analytes were eluted by two-step elution using NH_4_OH (5 ml) and 5 ml of 0.35 M NH_4_OH in 60% methanol. The eluate was vacuum evaporated and kept at –80 °C until analysis. Quantitative analysis of ABA and CKs in crude extracts was determined by HPLC-electrospray ionization tandem mass spectrometry (HPLC-ESI-MS/MS) (Pan *et al.*, 2008; Farrow and Emery, 2012). At least three biological replicates were conducted.

### Dual-luciferase reporter assay

The GhNAC coding sequence was cloned into pGreenII 62-SK. A promoter of the *GhPP2C1p*, *GhPP2C1p*^*MUT*^, *GhIPTp*, or *GhIPTp*^*MUT*^ regions was cloned into pGreenII LUC vector (Wei *et al.*, 2017). All constructs were transformed into *A. tumefaciens* strain GV3101 harboring the pSoup helper plasmid. The infiltration and LUC measurements were performed as previously described (Wei *et al.*, 2017).

## Results

### *GhPP2C1* promotes corm dormancy release in *Gladiolus*

**Fig. 1.**
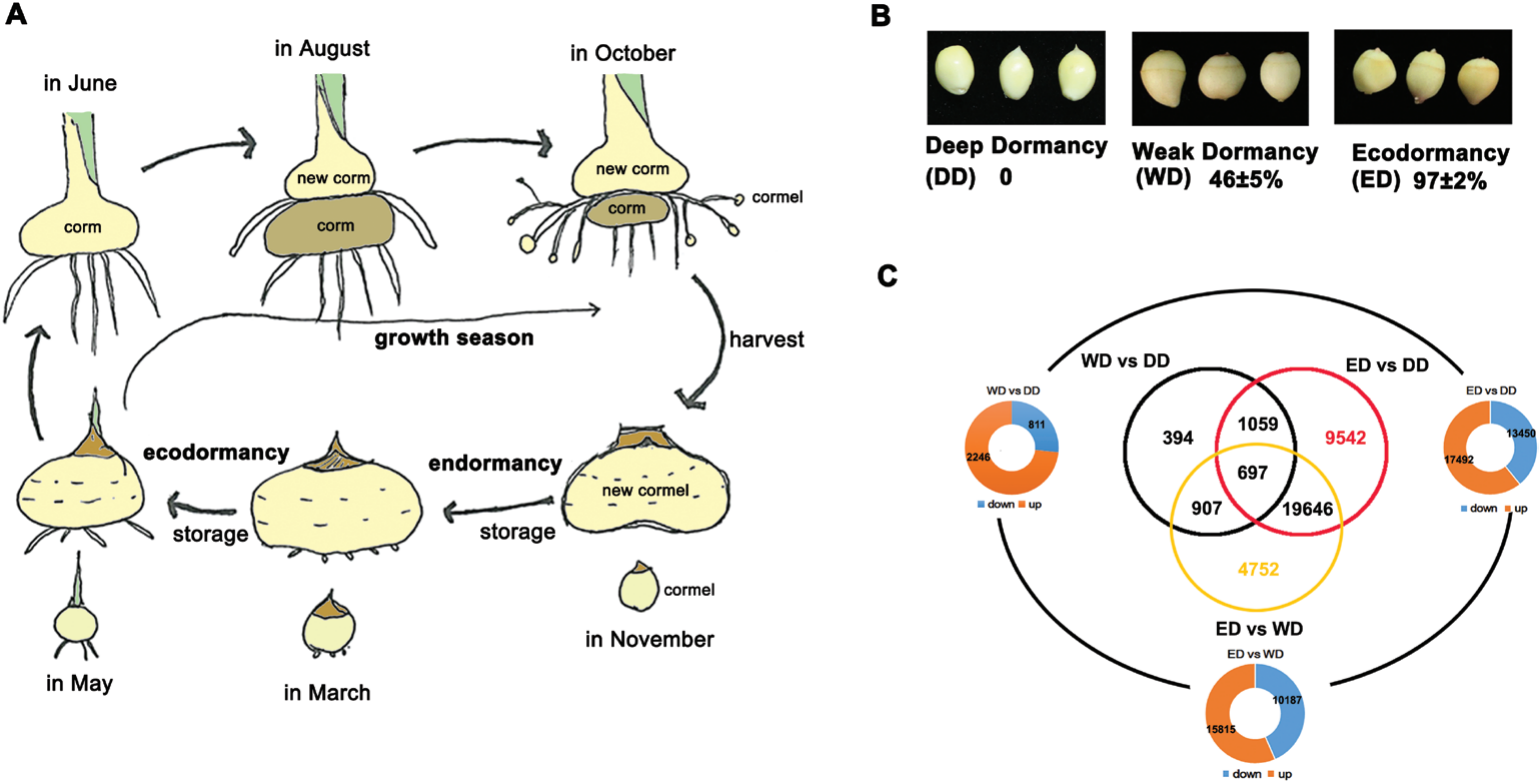
Transcriptome analysis of *Gladiolus* corm dormancy release. (A) Life cycle of *Gladiolus*. Corms >1 cm in diameter are used for cut-flower production. Cormels are planted in the next growing season and develop into corms. (B) Different stages of corm dormancy. DD, deep dormancy; WD, weak dormancy; ED, ecodormancy. Sprouting rates were tested 20 d after planting on soil. Data are shown as means of three replicates ±SD (*n*=30). (C) Differentially expressed genes (DEGs) during *Gladiolus* dormancy release. Genes were considered to be DEGs when there was a cut-off ratio of log2 < –1 or >1 and a *q*-value <0.05. The 697 overlapping DEGs are listed in Supplementary Table S2. (This figure is available in color at *JXB* online.)

**Fig. 2.**
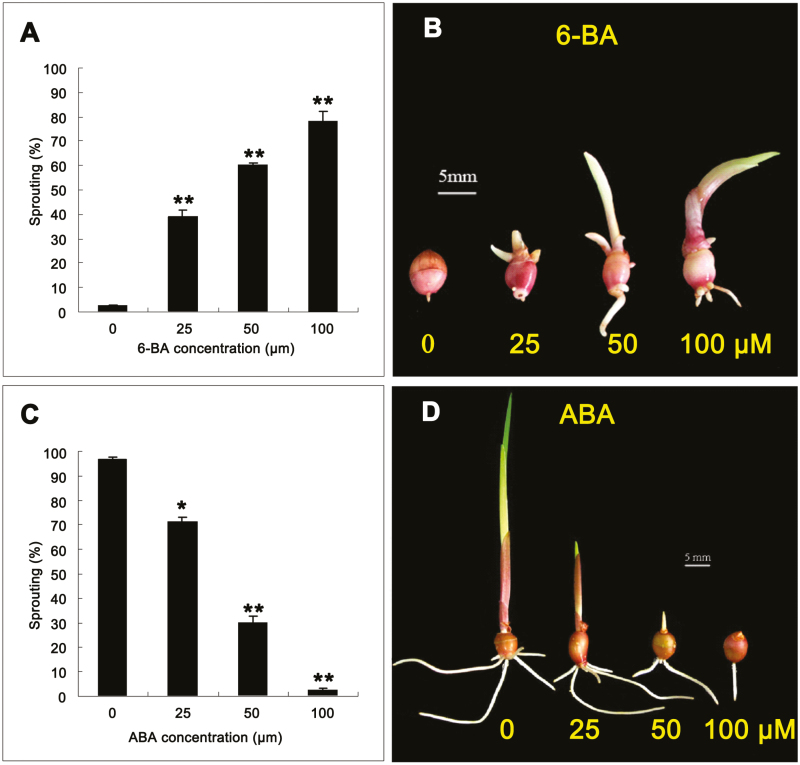
ABA and cytokinins are involved in corm dormancy release. (A) 6-BA promotes sprouting of dormant cormels. (B) The phenotype of dormant cormels exposed to 6-BA for 20 d. (C), ABA inhibits sprouting of non-dormant cormels. (D) The phenotype of non-dormant cormels exposed to ABA for 20 d (*P<0.05 and **P<0.01). Averages of three biological replicates with the SD are shown; *n*=30. (This figure is available in color at *JXB* online.)

To investigate the molecular mechanism of *Gladiolus* CDR, we first tracked sprouting of cormels at different stages (Fig. 1A). We chose deep dormant (DD; unsprouting), weak dormant (WD; half-sprouting), and ecodormant (ED; all-sprouting) cormels for large-scale transcriptome sequencing on the Illumina Hiseq2500 platform using the paired-end protocol (Fig. 1B). To identify genes that are differentially regulated during CDR, differentially expressed genes (DEGs) were screened using a cut-off ratio of log2 < –1 or >1, and a *q*-value of <0.05, and 697 overlapping DEGs were found (Supplementary Table S2). The results in Fig. 1C indicate that the greatest change in gene expression occurred during the ED transition (ED versus WD; 26 002 unigenes) and not in the WD transition (WD versus DD; 3057 unigenes). During the WD transition, GO terms of phytohormone biosynthesis (zeatin and ABA) and plant hormone signal transduction were highly enriched (Supplementary Fig. S1), supporting the opposing roles of these hormones in CDR (Fig. 2).

With respect to phytohormones, ABA-related DEGs, including PP2C family genes, were the most abundant, showing strong up-regulation from DD to WD (Supplementary Table S3). Moreover, three PP2C unigenes (GlaUn030679, GlaUn052869, and GlaUn078852) maintained high transcriptional levels during CDR (Supplementary Table S3).

**Fig. 3.**
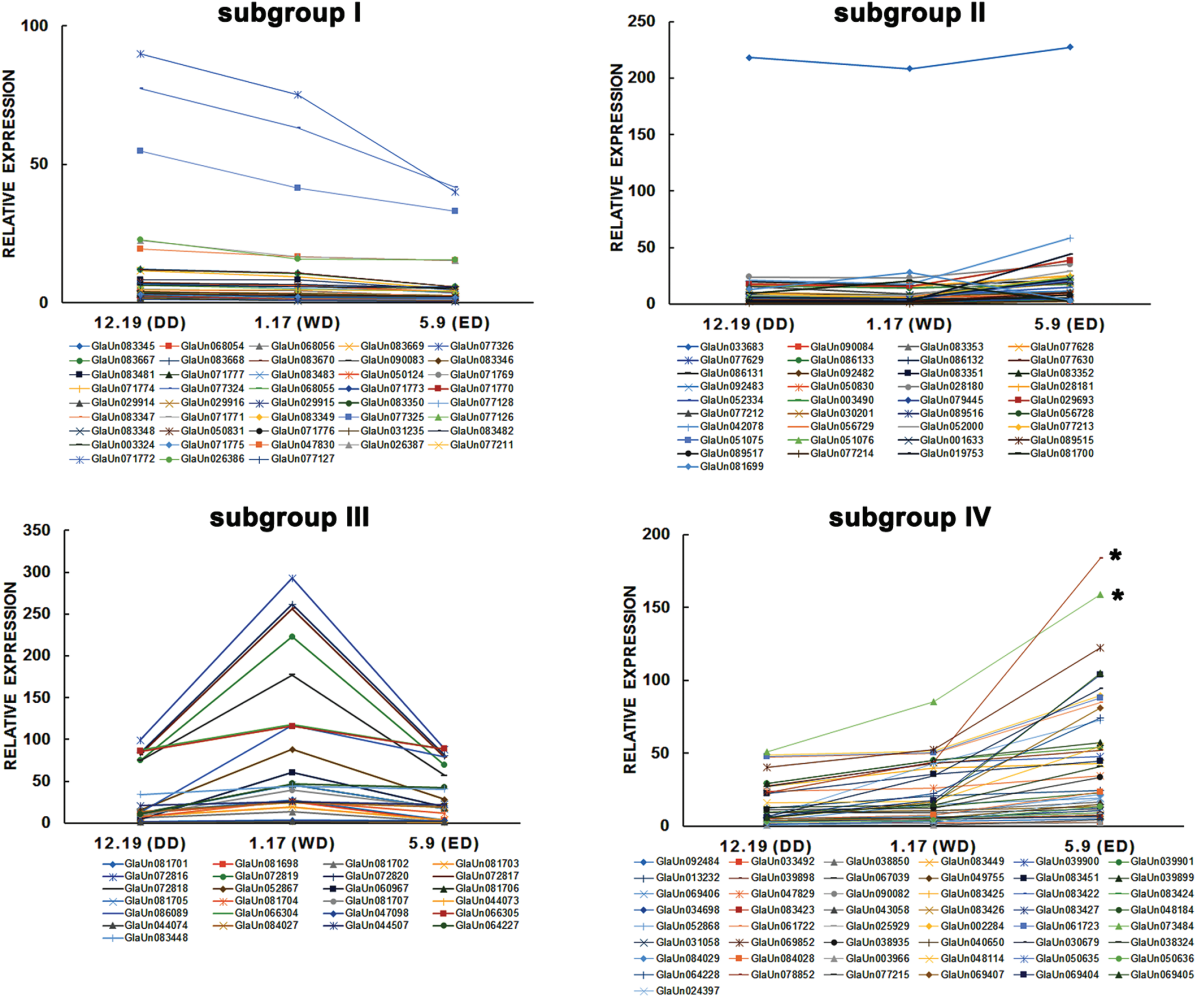
Expression patterns of *GhPP2C* genes in *Gladiolus* CDR. An asterisk (*) represents the selected unigenes (GhPP2C1) from *Gladiolus* CDR transcriptome analysis. Expression of unigenes in the top left panel decreased during CDR (DD→WD→ED). Unigenes in the top right panel decreased in expression from DD to WD, but increased from WD to ED. Expression of unigenes in the bottom left panel increased from DD to WD, but decreased from WD to ED. Expression of unigenes in the bottom right panel increased during CDR (DD→WD→ED). The expression levels are based on a FPKM evaluation. DD, deep dormancy; WD, weak dormancy; ED, ecodormancy. (This figure is available in color at *JXB* online.)

PP2Cs are a part of the core ABA signaling module and are involved in seed dormancy ( Seiler *et al.*, 2011; Nee *et al*., 2017). In order to investigate PP2C’s function in CDR, 154 members were identified in the transcriptome and sorted into four subgroups by their expression pattern: subgroup I (43/154), subgroup II (37/154), subgroup III (25/154), and subgroup IV (49/154) (Fig. 3). When a threshold for change in expression level was set (fold <0.8 or >1.6 and relative expression value >20), only two members (GlaUn078852 and GlaUn073484) met the criteria. The full-length cDNAs of *GlaUn078852* and *GlaUn073484* were amplified from *G. hybridus* cv. ‘Rose Supreme’ cormels by RACE, and were found to be the same gene. Therefore, we selected this gene for further study.

**Fig. 4.**
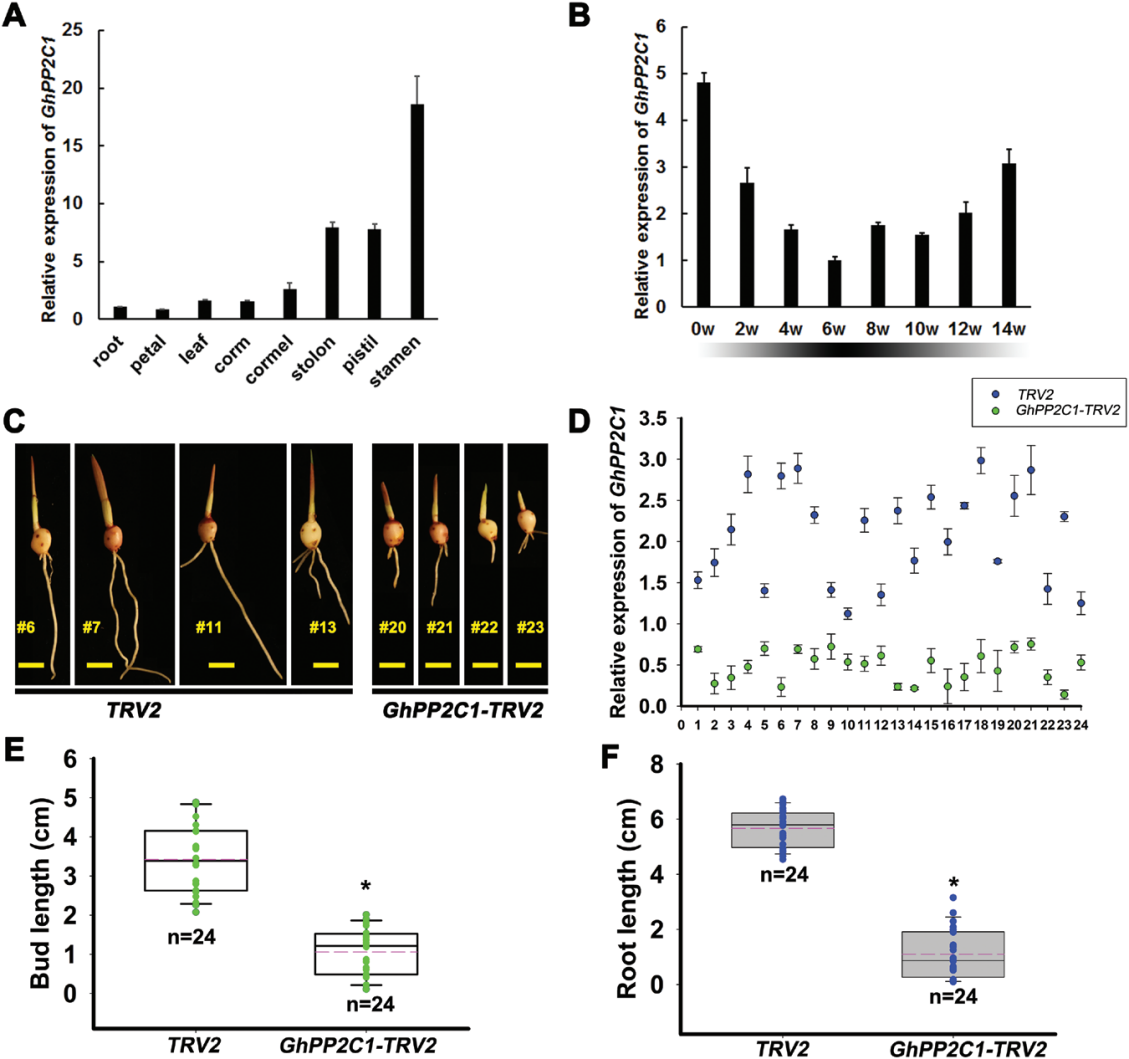
*GhPP2C1* is involved in corm dormancy release. (A) The expression of *GhPP2C1* in different organs at blooming flower stage. (B) The expression pattern of *GhPP2C1* during corm desiccation (weeks 0–6) and cold storage (weeks 6–14). Data in (A) and (B) are displayed as averages of three biological repeats with the SD. (C) Phenotype resulting from *GhPP2C1* silencing 10 d after planting on soil. The scale bar represents 1 cm. (D) The expression of *GhPP2C1* in 24 independent *GhPP2C1-TRV2* lines. Data are shown as averages of three technical replicates with the SD. Bud length (E) and root length (F) in *GhPP2C1-TRV2* and *TRV2* lines; *n*=24 independent lines (*P<0.05; **P<0.01). (This figure is available in color at *JXB* online.)

This PP2C member, which belongs to group A of the PP2C family and shares high sequence similarity with Arabidopsis HAB1 and HAB2 (Supplementary Fig. S2), was named *GhPP2C1* (GenBank ID: KP710220). The expression of *GhPP2C1* was evaluated in different organs of blooming plants. As shown in Fig. 4A, *GhPP2C1* was expressed in all tested organs, including cormels and corms. *GhPP2C1* was expressed throughout desiccation (weeks 0–6) and storage (weeks 6–14). The transcript levels began to decrease after harvest, and were lowest at the end of the desiccation period. However, the expression of *GhPP2C1* gradually increased after cold storage for CDR (Fig. 4B). This result is in accordance with the transcriptome data and suggests that *GhPP2C1* may regulate CDR.

Virus-induced gene silencing (VIGS) is widely used in functional analysis of horticultural plants, such as rose, apple, strawberry, and *Gladiolus* (Zhong *et al.*, 2014; Wu *et al.*, 2016; Ma *et al.*, 2017; S. Wang *et al.*, 2018). Therefore, we investigated the role of *GhPP2C1* in CDR using a VIGS approach. We inserted a specific 3'-untranslated region (UTR) fragment of *GhPP2C1* into the TRV2 vector for specific gene silencing in dormant cormels (Fig. 4C, D). After 10 d on soil, *GhPP2C*-silenced (*GhPP2C*-*TRV2*) cormels grew significantly more slowly than the control (empty TRV2 vector), and buds and roots were dramatically shorter than those of controls (Fig. 4C, E, F). These results indicate that down-regulation of *GhPP2C1* in dormant cormels leads to delayed CDR, demonstrating that GhPP2C1 acts as a positive regulator of CDR.

### GhNAC83 is a negative regulator of *GhPP2C1*

**Fig. 5.**
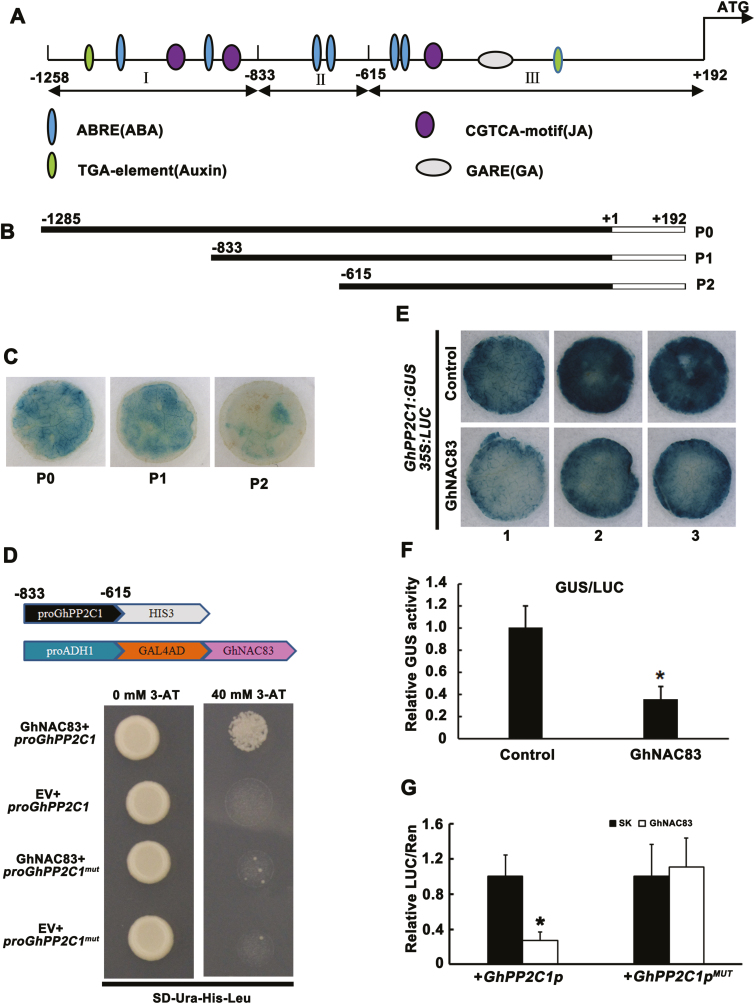
GhNAC83 binds the *GhPP2C1* promoter and inhibits its transcription. (A) The distribution of *cis*-elements in the *GhPP2C1* promoter. (B) Truncations of the *GhPP2C1* promoter used in transient assays. (C) Deletion of base pairs –833 to –615 in the *GhPP2C1* promoter (P2 construct shown in B) dramatically affects its activity in transient *N. benthamiana* assays. Three biological replicates were conducted and showed similar results. One biological replicate of leaf discs is shown. (D) The interaction of GhNAC83 and the *GhPP2C1* promoter in yeast one-hybrid assays. The empty prey vector (pDEST-GAD424) was used as the control. The mutagenesis of NAC-binding sites (Supplementary Fig. S3) in the *GhPP2C1* promoter (proGhPP2C1^mut^) was also tested. The interaction between the GhNAC83 protein and the *GhPP2C1* promoter was determined by cell growth on synthetic dropout nutrient medium lacking Ura, His, and Leu, and containing 40mM 3-AT. (E) GhNAC83 represses *GhPP2C1* promoter activity in transient expression assays in *N. benthamiana* leaves. 35S:GhNAC83 was used as an effector and *GhPP2C1*:GUS was used as a reporter. 35S:LUC was used as an internal control. GUS stains were performed on the third day post-infiltration. (F) The relative GUS activity (GUS/LUC) indicates that GhNAC83 represses *GhPP2C1* transcription *in planta*. Three biological replicates were performed and are shown with the SD. (G) The interaction of GhNAC83 with the *GhPP2C1* promoter using a dual-luciferase reporter assay in *N. benthamiana* leaves. A fragment of *GhPP2C1*’s promoter (base pairs +192 to –1285) was used in this assay. Mutated sites in *GhPP2C1p*^*MUT*^ are shown in Supplementary Fig. S3. The empty vector (pGreenII 62-SK) was used as a control. Data are shown as the average of three biological replicates with the SD (*n*=5 leaves), *P<0.05. (This figure is available in color at *JXB* online.)

To explore the regulation of *GhPP2C1* during CDR further, we isolated a 1.5 kb sequence of the *GhPP2C1* regulatory region upstream of the translation start site (Fig. 5A) by Hi-TAIL PCR. Based on the distribution of *cis*-elements, we truncated the promoter (Fig. 5B) and performed transient expression assays in leaves of *N. benthamiana*. Our results show that the promoter activity is unaffected when region I is deleted (–1285 to –833; P1 construct); however, a deletion in region II (–833 to –615; P2 construct) led to a sharp decrease in promoter activity (Fig. 5C). Therefore, we focused our efforts on identifying regulators that bind region II of the *GhPP2C1* promoter.

The 219 bp region II contains several conserved TF-binding sites (Supplementary Fig. S3A). To identify TFs that bind this region of the *GhPP2C1* promoter, a yeast one-hybrid screen was performed using a TF library from Arabidopsis (Mitsuda *et al.*, 2010). First, we selected yeast harboring the integrated 219 bp promoter that could not survive on selection medium containing 40 mM 3-AT. Then, we performed the yeast one-hybrid screen and isolated 12 TFs among 100000 cfu (Table 1). We then identified *Gladiolus* homologous genes using the *Gladiolus* transcriptome database, and five TFs were able to bind region II (Table 1). Taking into consideration the expression level during CDR and the number of clones identified from the yeast one-hybrid screen (Table 1), *GhNAC83* (*GlaUn057212*) was selected for further study.

**Table 1.** Potential upstream regulators of GhPP2C1 screened by yeast one-hybrid analysis

Gene	Family	Repeats^*a*^	Re-Y1H^*b*^	DD	WD	ED
GhNAC83	NAC	42	+	**60.55**	**28.08**	**7.32**
GhbZIP1	bZIP	28	+	*22.98*	*58.48*	*74.44*
*GhWRKY40*	WRKY	16	+	**331.05**	*347.76*	**93.83**
*GhMYB1R1*	MYB	12	+	*26.29*	*33.28*	*49.16*
*GhAPL-Like*	MYB	4	+	**106.24**	**83.66**	**53.97**
*GhDof1.8*	Dof	18	–	6.56	4.28	7.12
*GhbZIP60*	bZIP	14	–	23.12	36.86	59.18
*GhBPC1*	BPC	12	–	1.19	1.09	3.40
*GhCIB4*	bHLH	12	–	–	–	–
*GhWOX6*	HB	11	–	–	–	–
*GhTCP4*	TCP	10	–	17.69	17.12	12.51
*GhKNU*	C2H2	1	–	–	–	–

^*a*^ Number of colonies harboring the same gene isolated by yeast one-hybrid (Y1H) screens using an Arabidopsis TF library (Mitsuda *et al.*, 2010).

^*b*^ Positive or negative Y1H results when using *Gladiolus* clones.

DD, deep dormancy; WD, weak dormancy; ED, ecodormancy. The data correspond to the expression level (based on FPKM evaluation) in the *Gladiolus* transcriptome database. Values in bold indicate down-regulation and those in italics indicate up-regulation.

To test the binding ability of GhNAC83, we mutated the NAC-binding site in the promoter (Supplementary Fig. S3B). The result showed that GhNAC83 binds the native *GhPP2C1* promoter, but cannot bind the mutant promoter (Fig. 5D). In addition, to test further the effect of GhNAC83 on *GhPP2C1* transcription, we performed transient transactivation assays using the *GhPP2C1* promoter driving GUS reporter expression. A GhNAC83 effector construct driven by the 35S promoter was co-agroinfiltrated with the reporter construct into leaves of *N. benthamiana*. The expression of *GhPP2C1* was significantly inhibited by GhNAC83 (Fig. 5E, F). Furthermore, a dual-LUC reporter assay was performed to analyze the regulation of the *GhPP2C1* promoter by GhNAC83 *in planta*. The results show that GhNAC83 decreases *GhPP2C1* promoter activity; furthermore, when we mutated the binding sites of GhNAC83 in the *GhPP2C1* promoter (*GhPP2C1p*^*MUT*^), *GhPP2C1*^*MUT*^ promoter activity was unaffected (Fig. 5G). These results suggest that GhNAC83 directly binds the *GhPP2C1* promoter and negatively regulates its expression *in planta*.

### *GhNAC83* is down-regulated during corm dormancy release

**Fig. 6.**
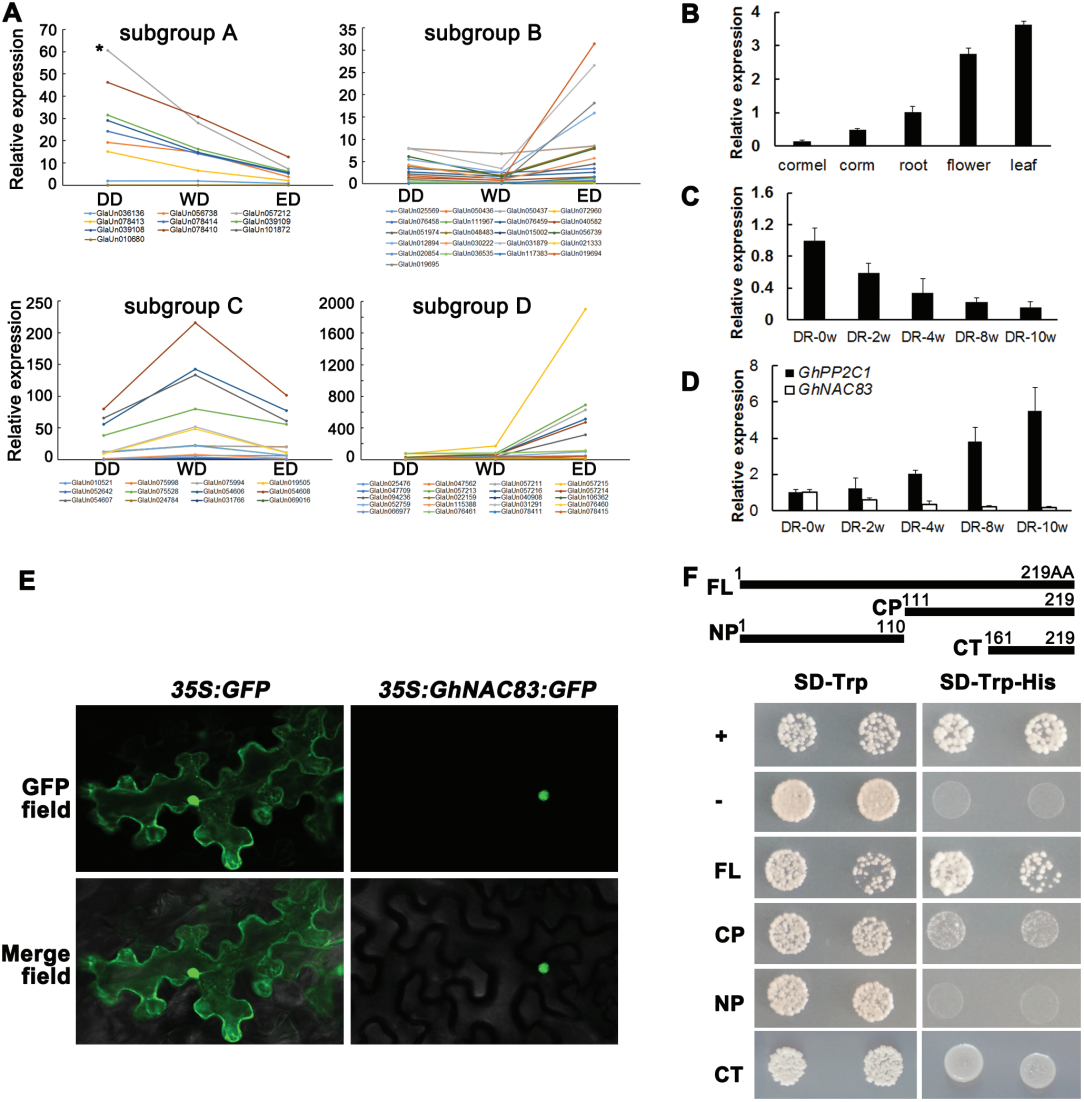
The expression pattern and protein characteristics of GhNAC83. (A) Expression pattern of *GhNAC* genes in the transcriptome database of *Gladiolus* CDR. The expression levels are based on a FPKM evaluation. The asterisk (*) represents *GhNAC83*. (B) The expression of *GhNAC83* in different organs during the blooming stage. (C) The expression pattern of *GhNAC83* during CDR. (D) The expression pattern of *GhNAC83* shows a negative relationship with *GhPP2C1* during CDR. Three biological replicates with the SD were performed. (E) Subcellular localization of GhNAC83 in the epidermal cells of *N. benthamiana*. (F) Transactivation activity of GhNAC83 in yeast. Yeast cells were grown on synthetic dropout nutrient medium lacking Trp (SD-Trp)and Trp/His (SD-Trp-His). DR, dormancy release; FL, full length; CP, C-terminal part; NP, N-terminal part; CT, C-terminus. (This figure is available in color at *JXB* online.)

To better understand the function of GhNAC83, we analyzed its sequence and expression patterns. *GhNAC83* encodes 219 amino acids with high similarity to NAC83-like in other species and VNI2 (VASCULAR-RELATED NAC-DOMAIN INTERACTING2) in Arabidopsis, and belongs to subgroup II VIII-3 cluster ( Ooka *et al.*, 2003; Jensen *et al.*, 2010) (Supplementary Fig. S4A), containing five conserved domains (A–E) (Supplementary Fig. S4B). In addition, GhNAC83 contains a transcription repressor motif ‘LVFY’ (Puranik *et al.*, 2012; Wang *et al.*, 2017). In our transcriptome database, GhNAC83 is one of 10 GhNACs down-regulated during CDR (Fig. 6A). GlaUn078410 (red line in subgroup A of Fig. 6A) showed a similar trend to GhNAC83, and had high sequence similarity to ATAF1 in Arabidopsis and OsNAC6 in rice. ATAF1 and OsNAC6 have been shown to participate in ABA signaling (Nakashima *et al.*, 2007; Ton *et al.*, 2009).

At the blooming stage, *GhNAC83* had high expression in leaves, flowers, and roots, and had low expression in cormels (Fig. 6B). In addition, the expression of *GhNAC83* gradually decreased during the cold storage required for CDR (Fig. 6C). During dormancy release stages, the expression pattern of *GhNAC83* was almost opposite to that of *GhPP2C1* (Figs 4B, 6D). These results are in accordance with the results *in planta* which showed that *GhNAC83* negatively regulates *GhPP2C1* expression during CDR (Fig. 5E–G).

To provide evidence for potential roles of *GhNAC83* in transcriptional regulation, we examined the subcellular localization of GhNAC83 in *N. benthamiana* epidermal cells. The results showed that the GhNAC83–GFP fusion protein localizes to the nucleus (Fig. 6E). Additionally, a transactivation activity assay was performed in yeast to examine the transactivation ability of GhNAC83. On selection medium, yeast colonies harboring pGAL4 (positive control), FL (full length), CP (C-terminal part), or CT (C-terminus) grew, whereas colonies harboring pBD (negative control) or NP (N-terminal part) did not grow (Fig. 6F). These data suggest that GhNAC83 contains a transactivation domain in its C-terminal region between amino acids 161 and 219. GhNAC83 contains a transcriptional repressor domain in the NP (LVFY; amino acids 105–108) in addition to a transactivation domain, suggesting that GhNAC83 is a bifunctional TF, similar to its homologous gene (VNI2) in Arabidopsis (Yang *et al.*, 2011).

### Silencing of *GhNAC83* accelerates corm dormancy release

**Fig. 7.**
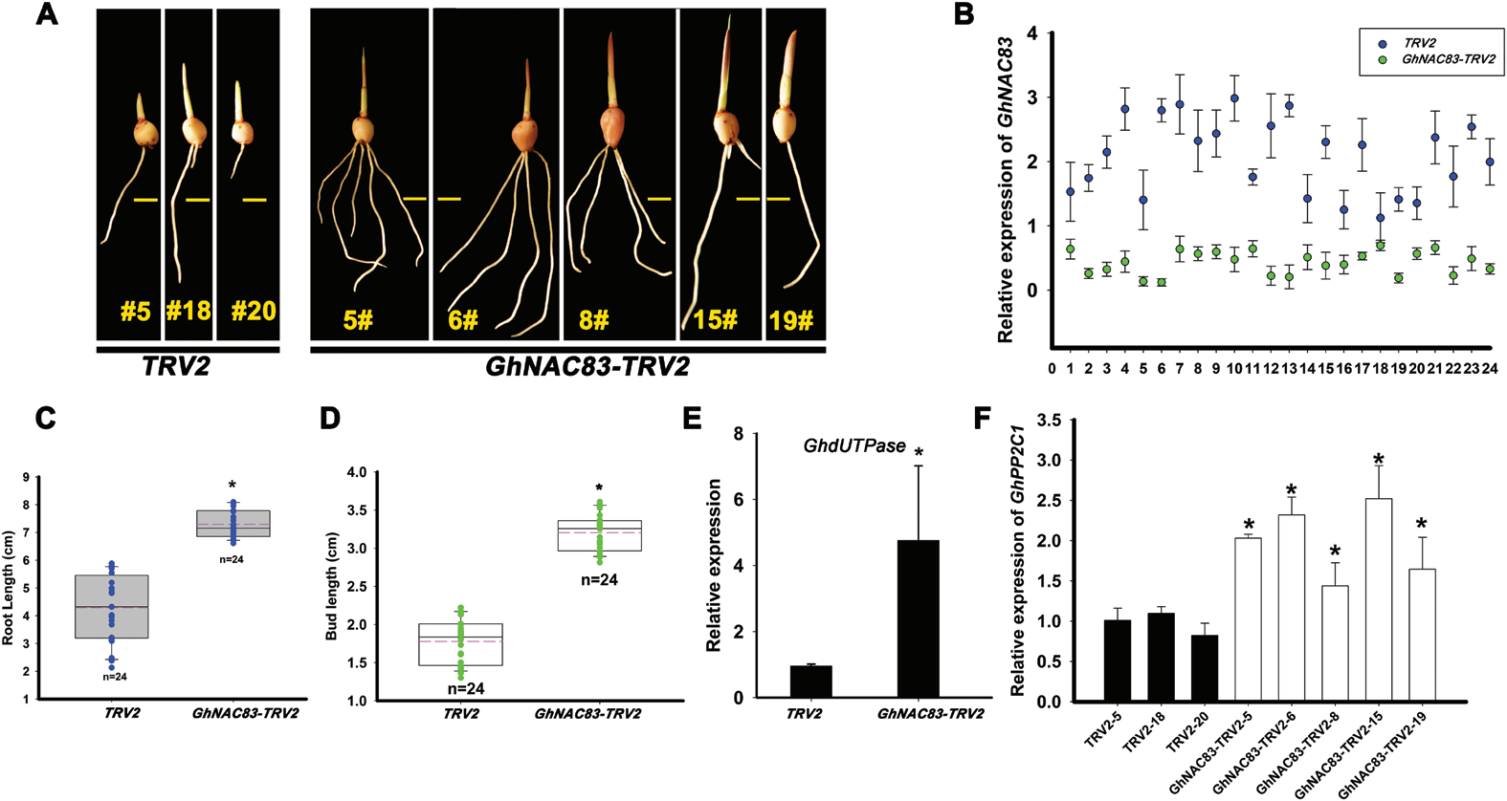
Silencing of *GhNAC83* accelerates corm dormancy release. (A) The phenotype of *GhNAC83-TRV2* independently silenced lines compared with *TRV2* control lines 10 d after planting on soil. The scale bar represents 1 cm. (B) The expression of *GhNAC83* in 24 independent *GhNAC83-TRV2* lines. Data are shown as averages of three technical replicates with the SD. The root length (C) and bud length (D) in *GhNAC83-TRV2* and *TRV2* lines. Data are shown as averages of *n*=24 independent lines with the SD. (E) Expression of the dormancy release marker *GhdUTPase* in *GhNAC83-TRV2* and *TRV2* lines. (F) Expression of *GhPP2C1* in five independent *GhNAC8*-silenced lines. Data are shown as averages of three technical replicates with the SD (*P<0.05; **P<0.01). (This figure is available in color at *JXB* online.)

Since there is no report about NAC members participating in corm dormancy, we conducted VIGS in order to evaluate the potential role of *GhNAC83* in *Gladiolus* CDR. Accelerated sprouting occurred when *GhNAC83* was silenced in dormant cormels (Fig. 7A, B). Roots and buds of *GhNAC83-TRV2* were also significantly longer than those of the *TRV2* control (Fig. 7C, D). It has been shown that *UTPase* is a marker for tuber dormancy release in *Solanum tuberosum* (Senning *et al.*, 2010; Hartmann *et al.*, 2011). Here, the expression of a CDR marker (*GhdUTPase*) was dramatically higher in silenced lines than in the control, a result that confirmed the observed phenotype (Fig. 7E). To confirm that silencing of *GhNAC83* can affect transcription of *GhPP2C1*, we determined the expression level of *GhPP2C1* in *GhNAC83* silenced lines. The results showed that the expression of *GhPP2C1* was higher in *GhNAC83* silenced lines (Fig. 7F). Together with the binding assay results in Fig. 5, our data suggest that GhNAC83 negatively regulates *GhPP2C1* expression and inhibits CDR.

### GhNAC83 mediates CK biosynthesis by directly targeting the *GhIPT* promoter

**Fig. 8.**
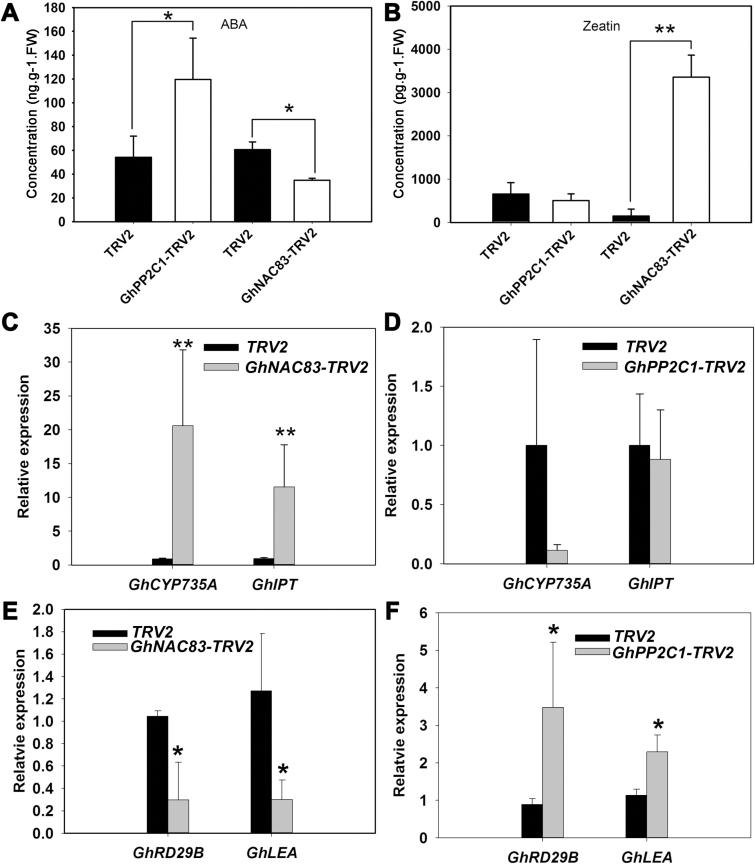
GhNAC83 mediates CK biosynthesis during CDR in a *GhPP2C1*-independent manner. (A) ABA levels in *GhNAC83*- or *GhPP2C1*-silenced lines. (B) Zeatin levels in *GhNAC83*- or *GhPP2C1*-silenced lines. (C) The expression of important CK biosynthesis genes in *GhNAC83*-silenced lines. (D) The expression of important CK biosynthesis genes in *GhPP2C1*-silenced lines. (E) Expression of genes downstream from GhABI5 in *GhNAC83*-silenced lines. (F) Expression of genes downstream from GhABI5 in *GhPP2C1*-silenced lines. Averages of three biological replicates with the SD are shown (*P<0.05; **P<0.05). Five independent silenced lines and five independent control lines were used.

To investigate further how *GhPP2C1* and *GhNAC83* affect CDR, we measured endogenous phytohormone levels in *GhNAC83*-silenced cormels. *GhNAC83*-silenced cormels had half the ABA content and a dramatic increase in zeatin level compared with TRV2 cormels (Fig. 8A, B). This result suggests that GhNAC83 may be involved in the antagonism between ABA and CKs during CDR. In *GhPP2C1-TRV2* cormels, zeatin was unaffected compared with TRV2 cormels (Fig. 8B). Moreover, the expression of CK biosynthesis gene homologs (*GhCYP735A* and *GhIPT*) was dramatically higher in *GhNAC83-TRV2* lines and there was no difference in *GhPP2C1-TRV2* lines when compared with TRV2 lines (Fig. 8C, D). Therefore, it is likely that GhNAC83 mediates zeatin biosynthesis during CDR independently of *GhPP2C1*. As for ABA, silencing of *GhNAC83* decreased the expression of genes downstream of *ABI5* (*GhRD29B* and *GhLEA*) and silencing of *GhPP2C1* led to an opposite result (Fig. 8E, F). Overall, these results suggest that GhNAC83 can influence ABA signaling responses through *GhPP2C1*, and that GhNAC83 affects ABA and zeatin levels in an antagonistic manner during CDR.

**Fig. 9.**
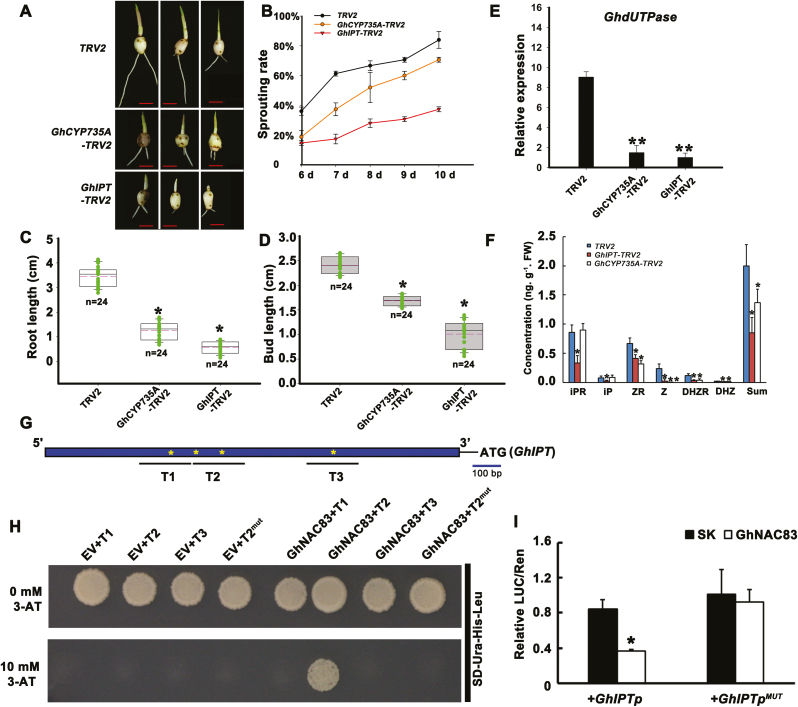
Silencing of cytokinin biosynthesis genes delays corm dormancy release. (A) Phenotypes associated with silencing of *GhCYP735A* and *GhIPT* in dormant cormels 10 d after planting on soil; scale bar=1 cm. (B) The sprouting rate of control (TRV2) and silenced cormels. Cormels were considered sprouted once bud length was longer than 0.5 cm. Averages of three biological replicates with the SD are shown (*n*=24 independent lines). Root length (C) and bud length (D) of the *TRV2* control and *GhCYP735A* /*GhIPT-TRV2* silenced cormels (*n*=24 independent lines). (E) Transcript levels of the dormancy release marker *GhdUTPase* in *TRV2* and silenced cormels. Data are shown as averages of four independent lines with the SD. (F) Concentration of endogenous CKs in *TRV2* and *GhCYP735A*/*GhIPT-TRV2* cormels. iPR, isopentenyladenosine; iP, isopentenyladenine; Z, zeatin, ZR: zeatin riboside; DHZ, dihydrozeatin; DHZR, dihydrozeatin riboside; Sum, total amount of iPR, iP, Z, ZR, DHZ, and DHZR. Data are shown as averages of three independent lines with the SD. (G) Schematic representation of the *GhIPT* upstream regulatory region. Asterisks (*) correspond to putative NAC-binding sites and the lines indicate the fragments used in the yeast one-hybrid analysis shown in (H). T1, base pairs –1140 to –940; T2, base pairs –910 to –710; T3, base pairs –500 to –300 relative to the translation start site of *GhIPT*. (H) The interaction of GhNAC83 with the *GhIPT* promoter in yeast. EV, empty prey vector (pDEST-GAD424). The interaction between protein and promoter was determined by yeast growth on synthetic dropout nutrient medium lacking Ura, His, and Leu, and containing 10mM 3-AT. The mutagenesis of NAC-binding sites (Supplementary Fig. S5) in the *GhIPT* promoter (T2^mut^) was also tested. (I) The interaction of GhNAC83 with the *GhIPT* promoter using a dual-luciferase reporter assay in *N. benthamiana* leaves. A fragment of the *GhIPT* promoter (base pairs +56 to –1537) was used in this assay, and mutant sites in *GhIPTp*^*MUT*^ are shown in Supplementary Fig. S5. The empty vector (pGreenII 62-SK) was used as a control. Data are shown as the average of three biological replicates with the SD (*n*=5 leaves) (*P<0.05 and **P<0.01). (This figure is available in color at *JXB* online.)

Presently, there is limited information concerning the relationship between CK biosynthesis and CDR. To test the role of CK biosynthesis genes *GhCYP735A* and *GhIPT* in CDR, VIGS was employed. The results showed that silencing *GhCYP735A* or *GhIPT* in dormant cormels led to delayed sprouting (Fig. 9A, B). The buds and roots of silenced cormels (*GhCYP735A* and *GhIPT*) were significantly shorter than those of the *TRV2* control (Fig. 9C, D). Moreover, expression of the CDR marker, *GhdUTPase*, was dramatically lower than that of *TRV2* (Fig. 9E). To test whether a decrease in CK levels was indeed responsible for CDR, we measured the levels of active forms of CKs in silenced cormels. We focused on active CKs as they function in CK signal transduction, and they can mainly reflect CK changes in plants (Wang *et al.*, 1995; Yong *et al.*, 2000; Werner *et al.*, 2001; Aoyama and Oka, 2003; Yonekura-Sakakibara *et al.*, 2004; Ashikari *et al.*, 2005; Tao *et al.*, 2010; Matsuo *et al.*, 2012). Silencing of *GhCYP735A* decreased the levels of zeatin (free and riboside) and dihydrozeatin (free and riboside) in cormels (Fig. 9F). Furthermore, silencing of *GhIPT* also decreased the level of isopentenyladenosine and isopentenyladenine in cormels (Fig. 9F). These results indicate that silencing CK biosynthesis genes (*GhCYP735A* and *GhIPT*) reduces active CK contents in cormels and further inhibits CDR.

To determine whether GhNAC83 can directly regulate the expression of CK biosynthesis genes, we cloned a 1594 bp upstream sequence of *GhIPT* by Hi-TAIL PCR. There are four predicted NAC-binding sites in the *GhIPT* promoter (Fig. 9G). Using a yeast one-hybrid approach, we found that GhNAC83 binds the T2 fragment (base pairs –910 to –710) of the *GhIPT* promoter, while mutation of NAC-binding sites in the T2 fragment (Supplementary Fig. S5) resulted in no binding ability (Fig. 9H). In addition, we performed a LUC reporter assay to analyze the regulation of *GhIPT* expression by GhNAC83 *in planta* (Fig. 9I). *Nicotiana benthamiana* leaf cells co-transformed with *35S:GhNAC83* and *GhIPT:LUC* exhibited significantly lower LUC activity than cells transformed with empty vector and *GhIPT:LUC*. The activity of the *GhIPT* promoter with mutated NAC-binding sites in the T2 region (*GhIPTp*^*MUT*^) was no longer significantly different between treatments with empty vector and *35S:GhNAC83* (Fig. 9I). These data suggest that GhNAC83 negatively regulates *GhIPT in planta*.

**Fig. 10.**
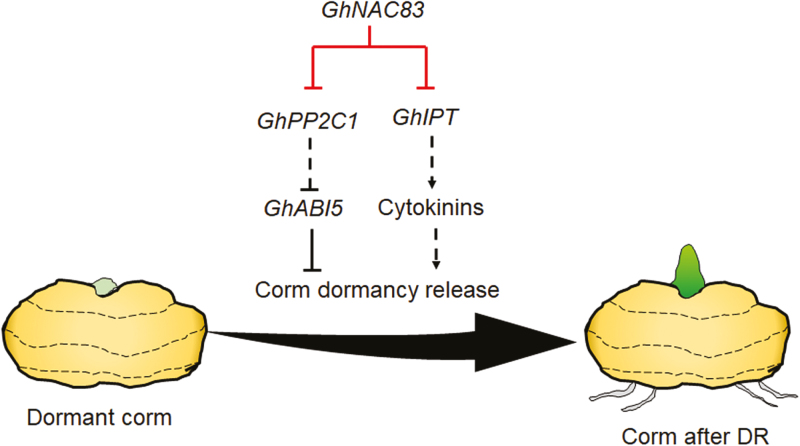
Diagram of *GhNAC83* in regulating corm dormancy release in *Gladiolus*. GhNAC83 directly binds *GhPP2C1* and *GhIPT* promoters and represses their expression, modulating ABA signaling and CK biosynthesis during CDR. (This figure is available in color at *JXB* online.)

Together, our results demonstrate that GhNAC83 directly binds the *GhIPT* promoter and negatively regulates CK biosynthesis in a *GhPP2C1*-independent manner to control CDR in *Gladiolus* (Fig. 10).

## Discussion

In this study, we identified a novel NAC family member in *Gladiolus*, GhNAC83, and characterized its negative regulatory role in CDR. *GhNAC83* expression decreases during CDR. GhNAC83 directly binds to and inhibits *GhPP2C1* expression, thereby activating ABA downstream response, and additionally GhNAC83 inhibits CK biosynthesis through direct binding and down-regulation of *GhIPT*, thus delaying CDR (Fig. 10). Accordingly, silencing of *GhNAC83* in dormant cormels leads to a higher zeatin content and lower ABA levels, thereby promoting CDR, while silencing of *GhPP2C1* results in delayed CDR by enhancing ABA downstream response. Altogether, the data shown here indicate that *GhNAC83* regulates ABA–CK crosstalk to inhibit CDR.

### GhPP2C1 promotes CDR

Plant dormancy is a complex trait and is regulated by several phytohormones, with ABA being a central player (Finkelstein *et al.*, 2008). In Arabidopsis, PP2Cs are sorted into 10 subgroups (A–J) and have been shown to play a role in diverse signaling pathways related to plant development and stress responses (Kerk *et al.*, 2002). Members of subgroup A, including ABI1/2 and HAB1/2, are co-receptors of ABA signaling, and regulate seed germination and abiotic stress (Gosti *et al.*, 1999; Kong *et al.*, 2015).

Here, we isolated *GhPP2C1*, which belongs to subgroup A and shares high sequence similarities with HAB1 and HAB2 (Supplementary Fig. S2), in addition to conserved motifs, such as the PYL interaction site, PA (phosphatidic acid)-binding site, metal contact points, and a nuclear localization signal (NLS)-like motif at the C-terminus (Supplementary Fig. S6) (Moes *et al.*, 2008; Zhang *et al.*, 2014). *GhPP2C1* was differentially expressed in a transcriptomic analysis of *Gladiolus* during CDR. The transcription of *GhPP2C1* increases during CDR in *Gladiolus*, and further functional analysis showed that silencing of *GhPP2C1* results in delayed CDR by enhancing ABA downstream response (Fig. 8F). Together with the transcriptome analysis data (Supplementary Table S3), our results present a role for the clade A PP2C, GhPP2C1, as a positive regulator of CDR.

### GhNAC83 plays a role in ABA–CK crosstalk to inhibit CDR

Yeast one-hybrid screening is widely used for the identification of TFs that bind a specific *cis*-element in the promoter of a gene of interest. Also, employing this technique allows us to use a TF-specific library which is much more convenient than a traditional cDNA library given that it reduces false positives, enriches full-length TFs, and overall has higher efficiency (Mitsuda *et al.*, 2010). Therefore, we performed yeast one-hybrid screening with an Arabidopsis TF library and identified homologs in *Gladiolus* from these results. We then confirmed the results by performing yeast one-hybrid analysis with the homologous TFs, proving the interaction with the *GhPP2C1* promoter. One TF, GhNAC83, had the highest affinity for the *GhPP2C1* promoter, and further analysis by transient reporter activation assays showed that GhNAC83 acts as a negative regulator of *GhPP2C1* (Fig. 5E–G). These data are in accordance with the expression of *GhPP2C1* in *GhNAC83*-silenced cormels (Fig. 7F).

NACs are a large TF family in plants and are associated with diverse processes. Despite their highly conserved DNA-binding domains, the remarkable diversification across plant species reflects their numerous functions (Puranik *et al.*, 2012). Here, we identified a novel NAC member, GhNAC83, which has high similarity to NAC83 from *Asparagus officinalis*. Similar to its homologous protein (VNI2) in Arabidopsis, which functions in integrating ABA signaling into leaf senescence via the *COLD-REGULATED* (*COR*)/*RD* genes (Yang *et al.*, 2011), GhNAC83 contains a transactivation domain in its C-terminal region and is localized to the nucleus (Fig. 6E, F). Further study of GhNAC83 showed that *GhNAC83* is down-regulated during CDR and has an opposite expression pattern to *GhPP2C1* (Fig. 6C, D). Silencing *GhNAC83* results in earlier CDR with longer buds and roots (Fig. 7A, C, D), showing that NAC family members also contribute to the process of CDR in plants.

Previous studies have shown that NACs modulate drought stress, oxidative stress, and leaf senescence by regulating the expression of some PP2C family members (Zhang and Gan, 2012; You *et al.*, 2014). In particular, in rice, SNAC1 (STRESS-RESPONSIVE NAC1) directly regulates *OsPP18* (*PROTEIN PHOSPHATASE18*) and confers drought and oxidative stress tolerance by regulating ROS (reactive oxygen species) homeostasis through ABA-independent pathways. Although OsPP18 belongs to the PP2C family (subgroup F), ABA response genes and ABA sensitivity were not affected in the *ospp18* mutant (You *et al.*, 2014). In this study, we found that GhNAC83 negatively regulates the expression of *GhPP2C1* (Fig. 7F) and up-regulates the expression of ABA-responsive genes (*GhRD29B* and *GhLEA*; Fig. 8E), indicating that GhNAC83 regulates CDR in an ABA-dependent pathway.

Previous research has shown that some NAC family members participate in ABA pathways, as explained above, and some NAC family members participate in CK pathways, such as NTM1, which is activated by proteolytic cleavage through regulated intramembrane proteolysis and tightly mediates CK signaling during cell division in Arabidopsis (Kim *et al.*, 2006). In this study, we show that GhNAC83 is involved in both ABA (above) and CK pathways. GhNAC83 is a nuclear protein that negatively regulates *GhIPT* expression, inhibiting CK biosynthesis and resulting in partial repression of CDR. Given the large size of the NAC TF family, it will be interesting in the future to test if different NACs can integrate different environmental and endogenous signals to regulate growth rates in cormels and other organs by balancing ABA and CK levels and signaling.

### Corm and seed dormancy release

Corm and seed dormancy release are two processes with similarities and differences. Seed dormancy release is regulated by two major hormones: ABA and GA (Finch-Savage and Leubner-Metzger, 2006). On the other hand, *Gladiolus* corm dormancy release is regulated by CKs and ABA. Moreover, previous research has shown that GA is not an essential hormone in promoting CDR in *Gladiolus* (Ginzburg, 1973). This research is in accordance with our transcriptome analysis, where we showed that GA-related DEGs are not in the top three of hormone metabolism-related DEG abundance (Supplementary Fig. S1C, D). Instead, ABA- and CK-related DEGs are enriched, suggesting that CKs may play a more prominent role than GA in *Gladiolus* CDR, and not GA, but the molecular mechanism is still largely unknown (Ginzburg, 1973; Wu *et al.*, 2015). Another difference in corm and seed dormancy is that corms lack seed coats and an endosperm; therefore, due to these structural differences, corms do not undergo coat and endosperm dormancy as seeds do. Thus, factors related to coat or endosperm dormancy do not affect corm dormancy (Finch-Savage and Leubner-Metzger, 2006). Given that hormone crosstalk plays a major role in regulating seed dormancy, with most hormones contrasting the inhibitory role of ABA (Gazzarrini and Tsai, 2015; Shu *et al.*, 2016), it will be interesting in the future to characterize the interaction between ABA, CK, and other hormones such as auxin in *Gladiolus* CDR.

## Supplementary data

Supplementary data are available at *JXB* online.

Table S1. Primer sequences used in this study.

Table S2. Overlapping differentially expressed genes in deep, weak, and ecodormancy.

Table S3. List of differentially expressed cytokinin- and ABA-related genes.

Fig. S1. Gene Ontology (GO) enrichment analysis.

Fig. S2. Alignment of GhPP2C1 with the PP2C family in Arabidopsis.

Fig. S3 Predicted transcription factor-binding sites in the *GhPP2C1* promoter (–833 to –615 bp).

Fig. S4. Phylogenetic analysis of GhNAC83.

Fig. S5. Mutagenesis of NAC-binding sites in the *GhIPT* promoter.

Fig. S6. Sequence alignment of GhPP2C1 with its homologs.

Supplementary Figures S1-S6 Table S1

Supplementary Table S2

Supplementary Table S3
