# Supplementary material for: GhNAC83 inhibits corm dormancy release by regulating ABA signaling and cytokinin biosynthesis in Gladiolus hybridus
Source: J Exp Bot. 2018 Dec 5;70(4):1221–37. doi: 10.1093/jxb/ery428 (PMC6382327; doi:10.1093/jxb/ery428)
Supplement: Supplementary Figures S1-S6 Table S1 [file ery428_suppl_supplementary_figures_s1-s6_table_s1.pdf]

SUPPLEMENTARY DATA

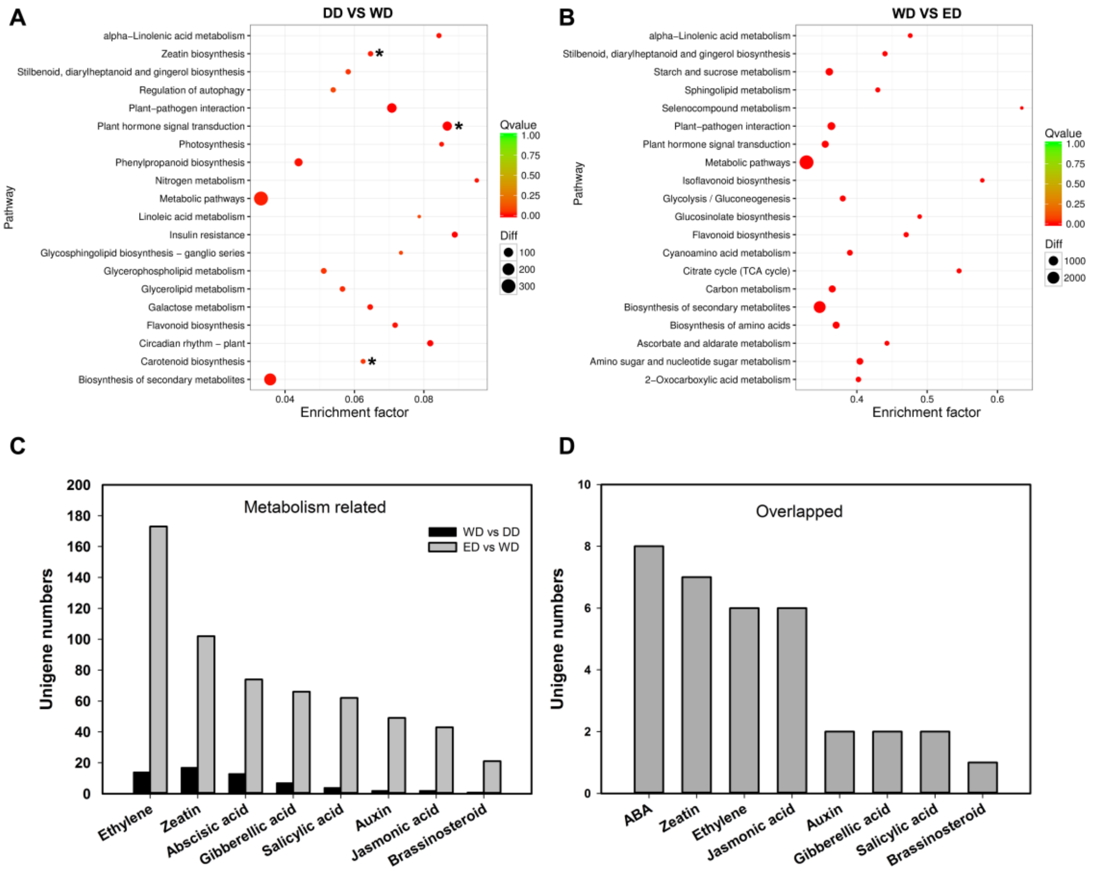

Figure S1 Gene Ontology (GO) enrichment analysis. GO-term enrichments in WD (weak dormancy) versus DD (deep dormancy) (A) and ED (ecodormancy) versus WD (B) were generated using BLAST2GO. Only the top 20 most strongly represented pathways were displayed in the diagram. The degree of pathway enrichment was represented by an enrichment factor, the Qvalue, and the number of unigenes enriched in the pathway (Diff). The enrichment factor indicates the ratio of differential expression unigenes enriched in this pathway to the total number of annotated unigenes in this pathway. The names of the pathways are listed along the y-axis. The Qvalue indicates the corrected P-value, ranging from 0 and 1, and a Qvalue closer to 0 indicates more enrichment. C, The distribution of hormone metabolism DEGs (differentially expressed genes) during dormancy release. D, Overlapped DEGs of Hormone metabolism in WD vs DD and ED vs WD. DEGs when a cutoff ratio of Log2 <-1 or >1, and q value <0.05.

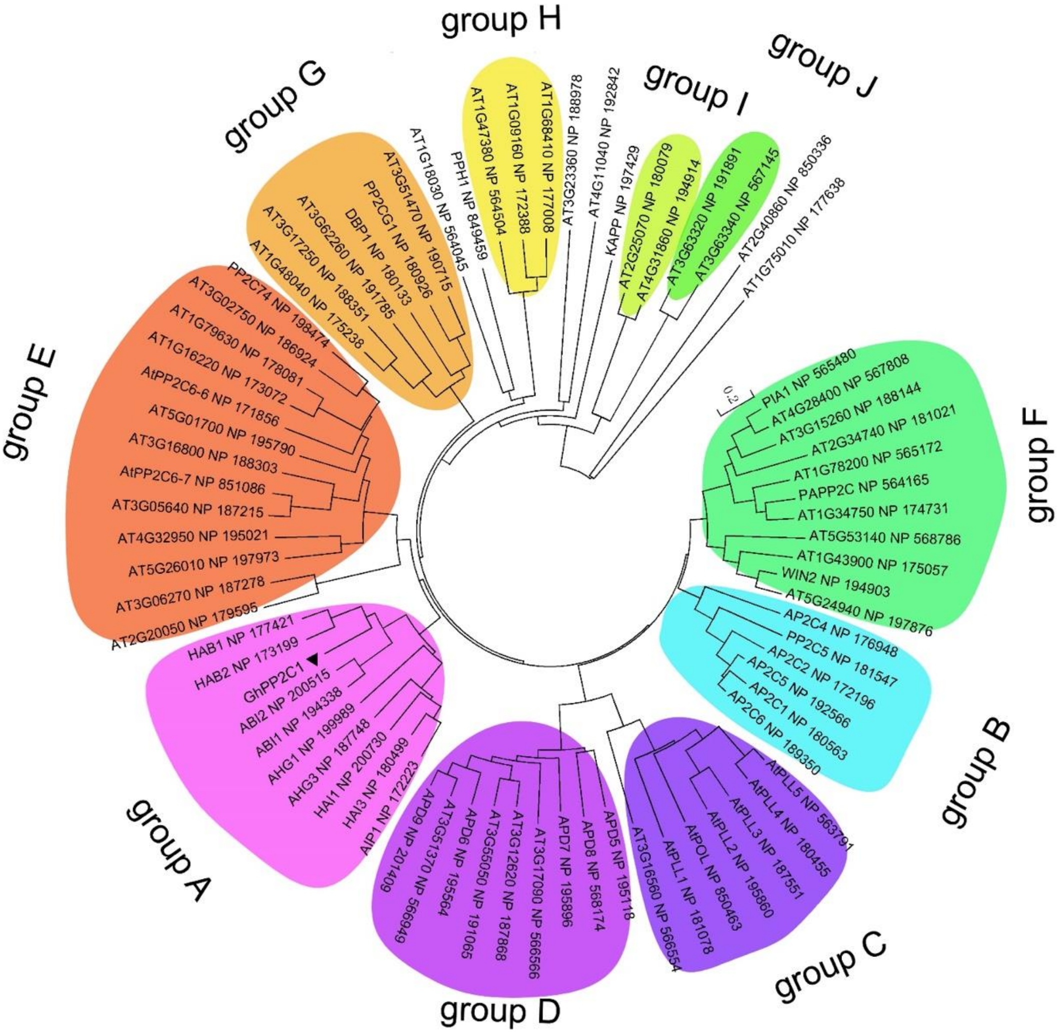

Figure S2 Alignment of GhPP2C1 with PP2C family in Arabidopsis. Phylogenetic relationships among GhPP2C1 (black triangle) and Arabidopsis PP2C proteins. GhPP2C1 belongs to PP2C1 group A.

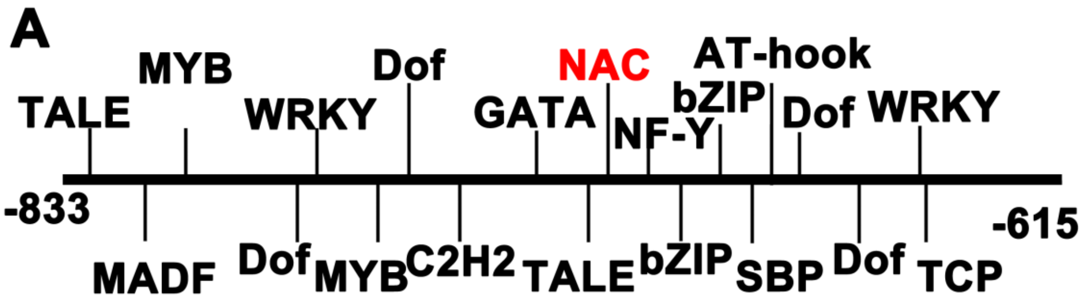

B

GhPP2C1 promoter sequence for Y1H:

CTCTGACAGGGACGTTAACCCCGCACGCCGTTAAACATAAGTGGGCGCCGCTTTTCCTGACTGGGTGATTCCATGGGTG
AAAGGTTTCCGGTGCACTACGGAGAAGAGAAGATCGTGACATGTGCTAAATTGGGGTGGACACGTACGCATAAATACTT
TAAATGCGCTATTGCTGGCGGAGTTTACAAAGACAAGTCAGGGCCCCACCAGAGCTAACG

GhPP2C1 mutant promoter seuqence:

CTCTGACAGGGACGTTAACCCCGCACGCCGTTAAACATAAGTGGGCGCCGCTTTTCCTGACTGGGTGATTCCATGGGTG
AAAGGTTTCCGGgGCcCTACGGAGAAGAGAAGATCccGACcTtTGCTAAATTGGGGTGGACACGTACGCATAAATACTTT
AAATGCGCTATTGCTGGCGGAGTTTACAAAGACAAGTCAGGGCCCCACCAGAGCTAACG

NAC binding site   mutant site

Figure S3 Predicted transcription factor binding sites in the GhPP2C1 promoter (-833 to -615 bp) for Yeast one hybrid. A. Predicted transcription factor binding sites. B. Mutagenesis of NAC binding site in the GhPP2C1 promoter.

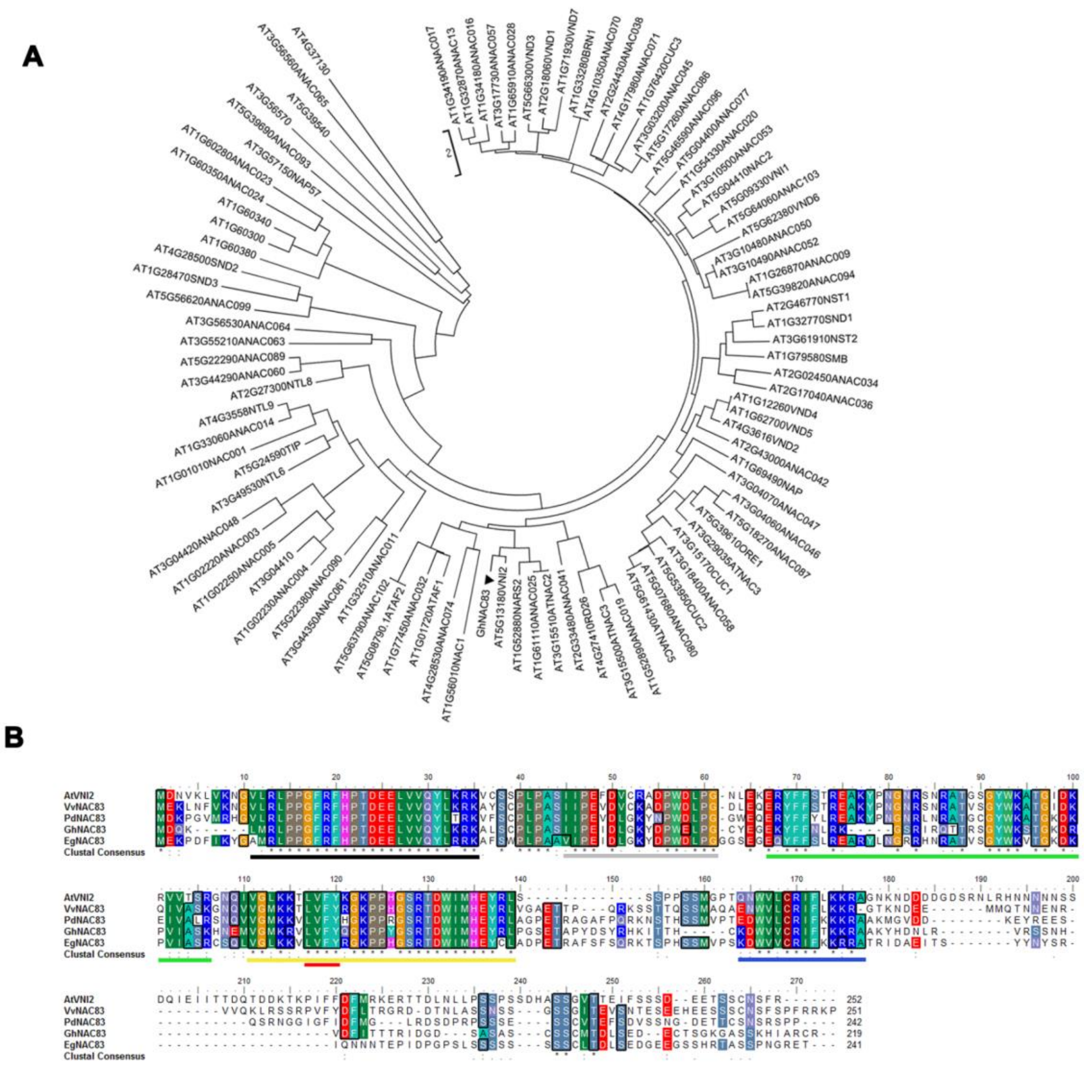

Figure S4 Phylogenetic analysis of GhNAC83. A, Phylogenetic relationships among GhNAC83 (black triangle) and Arabidopsis NAC proteins. B, Sequence alignment of GhNAC83 with its homologs from *Arabidopsis thaliana* (AtVNI2), *Vitis vinifera* (VvNAC83; XP_002264588), *Phoenix dactylifera* (PdNAC83; XP_010907664) and *Elaeis guineensis* (EgNAC83; XP_010909195). The conserved A to E domains are indicated by a black, gray, green, yellow and blue lines, respectively. The red line indicates the 'LVFY' repression motif.

GhIPT promoter T2 sequence for Y1H:

TCGTATAGCTTTCCGACTTCAAATACTCTCATTCAACAATGGCATTGCTTGGCACAC
CGGCTACTAACACAAGCTCTTCAAACTTTTTGGAAGAACTAAACAGTCCACCTTCAC
GCATGAACTTGCCAACATAAAACTAAAACGAGGTTTACTTCAATTTTGCTTTTGGAGT
GATGTTGTAACAGTATAACTAATCATAG

GhIPT mutant promoter T2 sequence:

TCcTATAGCTTTCCGACTTCAAATACTCTCATTCAACAATGGCATTGCTTGGCACAC
CGGCTACTAACACAAGCTCTTCAAACTTTTTGGAAGAACTAAACAGTCCACCTTCcC
GCATGAACTTGCCAACATAAAACTAAAACGAGGTTTACTTCAATTTTGCTTTTGGAGT
GATGTTGTAACAGTATAACT

Figure S5 Mutagenesis of NAC binding site in the GhIPT promoter.

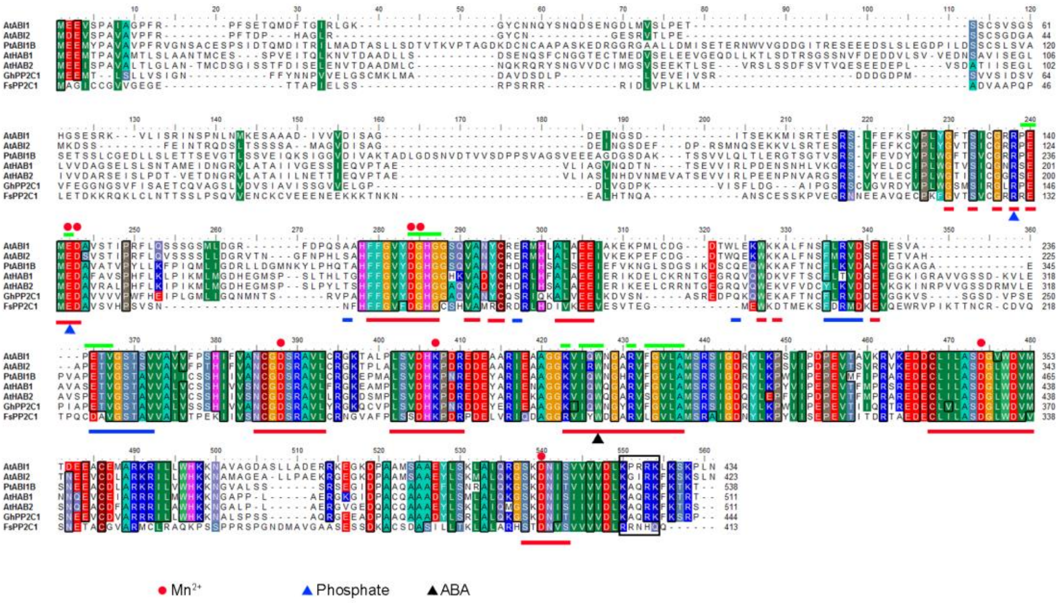

Figure S6 Sequence alignment of GhPP2C1 with its homologs. Alignment of GhPP2C1 with four group A members in Arabidopsis (AtABI1, AtABI2, AtHAB1 and AtHAB2), FsPP2C1 (*Fagus sylvatica*; CAB90633.1) and PtABI1B (*Populus tremula*; CAM84257). Black box indicates the NLS-like nuclear localization sequence motif at the C-terminus; red lines indicate the amino acid residues involved in the interaction with ABA receptors (green lines indicate PYLs interaction); blue lines indicate the amino acid residues involved in the de-phosphorylation. The red dot, blue triangle and black triangle stand for the contact point with metal, phosphate and ABA, respectively.

Table S1 Primer sequences used in this study

Primer	Forward sequence (5'-3')	Reverse sequence (5'-3')
**CDS cloning**		
GhPP2C1-CDS	ATGGAGGAAATGACATTGTCTCTGC	TTAAGGTCTGCTCTTGAACTTTCTC
GhNAC83-CDS	ATGGATCAGAAGCTCATGAGATTG	CTAGCGACACCGAGCAATGTGC
**Virus-induced gene silencing (VIGS)**		
TRV1	TTACAGGTTATTTGGGCTAG	CCGGGTTCAATTCCTTATC
TRV2	TGGGAGATGATACGCTGTT	CCTAAAACTTCAGACACG
GhPP2C1/TRV2	GCTCTAGACAACGAACGAGAGAAGATGAGT	GGGGTACCCTCATACAACAACTACAGCCCC
GhNAC83/TRV2	CGCGGATCCCAATCGAATTGGTCGGAGGT	CCGCTCGAGCTCTCTCGCTAATGTCATTTG
GhIPT/TRV2	CGCGGATCCTATGGTAGGATCTACTCACTGA	CCGCTCGAGGCAAATAGGCTCATATATATTCTC
GhCYP735A/TRV2	CGCGGATCCGTCGGAGTACCCTTCAGCAA	CCGCTCGAGGGTCCAAGTTAGGGCAAGTC
**Qrt-PCR**		
GhACTIN-QRT	ACTGCAGAGCGGGAAATTGT	CCAATCAGGGATGGCTGGAA
GhPP2C1-QRT	CGAGATCGTGTCCAGGGATG	ACCCCACTGCTAATGACAGC
GhdUTPase-QRT	TTATGCCCGCATCGCTCCAA	GACAACTCCCACCGGTCCAC
GhNAC83-QRT	AGATTGCCACCTGGCTTCCG	CGACAGCGCCTTCCTCTTGA
GhLEA-QRT	TCCGTTATCGCCAAGTCCAA	CGGATCCATCTCGACAACCC
GhRD29B-QRT	GGGACGTCAGTGAAGGAGTT	TCTGATCCGTTGTCTCTTGC
GhIPT-QRT	GGACTCCGGCAAGTTCCTCA	GTTGAATTGCGTGGCGAGGT
GhCYP735A-QRT	CGACGAGGGAGCTTGTGGAT	CCTCCTCCCTTAGCCGCTTC
**Subcellular localization**		
GhNAC83/Pcambia1300-GFP	TCCCCCCGGGATGGATCAGAAGCTCATGAGATTG	GGGGTACCGCGACACCGAGCAATGTGC
**Transactivation domain analysis**		
GhNAC-FL/Pgbkt7	GGAATTCCATATGATGGATCAGAAGCTCATGAGA	TCCCCCCGGGCGACACCGAGCAATGTGC
GhNAC-NP/Pgbkt7	GGAATTCCATATGATGGATCAGAAGCTCATGAGA	TCCCCCCGGGCCTTGGTAGAAGACTAGAACCCT
GhNAC-CP/Pgbkt7	GGAATTCCATATGAAGCCTCCCTATGGGTCT	TCCCCCCGGGCGACACCGAGCAATGTGC
GhNAC-CT/Pgbkt7	GGAATTCCATATGGCAAAATACCATGATAATCT	TCCCCCCGGGCGACACCGAGCAATGTGC
**Promoter cloning and analysis**		
GhPP2C1-hitail-R1	CCAATCGACCTTGACATTGCAA	

	GAAC	
GhPP2C1-hitail-R2	CCACTGGATGATCTTGCCCCCAGCA	
GhPP2C1-hitail-R3	CTTGCCCCCAGCAGCCTCAATCCG	
GhPP2C1-Promoter-P0/Pcambia1391	CCCAAGCTTAGATTAAGAGTATGGAGTATAC	TCCCCCGGGCCCTTCCCCTCCTCAAGATA
GhPP2C1-Promoter-P1/Pcambia1391	CCCAAGCTTCTCTGACAGGGACGTTAAC	TCCCCCGGGCCCTTCCCCTCCTCAAGATA
GhPP2C1-Promoter-P2/Pcambia1391	CCCAAGCTTTTTCCGGTTGGTGGGGCCCA	TCCCCCGGGCCCTTCCCCTCCTCAAGATA
**Yeast one-hybrid**		
pDEST-GAD424	CTATTCGATGATGAAGATACCCC	CGTTTTAAAACCTAAGAGTCAC
GhPP2C1 pro/pDESTHISi-2	ggggacaactttgtatagaaaagttgccCTCTGACAGGGACGTTAACC	ggggactgcttttttgtacaaacttgcCGTTAGCTCTGGTGGGGCCC
GhNAC83/Pdestgad424	ggggacaagtttgtacaaaaaagcaggctccATGGATCAGAAGCTCATGAGATTG	ggggaccactttgtacaagaaagctgggttCTAGCGACACCGAGCAATGTGC
GhbZIP1/Pdestgad424	ggggacaagtttgtacaaaaaagcaggctccATGGACCAGAGGAAGAGGA	ggggaccactttgtacaagaaagctgggttTCAGAACTGCAACATATCGGCCG
GhWRKY40/Pdestgad424	ggggacaagtttgtacaaaaaagcaggctccATGGAGGAGGTGGAGGAGGC	ggggaccactttgtacaagaaagctgggttCTAGGTAGTGGCCGACTGGGTG
GhMYB1R1/Pdestgad424	ggggacaagtttgtacaaaaaagcaggctccATGACGAGAAGGTGCTCCCATTG	ggggaccactttgtacaagaaagctgggttTCATGTTTGGGCCACTTGGGGT
GhAPL-like/Pdestgad424	ggggacaagtttgtacaaaaaagcaggctccATGTTCTCTGGGTTGATTCAG	ggggaccactttgtacaagaaagctgggttTTAATTCAGTGCCTTCCGCTTC
GhDof1.8/Pdestgad424	ggggacaagtttgtacaaaaaagcaggctccATGGAGGAACTGTTGTCTTCT	ggggaccactttgtacaagaaagctgggttTTACTCTCCTCTTCTCCAGATC
GhbZIP60/Pdestgad424	ggggacaagtttgtacaaaaaagcaggctccATGGAAGAAGTAGCTCTATCTCAAG	ggggaccactttgtacaagaaagctgggttCTAAGCAACAAGAACAACACAAGG
GhBPC1/Pdestgad424	ggggacaagtttgtacaaaaaagcaggctccATGGATGATCATGGGGGATTAG	ggggaccactttgtacaagaaagctgggttCGAACAAGTTTGTGACTATCAGGTAA
GhIPT-promoter-T1/pDESTHISi-2	ggggacaactttgtatagaaaagttgccGTGAAAAAAATAATGTGGCACTGC	ggggactgcttttttgtacaaacttgcTACATGCAATATTTGTTGCACCAT
GhIPT-promoter-T2/pDESTHISi-2	ggggacaactttgtatagaaaagttgccTCGTATAGCTTTCCGACTTC	ggggactgcttttttgtacaaacttgcCTATGATTAGTTATACTGTTACAACATC
GhIPT-promoter-T3/pDESTHISi-2	ggggacaactttgtatagaaaagttgccTGGTATCAATACATATATGGATGCT	ggggactgcttttttgtacaaacttgcTATGTTCTCAGTGCATGGATTG

**Mutant promoter**		
GhPP2C1 pro/pDESTHISi-2F1	ggggacaactttgtatagaaaagttgccCTCTGACAGGGACGTTAACC	
GhPP2C1pro^{MUT}-R1	CggGATCTTCTCTTCTCCGTAGgGCcCCGGAAACCTTTCACCCATGG	
GhPP2C1pro^{MUT}-F2	TACGGAGAAGAGAAGATCccGACcTtTGCTAAATTGGGGTGGACACG	
GhPP2C1 pro/pDESTHISi-2F2	ggggactgcttttttgtacaaacttgcCGTTAGCTCTGGTGGGGCCC	
GhIPTpro^{MUT}-T2F1	ggggacaactttgtatagaaaagttgccTCcTATAGCTTTCCGACTTC	
GhIPTpro^{MUT}- T2R1	ATGCGgGAAGGTGGACTGTTTAGTTCT	
GhIPTpro^{MUT}- T2F2	AACTAAACAGTCCACCTTCcCGCATGAACTTGCCAACATA	
GhIPTpro^{MUT}- T2F2	ggggactgcttttttgtacaaacttgcCTATGATTAGTTATACTGTTACAACATC	
**Dual-luciferase**		
GhIPT-promoter/pGreenII LUC F/R	ctatagggcgaattgggtaccCCTTTTAATTTGTAGAGAGAGGAGTTTG	tgtttttggcgtcttccatggGGCCACTTAGTTAATTACAAGTGTG
GhIPTpro^{MUT}-T2R-1		GCTATAgGAGCTCGATGTCTGGTTGTCTGTAATTGGAATTAC
GhIPTpro^{MUT}-T2F1-1	ACAGACAACCAGACATCGAGCTCcTATAGCTTTCCGACTTC	
GhIPTpro^{MUT}- T2R1		ATGCGgGAAGGTGGACTGTTTAGTTCT
GhIPTpro^{MUT}- T2F2	AACTAAACAGTCCACCTTCcCGCATGAACTTGCCAACATA	
GhPP2C1p/pGreenII LUC F/R	ctatagggcgaattgggtaccAGATTAAGAGTATGGAGTATAC	tgtttttggcgtcttccatggCCCTTCCCCTCCTCAAGATA
GhPP2C1pro^{MUT}-R1		CggGATCTTCTCTTCTCCGTAGgGCcCCGGAAACCTTTCACCCATGG
GhPP2C1pro^{MUT}-F2	TACGGAGAAGAGAAGATCccGACcTtTGCTAAATTGGGGTGGACACG	
GhNAC83/pGreenII SK F/R	ggcggccgctctagaactagtATGGATCAGAAGCTCATGAGATTG	cagcgaattggtaccgggcccCTAGCGACACCGAGCAATGTGC
