# Supplementary material for: GhNAC83 inhibits corm dormancy release by regulating ABA signaling and cytokinin biosynthesis in Gladiolus hybridus
Source: J Exp Bot. 2018 Dec 5;70(4):1221–37. doi: 10.1093/jxb/ery428 (PMC6382327; doi:10.1093/jxb/ery428)
Supplement: Supplementary Table S2 [file ery428_suppl_supplementary_table_s2.pdf]

Table S2 Overlapped different expression genes in all stages

Gene ID	WD VS DD Fold	WD VS DD Log_2FC	ED VS WD Fold	ED VS WD Log_2FC	Annotation
GlaUn000047	249.43	1E-08	0.11	2.8E-05	-
GlaUn000344	0.37	0.00148	0.09	2.5E-12	-
GlaUn000487	15.19	0.00114	5.71	3.1E-05	PREDICTED: Phoenix dactylifera probable calcium-binding protein CML14 (LOC103715953), mRNA
GlaUn000610	28.11	2.9E-05	0.36	0.02636	PREDICTED: Elaeis guineensis uncharacterized LOC105039139 (LOC105039139), mRNA
GlaUn000620	2.15	0.00471	2.07	0.02032	PREDICTED: Brassica napus cellulose synthase-like protein D2 (LOC106387704), transcript variant X3, mRNA
GlaUn000661	5.45	0.02125	2.88	0.01675	PREDICTED: Vitis vinifera ethylene-responsive transcription factor 2 (LOC100254640), mRNA
GlaUn000832	33.29	3E-15	0.35	1E-05	-
GlaUn001010	0.22	1.6E-07	0.05	1.4E-05	-
GlaUn001151	0.31	0.01299	0.12	0.03667	Triticum aestivum chromosome 3B, genomic scaffold, cultivar Chinese Spring
GlaUn001284	0.35	0.0122	0.37	0.00948	-
GlaUn001305	5.48	0.00024	0.41	0.00435	-
GlaUn001526	0.41	0.02848	0.19	0.00298	-
GlaUn001607	16.36	0.00317	7.74	0.0272	-
GlaUn001981	0.38	9.8E-05	0.14	3.6E-07	-
GlaUn002034	6.67	0.04248	4.35	0.0001	-
GlaUn002092	0.04	0.04694	92.3	1.9E-10	-
GlaUn002174	15.7	0.01335	0.16	0.01413	-
GlaUn002297	0.47	0.03122	0.08	3.9E-09	-
GlaUn002424	0.36	0.00836	0.47	0.02663	-
GlaUn002649	0.26	0.0032	8.41	1.1E-06	-
GlaUn002680	33.33	0.04405	11.05	1E-06	PREDICTED: Elaeis guineensis transcription factor bHLH35 (LOC105050852), transcript variant X3, mRNA
GlaUn002751	0.34	0.00149	0.24	5.1E-05	-
GlaUn002805	14.27	1E-08	0.26	2.5E-05	-
GlaUn002878	2.12	0.00202	0.15	1.2E-16	-
GlaUn003102	15.48	8.5E-24	0.2	2.9E-14	-
GlaUn003155	0.32	0.01118	0.15	4.2E-07	-
GlaUn003482	3.43	0.00034	0.06	4.3E-17	-
GlaUn003483	2.75	0.00127	0.04	4.6E-25	-
GlaUn003573	0.42	0.01906	0.37	0.00096	PREDICTED: Elaeis guineensis transmembrane 9 superfamily member 3-like (LOC105053145), transcript variant X2, mRNA
GlaUn003584	21.42	0.00488	0.18	0.0164	-
GlaUn003678	29.92	4.3E-05	0.19	0.00277	-
GlaUn003821	21.94	0.00121	0.11	0.00137	-
GlaUn003867	2.44	0.00025	0.2	2.5E-12	-
GlaUn003908	0.36	0.02097	0.24	0.00092	-
GlaUn004050	0.31	0.01673	0.24	2.6E-07	-
GlaUn004170	0.3	0.02778	0.16	0.00329	-
GlaUn004231	2.07	0.00224	0.22	3.5E-12	-
GlaUn004299	0.29	0.01127	0.02	3.3E-05	-
GlaUn004310	6.21	0.01838	3.12	0.00014	Cocos nucifera serine-threonine kinase STK6 mRNA, complete cds
GlaUn004335	0.39	0.01275	0.37	0.02374	-
GlaUn004404	0.29	0.01325	9.43	5.3E-13	-
GlaUn004602	5.85	0.01371	2.12	0.02199	PREDICTED: Phoenix dactylifera arginine decarboxylase-like (LOC103697802), mRNA
GlaUn004884	0.3	0.03675	0.17	0.01097	PREDICTED: Nelumbo nucifera serine/arginine-rich splicing factor SR34A (LOC104593794), transcript variant X3, mRNA
GlaUn004903	28.75	3.9E-05	0.4	0.04497	-
GlaUn004948	6.56	1.4E-11	0.06	3.4E-22	-
GlaUn005054	0.36	0.03545	6.62	3.4E-11	-
GlaUn005068	17.98	9.4E-30	0.17	1.4E-17	-
GlaUn005131	0.4	0.01163	0.31	0.00013	-
GlaUn005233	7.85	3.9E-05	2.34	0.01634	-
GlaUn005676	0.37	0.00862	0.15	0.00032	-

GlaUn005862	20.12	6.7E-05	15.29	3.8E-06	PREDICTED: Ricinus communis cysteine-rich and transmembrane domain-containing protein A (LOC8264908),
GlaUn005863	18.47	0.00182	15.09	3.3E-06	PREDICTED: Ricinus communis cysteine-rich and transmembrane domain-containing protein A (LOC8264908),
GlaUn006476	7.26	0.00254	6.1	5.6E-05	-
GlaUn008009	6.18	1.2E-06	8.56	0.0024	PREDICTED: Phoenix dactylifera arabinogalactan peptide 3-like (LOC103712951), mRNA
GlaUn008387	0.26	0.00443	0.08	0.00318	-
GlaUn008592	5.53	4.5E-06	2.91	0.00079	-
GlaUn009869	2.32	7.4E-05	0.21	4.5E-15	-
GlaUn010205	0.3	0.00151	0.23	0.00783	-
GlaUn011242	0.39	0.01507	0.3	0.00036	Siraitia grosvenorii isopentenyl diphosphate isomerase mRNA, complete cds
GlaUn011270	0.08	0.02732	217.44	7.9E-44	Oryza sativa Indica Group cultivar RP Bio-226 chromosome 2 sequence
GlaUn011460	7.33	0.02259	3.64	2.7E-05	-
GlaUn011930	0.35	0.0002	0.21	1.3E-08	-
GlaUn012767	6.22	0.00014	4.25	0.00132	-
GlaUn012786	0.28	0.0431	0.12	7.2E-08	-
GlaUn013154	0.39	0.00042	0.41	1.6E-05	-
GlaUn013538	0.31	0.00022	0.24	4.4E-05	PREDICTED: Phoenix dactylifera isopentenyl-diphosphate Delta-isomerase I (LOC103705690), transcript variant X2,
GlaUn013683	191	0.02404	3	0.02753	-
GlaUn014194	0.24	0.03655	0.1	0.03997	-
GlaUn014204	11.15	0.01275	2.68	0.01327	-
GlaUn014255	0.41	0.02008	0.18	1.4E-06	-
GlaUn014495	0.49	0.01533	0.39	8.2E-07	Ipomopsis rubra ribulose-1,5-bisphosphate carboxylase/oxygenase large subunit (rbcL) gene, partial cds; chloroplast
GlaUn014989	0.19	0.01178	0.02	0.01628	-
GlaUn015261	4.72	0.00093	2.94	0.00065	-
GlaUn015334	0.43	0.04144	0.09	5.4E-08	-
GlaUn015525	0.29	0.03574	0.24	0.01655	-
GlaUn016004	2.15	0.00879	0.08	4.8E-17	-
GlaUn017111	456.33	0.02663	4.77	6.7E-07	PREDICTED: Brassica napus probable CCR4-associated factor 1 homolog 11 (LOC106371662), mRNA
GlaUn017158	38.93	3.5E-26	0.23	1E-09	-
GlaUn017488	0.4	0.02951	0.2	2.7E-07	PREDICTED: Phoenix dactylifera light-regulated protein-like (LOC103707811), mRNA
GlaUn017827	0.46	0.01868	0.22	2.1E-09	-
GlaUn018187	0.36	0.04005	11.23	3.2E-34	-
GlaUn018188	0.36	0.03478	11.25	2.8E-34	-
GlaUn018555	4.87	0.01853	10.45	4.2E-18	PREDICTED: Sesamum indicum peamaclein-like (LOC105159126), mRNA
GlaUn018753	0.3	0.0008	0.13	0.02187	Gladiolus grandiflorus polyubiquitin (UBQ) gene, promoter region and partial cds
GlaUn018933	9.45	6.2E-05	0.37	0.00402	-
GlaUn020755	5.77	1.1E-06	0.05	4.6E-14	-
GlaUn020756	5.49	6.9E-06	0.05	4.6E-13	-
GlaUn020822	9.21	0.00631	0.3	0.02145	PREDICTED: Musa acuminata subsp. malaccensis uncharacterized LOC103970718 (LOC103970718), mRNA
GlaUn021295	5.53	0.0142	2.55	0.02343	-
GlaUn021441	0.2	0.0003	31.92	3E-38	Ruta oreojasme clone RO62 microsatellite sequence
GlaUn021522	6.34	0.00354	4.31	0.00131	PREDICTED: Musa acuminata subsp. malaccensis UDP-glucose 6-dehydrogenase 4-like (LOC103968572), transcript variant X2, mRNA
GlaUn021893	0.37	0.00078	0.12	4.1E-12	PREDICTED: Elaeis guineensis phosphatidylinositol/phosphatidylcholine transfer protein SFH8-like (LOC105058769), transcript variant X3, misc_RNA
GlaUn021894	0.39	0.00798	0.24	1.3E-05	PREDICTED: Elaeis guineensis phosphatidylinositol/phosphatidylcholine transfer protein SFH8-like (LOC105058769), transcript variant X3, misc_RNA
GlaUn022034	4.07	0.04602	10.55	2.3E-34	PREDICTED: Musa acuminata subsp. malaccensis cytochrome c-like (LOC103983621), mRNA
GlaUn022103	0.3	0.0004	0.24	0.00026	-
GlaUn023070	0.18	0.02949	0.07	0.00437	-

GlaUn023163	2.68	0.01317	0.11	7.5E-09	-
GlaUn023645	0.33	0.00259	0.17	1.2E-05	-
GlaUn023646	0.29	3.1E-05	0.08	7.8E-11	-
GlaUn023752	0.39	9.1E-05	0.09	6.2E-16	-
GlaUn023844	0.49	0.03798	0.41	0.04184	PREDICTED: Elaeis guineensis thioredoxin-like 1-2, chloroplastic (LOC105057263), mRNA
GlaUn023849	0.29	0.00252	0.27	0.00559	-
GlaUn024725	4.26	0.00354	2.25	0.03532	-
GlaUn024856	2.41	0.00659	0.05	3E-45	-
GlaUn024899	15.52	0.01183	3.9	0.00305	PREDICTED: Elaeis guineensis U-box domain-containing protein 27-like (LOC105052049), mRNA
GlaUn024947	2.53	0.00337	2.69	0.00019	Triticum aestivum chromosome 3B, genomic scaffold, cultivar Chinese Spring
GlaUn025736	0.32	0.01942	0.28	0.0027	-
GlaUn027038	0.23	0.00142	0.2	0.01695	Morus notabilis hypothetical protein partial mRNA
GlaUn027070	3.58	0.01037	2.66	0.00375	-
GlaUn027071	12.33	0.04095	18.61	5.4E-44	PREDICTED: Phoenix dactylifera pyruvate decarboxylase 1 (LOC103695777), transcript variant X2, mRNA
GlaUn027072	13.29	0.01546	21.19	4.7E-46	Lycoris aurea mutant pyruvate decarboxylase mRNA, complete cds
GlaUn027210	7.01	0.01096	2.58	0.03259	-
GlaUn027282	10.96	6.4E-05	0.23	0.00068	-
GlaUn027689	0.23	0.03268	0.02	0.002	-
GlaUn027797	43.67	0.00625	0.2	0.03951	-
GlaUn027966	7.56	0.01481	5.6	0.00021	PREDICTED: Musa acuminata subsp. malaccensis protein UPSTREAM OF FLC-like (LOC103972317), mRNA
GlaUn028013	2.69	0.00014	3.96	5.5E-06	PREDICTED: Phoenix dactylifera histone H3.v1-like (LOC103717319), mRNA
GlaUn028094	0.47	0.02615	6.36	1.5E-09	PREDICTED: Elaeis guineensis bidirectional sugar transporter SWEET1a-like (LOC105037698), mRNA
GlaUn028102	6.01	0.00367	0.44	0.03084	PREDICTED: Elaeis guineensis probable calcium-binding protein CML10 (LOC105034665), mRNA
GlaUn028256	2.97	6.9E-05	0.14	5.5E-13	-
GlaUn028482	8.05	0.00057	4.57	8.8E-13	Oryza sativa Japonica Group DNA, chromosome 7, cultivar: Nipponbare, complete sequence
GlaUn028588	16.44	0.01415	4.47	1E-05	Phleum pratense DNA, SSR, microsatellite B01-F10
GlaUn028763	0.4	0.00016	0.49	9.3E-06	-
GlaUn028945	0.36	0.01158	0.42	0.01599	-
GlaUn029234	5.11	0.03742	5.88	4.5E-11	-
GlaUn029417	29.82	7.9E-18	0.19	1.6E-10	-
GlaUn029521	0.19	0.01325	201.16	3.3E-33	PREDICTED: Phoenix dactylifera uncharacterized LOC103715444 (LOC103715444), transcript variant X4, ncRNA
GlaUn029550	28.88	0.00048	0.17	0.0051	-
GlaUn029553	32.17	0.00023	0.16	0.00316	-
GlaUn030102	2.34	0.02792	3.49	5.6E-07	-
GlaUn030185	2	0.03302	0.19	3.6E-09	PREDICTED: Elaeis guineensis indole-3-acetic acid-amido synthetase GH3.17-like (LOC105051115), mRNA
GlaUn030352	0.34	6E-05	0.13	9.7E-07	PREDICTED: Elaeis guineensis phosphatidylinositol/phosphatidylcholine transfer protein SFH8-like (LOC105045972), transcript variant X4, mRNA
GlaUn030354	0.27	5.8E-05	0.13	5.3E-06	PREDICTED: Glycine max phosphatidylinositol/phosphatidylcholine transfer protein SFH8-
GlaUn030381	4.8	0.03277	3.72	0.01246	-
GlaUn030679	5.23	2.6E-16	2.74	3.5E-06	PREDICTED: Elaeis guineensis probable protein phosphatase 2C 32 (LOC105057113), mRNA
GlaUn030761	3.63	0.04395	2.26	0.03366	-
GlaUn031536	2.46	0.04102	0.02	3.6E-45	-
GlaUn031554	32.83	0.00893	3.13	0.01754	-
GlaUn031555	33.83	0.00194	2.99	0.02603	-
GlaUn031867	2.56	0.0489	275.3	0.00099	PREDICTED: Elaeis guineensis phosphoserine aminotransferase 2, chloroplastic-like (LOC105053256), mRNA
GlaUn031891	0.45	0.00146	0.12	1E-27	PREDICTED: Elaeis guineensis formin-A-like (LOC105056046), transcript variant X2, misc_RNA
GlaUn031892	0.46	0.00286	0.12	3.9E-28	PREDICTED: Elaeis guineensis formin-A-like (LOC105056046), transcript variant X2, misc_RNA
GlaUn032100	3.24	0.03277	3.66	1.2E-07	-

GlaUn032315	3.37	8.4E-07	0.07	8.6E-23	-
GlaUn032319	0.38	0.02577	10.38	6.2E-31	PREDICTED: Elaeis guineensis probable E3 ubiquitin-protein ligase XERICO (LOC105049948), mRNA
GlaUn032638	0.34	0.04787	0.1	0.00026	-
GlaUn032640	0.36	0.04959	0.09	0.0003	-
GlaUn032922	0.47	0.02741	0.43	0.0332	PREDICTED: Elaeis guineensis thioredoxin-like 1-2, chloroplastic (LOC105057263), mRNA
GlaUn033048	3.27	0.04706	0.02	2.1E-08	-
GlaUn033049	2.89	0.01508	0.03	9.3E-12	-
GlaUn033174	0.17	0.04901	239.07	5.8E-40	PREDICTED: Phoenix dactylifera uncharacterized LOC103715444 (LOC103715444), transcript variant X4, ncRNA
GlaUn033264	0.37	0.00092	0.35	0.00999	PREDICTED: Musa acuminata subsp. malaccensis abscisic stress-ripening protein 2-like (LOC103971257), mRNA
GlaUn033391	0.18	0.03207	0.23	0.03987	-
GlaUn033631	0.37	0.01994	0.23	0.00968	PREDICTED: Phoenix dactylifera RING-H2 finger protein ATL16-like (LOC103723035), mRNA
GlaUn033736	10.15	1.2E-07	0.43	0.00375	PREDICTED: Elaeis guineensis protein ASPARTIC PROTEASE IN GUARD CELL 1-like (LOC105040986), mRNA
GlaUn034087	7.63	0.02691	2.97	0.01422	PREDICTED: Elaeis guineensis uncharacterized LOC105049936 (LOC105049936), mRNA
GlaUn034176	3.34	0.03842	3.21	1.1E-06	-
GlaUn034177	3.6	0.00488	3.47	2.1E-08	-
GlaUn034333	18.32	0.00198	0.19	0.00905	-
GlaUn034334	19.1	0.00295	0.2	0.01336	-
GlaUn034518	49.69	3.2E-05	0.33	0.02106	PREDICTED: Elaeis guineensis putative lipoxygenase 5 (LOC105041807), mRNA
GlaUn034532	8.88	0.00259	5.58	0.00096	Vigna angularis var. angularis DNA, chromosome 2, almost complete sequence, cultivar: Shumari
GlaUn034787	0.31	0.04245	0.26	0.02789	-
GlaUn035099	3.75	0.0425	2.4	3.3E-05	PREDICTED: Elaeis guineensis uncharacterized LOC105053284 (LOC105053284), ncRNA
GlaUn035100	4.17	0.04159	2.99	4.2E-08	PREDICTED: Elaeis guineensis uncharacterized LOC105053291 (LOC105053291), mRNA
GlaUn035225	4.61	0.00128	2.07	0.01473	PREDICTED: Vitis vinifera ethylene-responsive transcription factor 2 (LOC100254640), mRNA
GlaUn035226	4.65	0.00232	2.04	0.01716	PREDICTED: Vitis vinifera ethylene-responsive transcription factor 2 (LOC100254640), mRNA
GlaUn035395	22.71	0.00567	0.27	0.04685	-
GlaUn035701	0.11	0.03208	0.03	0.02818	-
GlaUn035925	2.26	0.00887	2.06	0.01372	PREDICTED: Elaeis guineensis uncharacterized LOC105054518 (LOC105054518), mRNA
GlaUn035937	0.42	0.02477	0.28	0.00791	-
GlaUn036000	2.26	0.01539	2.9	4.9E-05	Dioscorea oppositifolia AT4G29520-like protein mRNA, partial cds
GlaUn036001	2.24	0.00931	3.2	2.4E-06	PREDICTED: Elaeis guineensis uncharacterized LOC105042827 (LOC105042827), mRNA
GlaUn036193	6.17	0.01432	3.05	0.00195	PREDICTED: Nelumbo nucifera inositol-tetrakisphosphate 1-kinase 1-like (LOC104609603), transcript variant X2, misc_RNA
GlaUn036437	5.16	0.01962	4.85	0.00872	-
GlaUn036576	8.49	0.00613	2.67	0.01338	-
GlaUn036698	23.19	3E-15	0.26	2.5E-07	-
GlaUn036699	24.04	9.4E-15	0.27	8.1E-07	-
GlaUn036747	13.11	0.00049	5.19	1.1E-05	-
GlaUn036908	0.41	0.00069	0.26	3.8E-11	PREDICTED: Musa acuminata subsp. malaccensis 21 kDa protein-like (LOC103975492), mRNA
GlaUn037087	3.24	0.00089	0.14	6.3E-08	Panicum virgatum clone E06_PVCAG-7 microsatellite sequence
GlaUn037242	6.88	0.04297	3.04	0.00609	-
GlaUn037374	2.4	0.04731	2.34	0.00325	PREDICTED: Phoenix dactylifera cytochrome b5 (LOC103722253), mRNA
GlaUn037379	3.26	0.0003	2.1	0.00083	-
GlaUn037412	3.63	0.006	5.77	7.8E-13	PREDICTED: Elaeis guineensis ethylene-responsive transcription factor ERF061-like (LOC105047575), mRNA
GlaUn037694	5.31	1.7E-08	0.46	0.00082	-
GlaUn037793	2.71	0.00728	3.5	0.02322	-
GlaUn037794	2.56	0.00688	3.24	0.03891	-
GlaUn037812	3.17	0.00122	0.06	4.9E-13	-
GlaUn037820	6.43	9.3E-05	2.54	0.00356	-

GlaUn037821	6.69	0.00026	2.51	0.00536	-
GlaUn037948	37.75	0.03694	24.48	2.3E-20	PREDICTED: Phoenix dactylifera S-type anion channel SLAH1-like (LOC103703420), mRNA
GlaUn038261	0.29	0.00427	0.19	0.0253	-
GlaUn038331	52.33	0.03374	21.15	1.1E-21	-
GlaUn038442	0.35	0.01744	0.17	0.00679	-
GlaUn038832	8.09	0.01265	5.44	6.3E-05	-
GlaUn038892	2.4	0.00114	0.14	8.7E-14	-
GlaUn039104	46.42	7.4E-07	0.4	0.03263	-
GlaUn039470	2.09	0.03705	6.38	4.7E-06	Gossypium hirsutum clone NBRI_GE66211 microsatellite sequence
GlaUn039472	2.05	0.03287	6.22	1.7E-05	Gossypium hirsutum clone NBRI_GE66211 microsatellite sequence
GlaUn039536	24.32	0.00204	0.29	0.04095	PREDICTED: Phoenix dactylifera phospholipase A1-Ibeta2, chloroplastic (LOC103713597), mRNA
GlaUn039604	2.12	0.02858	0.13	3E-10	-
GlaUn039605	2.18	0.0256	0.13	4.9E-10	-
GlaUn039654	55.88	0.00634	3.83	0.00045	-
GlaUn039655	56.6	0.0202	3.77	0.00046	-
GlaUn039872	4.83	0.02427	2.54	0.01742	PREDICTED: Musa acuminata subsp. malaccensis TGACG-sequence-specific DNA-binding protein TGA-1A-like (LOC103986471), mRNA
GlaUn039918	0.31	0.00352	20.99	6.2E-37	PREDICTED: Amborella trichopoda thaumatin-like protein 1b (LOC18441626), mRNA
GlaUn039919	0.38	0.02488	16.84	3E-33	PREDICTED: Amborella trichopoda thaumatin-like protein 1b (LOC18441626), mRNA
GlaUn040335	122.2	1.1E-07	0.23	0.00059	-
GlaUn040336	115.8	6.1E-07	0.24	0.00152	-
GlaUn040350	11.3	0.00212	0.35	0.03374	-
GlaUn040380	25.54	5.3E-05	0.33	0.01773	-
GlaUn040537	4.47	0.01489	5.61	2.6E-09	PREDICTED: Phoenix dactylifera 3-oxoacyl-[acyl-carrier-protein] reductase, chloroplastic-like (LOC103720253), mRNA
GlaUn040825	19.39	0.00481	0.26	0.03662	PREDICTED: Elaeis guineensis serine acetyltransferase 1, chloroplastic (LOC105032793), mRNA
GlaUn041014	2.92	0.01135	3.33	8.6E-05	Toxicodendron radicans subsp. hispidum microsatellite DNA locus M56
GlaUn041282	3.33	0.01943	3.58	0.00428	PREDICTED: Musa acuminata subsp. malaccensis UPF0496 protein At3g19330 (LOC103990869), mRNA
GlaUn041304	2.37	0.00038	2.4	0.04667	-
GlaUn041326	3.67	0.03157	3.32	8.5E-07	-
GlaUn041403	22.2	0.03752	2.63	0.00743	-
GlaUn041551	0.49	0.01387	0.33	0.00432	PREDICTED: Phoenix dactylifera repressor of RNA polymerase III transcription MAF1 homolog (LOC103709694), transcript variant X2, mRNA
GlaUn041553	0.38	0.00056	0.12	0.00049	-
GlaUn041556	0.34	0.00172	0.16	0.00038	PREDICTED: Phoenix dactylifera repressor of RNA polymerase III transcription MAF1 homolog (LOC103709694), transcript variant X1, mRNA
GlaUn041746	8.9	0.01806	2.24	0.04588	-
GlaUn041843	0.26	0.01268	24.69	9.7E-13	PREDICTED: Eucalyptus grandis thaumatin-like protein 1 (LOC104422763), mRNA
GlaUn041873	0.21	0.01098	0.05	0.03717	-
GlaUn042115	11.05	0.04148	4.2	0.00608	PREDICTED: Phoenix dactylifera U-box domain-containing protein 25-like (LOC103703189), mRNA
GlaUn042179	3.59	0.00755	3.23	0.02434	-
GlaUn042181	3.57	0.00713	2.59	0.02519	-
GlaUn042182	3.91	0.00443	2.08	0.0478	-
GlaUn042246	7.3	6E-06	2.56	0.00036	-
GlaUn042557	8.4	0.00845	2.14	0.00613	PREDICTED: Fragaria vesca subsp. vesca uncharacterized LOC101295283 (LOC101295283), ncRNA
GlaUn042973	2.79	0.04749	3.26	1.6E-08	PREDICTED: Elaeis guineensis protein YLS9-like (LOC105035820), mRNA
GlaUn043327	21.05	4.7E-09	0.46	0.01418	-
GlaUn043353	0.3	0.01636	0.04	0.00066	-
GlaUn043417	2.85	0.00532	4.91	0.00081	PREDICTED: Elaeis guineensis uncharacterized LOC105053895 (LOC105053895), mRNA
GlaUn043440	4.84	0.01868	2.51	0.029	-

GlaUn043675	2.15	0.00051	0.16	4.3E-24	PREDICTED: Phoenix dactylifera transcription factor PCF2-like (LOC103696613), mRNA
GlaUn043804	0.32	0.03272	0.31	0.02408	-
GlaUn044306	11.71	0.01687	2.75	8.5E-05	Hyacinthus orientalis pollen-specific protein mRNA, partial cds
GlaUn044344	62.73	0.00049	0.26	0.04206	-
GlaUn044354	2.33	0.00389	0.2	1.2E-07	-
GlaUn044410	16.75	0.00017	0.31	0.01279	-
GlaUn044567	0.37	0.03101	0.12	4E-06	-
GlaUn044603	4.78	0.00053	5.02	8.8E-10	PREDICTED: Elaeis guineensis auxin-induced protein X15-like (LOC105036306), mRNA
GlaUn044604	6.05	2.8E-05	5.53	8.7E-12	PREDICTED: Elaeis guineensis auxin-induced protein X15-like (LOC105036306), mRNA
GlaUn044652	0.17	0.01211	13.27	4.2E-08	PREDICTED: Phoenix dactylifera uncharacterized LOC103696034 (LOC103696034), mRNA
GlaUn044653	0.22	0.03667	10.43	1.8E-10	PREDICTED: Phoenix dactylifera uncharacterized LOC103696034 (LOC103696034), mRNA
GlaUn044682	0.23	0.02047	0.21	0.03282	-
GlaUn044782	31.08	0.00017	0.22	0.00779	PREDICTED: Phoenix dactylifera uncharacterized LOC103722767 (LOC103722767), mRNA
GlaUn044817	12.32	0.00505	3.12	0.00789	-
GlaUn044893	5.09	2.1E-10	0.49	0.00075	Androcymbium psammophilium microsatellite DNA locus ANDth-11
GlaUn044907	3.07	0.00097	0.09	6.7E-12	-
GlaUn044909	2.56	0.00189	0.1	3.7E-14	-
GlaUn044941	0.46	0.03969	0.38	0.00369	PREDICTED: Vitis vinifera uncharacterized LOC104881172 (LOC104881172), ncRNA
GlaUn045097	0.34	0.0058	0.48	0.03655	-
GlaUn045411	2.92	0.00014	6.7	6.5E-17	-
GlaUn045583	0.3	0.00886	6.99	8.1E-11	-
GlaUn045650	2.13	0.00015	0.13	2E-24	-
GlaUn045652	2.2	5.3E-05	0.12	1.5E-27	-
GlaUn045653	2.1	0.0105	0.22	2E-09	-
GlaUn045826	46.95	5.9E-09	0.24	0.00032	-
GlaUn045828	0.33	1.5E-05	0.16	0.0003	-
GlaUn045968	6.29	3.2E-06	2.63	0.00339	-
GlaUn045969	5.47	1.5E-06	2.24	0.01797	-
GlaUn046209	2.04	0.00277	2.09	0.0038	-
GlaUn046242	0.33	0.00294	0.13	0.00517	-
GlaUn046532	0.47	0.00973	0.34	8.1E-08	PREDICTED: Elaeis guineensis uncharacterized LOC105060016 (LOC105060016), mRNA
GlaUn046533	0.45	0.0058	0.39	1.9E-06	PREDICTED: Elaeis guineensis uncharacterized LOC105060016 (LOC105060016), mRNA
GlaUn046534	0.45	0.00434	0.43	1.5E-05	PREDICTED: Elaeis guineensis uncharacterized LOC105060016 (LOC105060016), mRNA
GlaUn046806	0.27	0.04088	56.3	1.8E-18	-
GlaUn046807	0.32	1.6E-06	37.3	2.1E-22	Lilium longiflorum pollen caleosin mRNA, complete cds
GlaUn046808	0.45	0.00574	16.72	1.6E-15	Lilium longiflorum pollen caleosin mRNA, complete cds
GlaUn046809	0.44	0.00405	16.24	2.7E-15	Lilium longiflorum pollen caleosin mRNA, complete cds
GlaUn046824	0.1	0.00919	25.21	3.4E-10	-
GlaUn046990	7.85	0.00017	3.37	0.00664	PREDICTED: Elaeis guineensis uncharacterized LOC105033200 (LOC105033200), mRNA
GlaUn047267	56.76	1.2E-11	0.34	0.00135	-
GlaUn047640	0.49	0.00627	0.22	8.1E-19	PREDICTED: Elaeis guineensis diacylglycerol O-acyltransferase 2 (LOC105052865), mRNA
GlaUn047641	0.47	0.00343	0.22	7.2E-19	PREDICTED: Elaeis guineensis diacylglycerol O-acyltransferase 2 (LOC105052865), mRNA
GlaUn047708	0.36	0.01278	0.14	1.1E-05	-
GlaUn048168	0.2	0.0058	0.2	0.03379	-
GlaUn048186	6.83	0.01188	2.39	0.00069	Phyllostachys edulis cDNA clone: bphyem107g20, full insert sequence
GlaUn048187	6.48	0.00598	2.31	0.00108	Phyllostachys edulis cDNA clone: bphyem107g20, full insert sequence
GlaUn048416	0.34	0.00884	0.08	1E-12	-
GlaUn048417	0.35	0.01008	0.08	3.9E-12	-
GlaUn048459	7.44	3.2E-22	0.47	1.5E-05	PREDICTED: Pyrus x bretschneideri F-box only protein 13-like (LOC103949665), mRNA

GlaUn048491	13.78	0.00627	5.58	0.02594	PREDICTED: Musa acuminata subsp. malaccensis pathogenesis-related genes transcriptional activator PTI5-like (LOC103989196), mRNA
GlaUn048765	2.26	3.6E-05	0.19	1E-20	-
GlaUn048824	6.37	3.4E-13	4.04	1.5E-10	-
GlaUn049086	5.68	0.01063	0.41	0.03371	Elaeis guineensis BI1-like protein (LOC105046588), mRNA
GlaUn049088	5.63	0.00424	0.41	0.02015	PREDICTED: Phoenix dactylifera BI1-like protein (LOC103704508), mRNA
GlaUn049122	7.53	1.2E-11	0.03	8.4E-26	-
GlaUn049172	12.17	1.3E-15	0.02	2.8E-24	-
GlaUn049848	0.43	0.02265	0.22	2.4E-06	Theobroma cacao SPFH/Band 7/PHB domain-containing membrane-associated protein family isoform 1 (TCM_018363) mRNA, complete cds
GlaUn050050	0.39	2.3E-05	0.38	4.2E-09	PREDICTED: Elaeis guineensis uncharacterized LOC105042973 (LOC105042973), transcript variant X3, mRNA
GlaUn050051	0.38	0.00015	0.4	8.1E-06	-
GlaUn050052	0.38	1.7E-05	0.37	2.2E-09	PREDICTED: Elaeis guineensis uncharacterized LOC105042973 (LOC105042973), transcript variant X3, mRNA
GlaUn050053	0.38	2E-05	0.37	1.9E-09	PREDICTED: Elaeis guineensis uncharacterized LOC105042973 (LOC105042973), transcript variant X3, mRNA
GlaUn050054	0.36	5E-06	0.36	2.1E-09	PREDICTED: Phoenix dactylifera uncharacterized LOC103721313 (LOC103721313), transcript variant X4, mRNA
GlaUn050189	6.83	0.01187	3.35	0.00014	-
GlaUn050202	5.86	0.00267	4	5.4E-12	PREDICTED: Phoenix dactylifera uncharacterized LOC103707423 (LOC103707423), mRNA
GlaUn050297	0.28	0.00155	17.84	1.8E-35	-
GlaUn050317	2.14	0.03916	2.6	0.0003	PREDICTED: Musa acuminata subsp. malaccensis probable WRKY transcription factor 71 (LOC103988756), mRNA
GlaUn050611	0.48	0.04994	0.38	0.00015	PREDICTED: Phoenix dactylifera uncharacterized LOC103720866 (LOC103720866), mRNA
GlaUn050652	2.76	9.7E-05	4.29	2.8E-12	PREDICTED: Phoenix dactylifera auxin response factor 19-like (LOC103715543), mRNA
GlaUn050861	2.66	0.00105	2.08	0.01243	PREDICTED: Elaeis guineensis uncharacterized LOC105054706 (LOC105054706), mRNA
GlaUn051298	0.17	0.04401	37.38	1.5E-10	PREDICTED: Phoenix dactylifera mannan endo-1,4-beta-mannosidase 1-like (LOC103712710), mRNA
GlaUn051540	2.5	0.0137	0.15	7E-08	-
GlaUn051541	3.12	0.00032	0.15	8.6E-10	PREDICTED: Populus euphratica protein REVEILLE 1-like (LOC105133175), transcript variant X2, mRNA
GlaUn051542	3.23	0.00015	0.15	2.6E-10	PREDICTED: Populus euphratica protein REVEILLE 1-like (LOC105133175), transcript variant X2, mRNA
GlaUn052029	59.48	1E-10	0.22	4.5E-05	-
GlaUn052055	30.83	0.01369	4.66	0.00012	PREDICTED: Musa acuminata subsp. malaccensis dnaJ homolog subfamily B member 14 (LOC103989384), mRNA
GlaUn052310	7.3	8.2E-05	26.44	7.8E-12	-
GlaUn052444	3.08	0.00776	3.72	2.5E-06	Oryza sativa Japonica Group DNA, chromosome 10, cultivar: Nipponbare, complete sequence
GlaUn052487	0.32	0.00164	0.13	4.6E-06	-
GlaUn052488	0.21	0.03773	0.06	0.04642	-
GlaUn052538	2.23	0.04025	10.58	6.9E-06	-
GlaUn052708	0.42	0.03366	0.23	3.1E-05	-
GlaUn052869	4.05	3.9E-11	2.28	0.00045	-
GlaUn052871	6.32	0.03508	5.25	4.3E-07	-
GlaUn052884	0.4	0.00353	0.12	3.9E-11	PREDICTED: Phoenix dactylifera glycerophosphodiester phosphodiesterase gde1-like (LOC103708094), transcript variant X2, mRNA
GlaUn053110	2.88	5.1E-05	3.82	0.03722	Saccharum hybrid cultivar BAC clone ShCIR9O20, selfed from cultivar R570
GlaUn053114	2.81	5.1E-05	3.82	0.03918	Saccharum hybrid cultivar BAC clone ShCIR9O20, selfed from cultivar R570
GlaUn053115	2.43	0.00562	3.41	0.04332	-
GlaUn053118	2.88	6.6E-05	3.81	0.04094	Saccharum hybrid cultivar BAC clone ShCIR9O20, selfed from cultivar R570
GlaUn053120	2.16	0.03867	3.87	0.0175	Saccharum hybrid cultivar BAC clone ShCIR9O20, selfed from cultivar R570
GlaUn053124	2.8	0.00404	3.95	0.04658	-
GlaUn053387	0.37	0.03323	0.22	0.0008	-
GlaUn053493	0.07	0.00035	224.77	3.1E-33	-

GlaUn053498	12.33	0.04649	2.43	0.0332	PREDICTED: Elaeis guineensis probable trehalose-phosphate phosphatase F (LOC105054029), transcript variant X4, mRNA
GlaUn054045	4.7	0.0073	4.66	2.5E-07	-
GlaUn054072	0.44	0.03361	0.01	4.6E-13	-
GlaUn054078	0.31	0.00887	0.01	9.2E-08	-
GlaUn054140	0.37	0.04501	0.25	2.1E-05	-
GlaUn054310	0.07	0.04901	275.3	2.5E-16	-
GlaUn054372	3.5	0.02065	11.47	0.02904	PREDICTED: Elaeis guineensis uncharacterized LOC105040143 (LOC105040143), transcript variant X2,
GlaUn054487	2.98	0.00822	0.06	3.8E-12	-
GlaUn054509	0.42	0.0028	11.89	8.7E-06	PREDICTED: Musa acuminata subsp. malaccensis naringenin,2-oxoglutarate 3-dioxygenase-like (LOC103993175),
GlaUn054871	2.51	0.04634	3.42	4.5E-06	PREDICTED: Elaeis guineensis bidirectional sugar transporter SWEET16-like (LOC105045177), mRNA
GlaUn054883	0.34	0.00153	0.21	0.0007	-
GlaUn054996	0.41	0.03992	0.13	9.9E-05	-
GlaUn055110	0.42	0.03906	0.42	0.00904	-
GlaUn055219	2.02	0.00022	0.13	1.5E-26	-
GlaUn055251	0.49	0.00736	0.07	3.1E-12	PREDICTED: Citrus sinensis uncharacterized LOC107177752 (LOC107177752), mRNA
GlaUn055252	0.27	6.1E-06	0.07	2E-10	-
GlaUn055253	0.42	0.00028	0.08	3.7E-14	-
GlaUn055492	4.08	0.04367	3.48	2.1E-06	PREDICTED: Elaeis guineensis chorismate mutase 2-like (LOC105034692), mRNA
GlaUn055881	28.9	1.7E-05	2.34	0.01881	-
GlaUn056406	2.02	0.01438	2.66	0.00031	PREDICTED: Elaeis guineensis scopoletin glucosyltransferase-like (LOC105047155), mRNA
GlaUn056436	0.24	0.00026	0.11	0.00648	-
GlaUn056437	0.21	0.00208	0.09	0.01873	-
GlaUn056478	0.37	0.00543	0.21	1.6E-05	-
GlaUn056648	2.24	0.03745	0.14	1.3E-08	-
GlaUn056894	0.31	0.00226	0.23	0.00066	-
GlaUn056895	0.32	0.00408	0.24	0.00078	-
GlaUn056977	2.18	0.02413	3.36	2.4E-06	-
GlaUn057026	0.38	0.02006	0.31	0.0017	PREDICTED: Phoenix dactylifera phototropin-2 (LOC103711883), mRNA
GlaUn057042	4.68	9.2E-12	2.9	0.03061	Oryza sativa Japonica Group DNA, chromosome 3, cultivar: Nipponbare, complete sequence
GlaUn057189	0.08	0.00193	57.4	1.7E-22	-
GlaUn057212	0.46	0.00366	0.26	3.1E-07	Ornithogalum longebracteatum NAC domain-containing protein 29 isoform 1 (NAC-1) mRNA, complete cds
GlaUn057597	2.62	0.00521	4.56	5.4E-15	PREDICTED: Phoenix dactylifera PRA1 family protein B4 (LOC103714372), mRNA
GlaUn057790	0.43	0.03873	12.98	1.4E-29	PREDICTED: Elaeis guineensis protein REVERSION-TO-ETHYLENE SENSITIVITY1 (LOC105059793), transcript variant
GlaUn057826	0.29	0.03014	0.18	0.02341	-
GlaUn057943	0.44	0.00589	0.1	5.5E-08	-
GlaUn057944	0.45	0.00582	0.09	3.7E-10	-
GlaUn057945	0.41	0.001	0.08	3.5E-08	-
GlaUn058057	49.9	5E-13	0.31	0.00023	-
GlaUn058058	37.15	1E-08	0.28	0.00097	-
GlaUn058133	0.26	0.01708	0.1	0.0021	-
GlaUn058348	0.45	0.03185	0.18	4.1E-08	-
GlaUn058521	3.09	0.00717	3.53	1.5E-10	PREDICTED: Musa acuminata subsp. malaccensis protein UPSTREAM OF FLC-like (LOC103972317), mRNA
GlaUn058522	3.27	0.02349	4.13	5.2E-13	PREDICTED: Elaeis guineensis protein UPSTREAM OF FLC-like (LOC105056990), mRNA
GlaUn058523	3.16	0.00852	3.58	9E-11	PREDICTED: Elaeis guineensis protein UPSTREAM OF FLC-like (LOC105032834), transcript variant X2, mRNA
GlaUn058524	3.18	0.00894	3.63	5.2E-11	PREDICTED: Elaeis guineensis protein UPSTREAM OF FLC-like (LOC105056990), mRNA
GlaUn058525	3.07	0.00767	3.53	1.5E-10	PREDICTED: Musa acuminata subsp. malaccensis protein UPSTREAM OF FLC-like (LOC103972317), mRNA
GlaUn058587	3.59	0.0006	5.85	2.7E-10	PREDICTED: Camelina sativa transcription factor DIVARICATA-like (LOC104764773), mRNA

GlaUn058593	3.74	0.0016	5.32	5E-12	PREDICTED: Camelina sativa transcription factor DIVARICATA-like (LOC104764773), mRNA
GlaUn058755	2.1	0.00196	3.6	2.3E-10	PREDICTED: Phoenix dactylifera double-stranded RNA-binding protein 2-like (LOC103713992), mRNA
GlaUn059241	2.45	0.01153	12.93	1.2E-09	PREDICTED: Elaeis guineensis mitochondrial chaperone BCS1-like (LOC105047854), mRNA
GlaUn059242	4.09	1.5E-06	15.2	1.7E-09	PREDICTED: Elaeis guineensis mitochondrial chaperone BCS1-like (LOC105047854), mRNA
GlaUn059243	3.03	0.00153	4.44	5.2E-05	PREDICTED: Elaeis guineensis mitochondrial chaperone BCS1-like (LOC105047854), mRNA
GlaUn059266	2.82	0.01116	38.68	2.6E-24	PREDICTED: Elaeis guineensis pectinesterase-like (LOC105053029), mRNA
GlaUn059267	2.91	0.01439	39.39	2.1E-24	PREDICTED: Elaeis guineensis pectinesterase-like (LOC105053029), mRNA
GlaUn059297	2.46	0.04592	6.38	7.8E-15	PREDICTED: Nelumbo nucifera ras-related protein RABA6b (LOC104609955), mRNA
GlaUn059448	0.3	0.01237	0.22	0.00348	-
GlaUn059500	0.28	0.03404	0.04	2.1E-05	-
GlaUn059569	15.64	0.02452	3.02	0.00025	PREDICTED: Elaeis guineensis brassinosteroid-regulated protein BRU1-like (LOC105049193), mRNA
GlaUn059570	13.74	0.03834	3.47	3.6E-06	PREDICTED: Nelumbo nucifera probable xyloglucan endotransglucosylase/hydrolase protein 23 (LOC104586223), mRNA
GlaUn059606	2.47	0.03111	2.27	0.04506	PREDICTED: Elaeis guineensis protein IQ-DOMAIN 1-like (LOC105044647), transcript variant X3, mRNA
GlaUn059694	0.38	0.01846	0.15	0.01116	-
GlaUn059725	21.28	0.0044	0.15	0.00861	-
GlaUn059748	2.08	0.00156	0.2	1.8E-12	PREDICTED: Phoenix dactylifera magnesium transporter MRS2-5-like (LOC103718206), transcript variant X2, mRNA
GlaUn059752	2.15	0.00127	0.19	1.8E-11	PREDICTED: Phoenix dactylifera magnesium transporter MRS2-5-like (LOC103718206), transcript variant X2, mRNA
GlaUn060055	16.2	0.02726	4.31	0.0352	-
GlaUn060056	22.12	0.00413	3.07	0.02676	-
GlaUn060667	2	0.00449	4.41	1.7E-11	PREDICTED: Phoenix dactylifera F-box/LRR-repeat protein 3 (LOC103719773), mRNA
GlaUn060967	13.19	6.6E-05	0.32	0.00641	PREDICTED: Phoenix dactylifera probable protein phosphatase 2C 4 (LOC103716499), mRNA
GlaUn061060	5.41	0.0134	3.6	3.9E-08	Triticum aestivum TaABA8'OH1-D mRNA for ABA 8' hydroxylase, complete cds
GlaUn061455	10.47	0.04754	0.21	0.0394	-
GlaUn061472	9.55	0.03238	3.33	0.00031	Gladiolus hybrid cultivar allene oxide synthase (AOS) mRNA, complete cds
GlaUn061683	11.41	1.8E-07	0.45	0.01133	-
GlaUn061690	15.82	1.3E-06	0.26	0.00035	-
GlaUn061771	12.01	0.00021	0.4	0.02886	-
GlaUn061797	0.33	0.02838	0.25	3.9E-05	-
GlaUn061843	7.22	0.00132	0.34	0.01068	-
GlaUn062139	0.19	9.2E-08	0.14	0.00028	Medicago truncatula chromosome 5 clone mth2-124p21, COMPLETE SEQUENCE
GlaUn062140	0.26	0.00123	0.33	0.03108	Medicago truncatula chromosome 5 clone mth2-124p21, COMPLETE SEQUENCE
GlaUn062578	0.48	0.00489	0.37	3.8E-05	PREDICTED: Elaeis guineensis beta-amylase (LOC105052391), mRNA
GlaUn062629	39.3	0.00022	0.25	0.02082	-
GlaUn062888	13.04	0.00243	3.39	0.00074	PREDICTED: Elaeis guineensis uncharacterized LOC105058510 (LOC105058510), mRNA
GlaUn062995	0.28	0.0001	0.25	0.00016	-
GlaUn062997	3.37	0.00024	6.32	3.9E-10	PREDICTED: Glycine max enoyl-CoA delta isomerase 1, peroxisomal (LOC100786464), mRNA
GlaUn063078	3.42	0.03574	3.46	0.00039	-
GlaUn063199	11.97	0.00753	3.58	0.02375	PREDICTED: Elaeis guineensis elicitor-responsive protein 1 (LOC105052228), transcript variant X2, mRNA
GlaUn063257	8.37	8.8E-07	2.83	9.3E-05	-
GlaUn063258	7.71	2.8E-05	2.81	0.00011	-
GlaUn063467	5.46	0.01182	6.91	4.1E-05	PREDICTED: Elaeis guineensis uncharacterized LOC105044597 (LOC105044597), mRNA
GlaUn063468	5.37	0.01182	6.82	5E-05	PREDICTED: Elaeis guineensis uncharacterized LOC105044597 (LOC105044597), mRNA
GlaUn063843	101.11	0.00024	0.19	0.00915	-

GlaUn063958	0.29	0.00802	0.3	0.0257	-
GlaUn063991	0.28	5.6E-06	7.4	5.6E-11	-
GlaUn063992	0.27	2.9E-06	7.85	7.4E-11	-
GlaUn064033	8.59	1.7E-17	0.45	7.4E-05	-
GlaUn064034	7.22	5.7E-15	0.48	2.1E-05	PREDICTED: Elaeis guineensis probable carboxylesterase 15 (LOC105034451), mRNA
GlaUn064117	0.34	1E-05	0.04	1.2E-27	-
GlaUn064154	2.07	7.4E-05	0.16	1.9E-21	-
GlaUn064156	2.16	0.00024	0.16	1E-19	-
GlaUn064183	15.33	0.04968	5.8	1.3E-10	PREDICTED: Elaeis guineensis myb-related protein Myb4-like (LOC105033428), mRNA
GlaUn064303	0.45	0.00443	0.19	1.4E-07	PREDICTED: Elaeis guineensis calcineurin B-like protein 3 (LOC105049942), transcript variant X4, mRNA
GlaUn064524	10.48	0.03673	5.02	9.1E-05	PREDICTED: Beta vulgaris subsp. vulgaris probable WRKY transcription factor 40 (LOC104884530), mRNA
GlaUn064635	5.28	0.00027	2.59	0.00381	-
GlaUn064673	0.47	0.0256	0.17	3.5E-11	PREDICTED: Elaeis guineensis uncharacterized LOC105045318 (LOC105045318), transcript variant X2, mRNA
GlaUn065097	22.71	0.04492	3.45	0.0121	PREDICTED: Musa acuminata subsp. malaccensis leucine-rich repeat receptor-like serine/threonine/tyrosine-protein kinase SOBIR1 (LOC103996472), mRNA
GlaUn065166	0.37	0.0157	0.3	0.00227	-
GlaUn065175	6.16	0.00077	0.4	0.00669	-
GlaUn065190	0.24	0.00097	0.23	0.00104	-
GlaUn065236	2.26	0.00706	2.18	0.00198	PREDICTED: Tarenaya hassleriana E3 ubiquitin-protein ligase ATL9-like (LOC104812700), mRNA
GlaUn065480	0.38	0.00025	0.4	0.00046	PREDICTED: Musa acuminata subsp. malaccensis zinc finger AN1 domain-containing stress-associated protein 15-like (LOC103979377), mRNA
GlaUn065539	0.17	0.0013	0.04	0.0077	-
GlaUn065551	4.91	2.6E-09	0.47	0.00042	-
GlaUn065755	0.38	0.00949	31.07	5.8E-61	PREDICTED: Musa acuminata subsp. malaccensis zinc finger protein 6-like (LOC103979231), mRNA
GlaUn065966	0.37	0.00024	0.29	3.6E-09	PREDICTED: Musa acuminata subsp. malaccensis organic cation/carnitine transporter 2-like (LOC103978707), mRNA
GlaUn066086	2.42	0.00548	2.44	0.00012	PREDICTED: Elaeis guineensis uncharacterized LOC105059075 (LOC105059075), mRNA
GlaUn067041	0.38	2.9E-05	5.71	1.2E-18	PREDICTED: Musa acuminata subsp. malaccensis pectinesterase-like (LOC103982084), mRNA
GlaUn067433	0.08	9.2E-05	4.9	0.02351	-
GlaUn067434	0.07	2.2E-05	5.24	0.01801	-
GlaUn067688	8.89	6.2E-17	0.46	0.00015	-
GlaUn067862	12.55	4.9E-09	0.24	4E-06	PREDICTED: Phoenix dactylifera uncharacterized LOC103696156 (LOC103696156), mRNA
GlaUn067864	15.42	4.2E-08	0.3	0.00031	PREDICTED: Phoenix dactylifera uncharacterized LOC103696156 (LOC103696156), mRNA
GlaUn068335	2.41	0.00166	0.05	1E-57	PREDICTED: Elaeis guineensis uncharacterized LOC105038671 (LOC105038671), transcript variant X1, mRNA >gi|743765268|ref|XM_010914548.1| PREDICTED: Elaeis guineensis uncharacterized LOC105038671 (LOC105038671), transcript variant X2, mRNA
GlaUn068389	3.51	0.03593	3.85	3.4E-12	PREDICTED: Phoenix dactylifera UDP-arabinopyranose mutase 1 (LOC103704494), mRNA
GlaUn068414	0.42	0.00245	0.13	6.7E-08	-
GlaUn068927	7.47	0.00446	2.53	0.00156	PREDICTED: Eucalyptus grandis transcription factor RAX2-like (LOC104436278), mRNA
GlaUn068977	0.46	0.03895	0.41	0.00067	Freycinetia banksii voucher S.W. Graham 02-03-14,UBC NADH-plastoquinone oxidoreductase subunit 7 (ndhH) gene, complete cds; plastid
GlaUn069063	10.15	5.1E-06	2.88	2.2E-05	Medicago truncatula clone mth2-61k18, complete sequence
GlaUn069064	10.13	1.7E-06	2.84	0.00028	Medicago truncatula clone mth2-61k18, complete sequence
GlaUn069065	10.26	2.9E-05	2.91	1.6E-05	Medicago truncatula clone mth2-61k18, complete sequence
GlaUn069129	8.35	6.4E-06	0.47	0.0225	-
GlaUn069174	0.35	0.00189	0.25	0.00031	-
GlaUn069369	3.79	3.8E-11	2.39	6.2E-05	PREDICTED: Musa acuminata subsp. malaccensis glutamate decarboxylase 4-like (LOC103995822), mRNA
GlaUn069654	2.03	0.0026	3.78	1E-08	Oryza sativa Japonica Group DNA, chromosome 2, cultivar: Nipponbare, complete sequence

GlaUn069670	6.25	0.00378	0.42	0.02484	-
GlaUn069795	0.42	0.04726	0.42	0.00964	-
GlaUn069978	2.36	0.00125	0.14	4.3E-14	-
GlaUn069984	2.46	0.01093	0.12	3.8E-08	-
GlaUn069999	9.76	0.02426	3.53	0.00457	Oryza sativa Japonica Group DNA, chromosome 2, cultivar: Nipponbare, complete sequence
GlaUn070057	5.28	8.4E-08	11.12	0.02287	PREDICTED: Nicotiana sylvestris uncharacterized LOC104217556 (LOC104217556), mRNA
GlaUn070058	4.71	5.3E-07	10.83	0.02397	PREDICTED: Nicotiana sylvestris uncharacterized LOC104217556 (LOC104217556), mRNA
GlaUn070059	4.23	1.9E-11	10.33	0.01619	PREDICTED: Nicotiana sylvestris uncharacterized LOC104217556 (LOC104217556), mRNA
GlaUn070060	4.7	6E-07	10.71	0.0244	PREDICTED: Nicotiana sylvestris uncharacterized LOC104217556 (LOC104217556), mRNA
GlaUn070061	4.08	3.6E-11	10.19	0.01866	PREDICTED: Nicotiana sylvestris uncharacterized LOC104217556 (LOC104217556), mRNA
GlaUn070201	2.78	0.02067	3.85	0.00092	Gladiolus grandiflorus SAM synthetase 1 (SAM1) mRNA, complete cds
GlaUn070484	6.35	0.00471	5.85	7.7E-05	-
GlaUn070525	0.36	4.2E-06	0.29	1.4E-06	PREDICTED: Phoenix dactylifera serine/threonine-protein kinase HT1-like (LOC103701974), transcript variant X2, mRNA
GlaUn070781	4.04	0.00352	13.69	2.9E-20	Aloe arborescens type III polyketide synthase (PKS5) mRNA, complete cds
GlaUn070783	3.36	0.04436	14.23	3.4E-15	Aloe arborescens type III polyketide synthase (PKS5) mRNA, complete cds
GlaUn070785	4.04	0.0002	10.02	7.7E-14	Aloe arborescens type III polyketide synthase (PKS5) mRNA, complete cds
GlaUn071399	2.5	0.02332	2.92	2.6E-06	PREDICTED: Elaeis guineensis RNA polymerase sigma factor sigE, chloroplastic/mitochondrial (LOC105039465), transcript variant X2, mRNA >gi|743755896|ref|XM_010915640.1| PREDICTED: Elaeis guineensis RNA polymerase sigma factor sigE, chloroplastic/mitochondrial (LOC105039465), transcript variant X3, mRNA
GlaUn071508	4.51	9.2E-12	16.12	8.5E-11	PREDICTED: Phoenix dactylifera UDP-glycosyltransferase 85A2-like (LOC103717956), mRNA
GlaUn072285	2.67	0.02057	4.43	1.4E-08	PREDICTED: Musa acuminata subsp. malaccensis G-type lectin S-receptor-like serine/threonine-protein kinase At5g35370 (LOC103975526), mRNA
GlaUn072515	4.27	0.04517	4.01	9.5E-09	PREDICTED: Phoenix dactylifera short-chain dehydrogenase reductase 3b-like (LOC103723725), mRNA
GlaUn072936	2.76	0.01689	3.04	0.00013	PREDICTED: Elaeis guineensis fructose-1,6-bisphosphatase, cytosolic (LOC105049443), mRNA
GlaUn073098	0.33	0.01645	0.16	0.00976	-
GlaUn073099	0.41	0.04687	0.14	2.6E-08	-
GlaUn073103	0.42	0.02967	0.15	4.9E-08	-
GlaUn073126	0.48	0.02943	0.25	4.1E-06	PREDICTED: Phoenix dactylifera serine/arginine-rich-splicing factor SR34-like (LOC103707242), transcript variant X5, misc_RNA
GlaUn073181	3.45	0.00057	2.53	0.00193	-
GlaUn073325	10.92	0.00133	0.4	0.04006	PREDICTED: Elaeis guineensis protein EXORDIUM-like (LOC105056796), mRNA
GlaUn073401	2.29	0.00042	15.36	6.4E-24	Morus notabilis Putative methyltransferase partial mRNA
GlaUn073498	10.76	8.3E-17	0.39	1.8E-05	-
GlaUn073499	11.82	9.5E-17	0.36	8.3E-06	-
GlaUn073536	0.4	0.00548	0.09	3.4E-13	-
GlaUn073711	15.74	2.3E-19	0.2	1.5E-12	-
GlaUn073712	17.38	5.7E-27	0.17	1E-15	-
GlaUn073876	2.29	0.03655	4.62	0.0006	PREDICTED: Brachypodium distachyon uncharacterized acetyltransferase At3g50280-like (LOC100843759), mRNA
GlaUn073878	2.35	0.03052	4.5	0.00054	PREDICTED: Brachypodium distachyon uncharacterized acetyltransferase At3g50280-like (LOC100843759), mRNA
GlaUn074018	3.77	0.03821	6.59	4.7E-22	-
GlaUn074019	9.17	0.00352	5.84	3.4E-13	-
GlaUn074023	2.87	0.01864	2.78	7.8E-07	PREDICTED: Phoenix dactylifera mitogen-activated protein kinase kinase 5 (LOC103718404), mRNA
GlaUn074024	2.51	0.04478	3.07	3.1E-08	PREDICTED: Phoenix dactylifera mitogen-activated protein kinase kinase 5 (LOC103718404), mRNA
GlaUn074027	2.25	0.03315	3.69	6E-11	PREDICTED: Phoenix dactylifera mitogen-activated protein kinase kinase 5 (LOC103718404), mRNA

GlaUn074358	2.52	0.02809	2.74	0.02228	-
GlaUn074359	2.53	0.02809	2.61	0.0332	-
GlaUn074525	0.33	0.00644	0.15	5.9E-09	-
GlaUn074712	2.34	6.1E-05	0.16	1.7E-18	-
GlaUn074829	2.66	0.00989	3.54	1E-10	Narcissus pseudonarcissus S-adenosylmethionine decarboxylase (SAMdc) mRNA, complete cds
GlaUn074832	2.6	0.01827	3.14	1.1E-08	Narcissus pseudonarcissus S-adenosylmethionine decarboxylase (SAMdc) mRNA, complete cds
GlaUn075216	2.29	0.03447	0.15	5.5E-08	-
GlaUn075362	3.43	1.3E-06	0.13	9.3E-16	-
GlaUn076110	3.44	0.00408	2.14	0.01003	PREDICTED: Musa acuminata subsp. malaccensis probable 2-aminoethanethiol dioxygenase (LOC103995095), mRNA
GlaUn076216	0.43	0.01625	0.35	0.00217	-
GlaUn076317	14.65	4.1E-05	0.34	0.00951	PREDICTED: Nicotiana sylvestris formin-like protein 4 (LOC104244000), mRNA
GlaUn076318	15.33	1.5E-05	0.34	0.00683	PREDICTED: Nicotiana sylvestris formin-like protein 4 (LOC104244000), mRNA
GlaUn076824	4.22	0.00014	3.16	6.3E-09	PREDICTED: Phoenix dactylifera 4-alpha-glucanotransferase DPE1, chloroplastic/amyloplastic (LOC103721718), mRNA
GlaUn076835	5.27	0.04242	49.07	2.1E-19	PREDICTED: Phoenix dactylifera trans-resveratrol di-O-methyltransferase-like (LOC103706155), mRNA
GlaUn076993	3.38	0.00205	6.81	5.8E-16	PREDICTED: Phoenix dactylifera pentatricopeptide repeat-containing protein At5g66520-like (LOC103717324), mRNA
GlaUn077347	2.03	0.03747	0.22	9.2E-08	-
GlaUn077349	2.51	2.8E-06	0.13	8E-25	-
GlaUn077350	2.53	2.4E-06	0.13	2.7E-25	-
GlaUn077352	2.14	0.00879	0.18	6.4E-11	-
GlaUn077353	2.5	3.2E-06	0.13	1.9E-25	-
GlaUn077354	2.17	0.00057	0.19	2.4E-15	-
GlaUn077524	3.78	0.01363	3.13	1.8E-07	PREDICTED: Phoenix dactylifera anamorsin homolog (LOC103717059), transcript variant X2, mRNA
GlaUn077525	3.37	0.02043	3.96	3.7E-11	PREDICTED: Phoenix dactylifera anamorsin homolog (LOC103717059), transcript variant X2, mRNA
GlaUn077526	3.26	0.00035	3.57	1.7E-09	-
GlaUn078100	0.45	0.02344	8.68	9.8E-24	-
GlaUn078254	0.07	0.00161	1267.27	1.1E-82	PREDICTED: Elaeis guineensis uncharacterized acetyltransferase At3g50280 (LOC105046267), mRNA
GlaUn078691	11.94	0.00611	0.2	0.00872	PREDICTED: Elaeis guineensis chitin-inducible gibberellin-responsive protein 1-like (LOC105046940), mRNA
GlaUn078692	12.08	0.00503	0.19	0.00704	PREDICTED: Elaeis guineensis chitin-inducible gibberellin-responsive protein 1-like (LOC105046940), mRNA
GlaUn078734	2.07	0.0157	2.18	0.00036	Ananas bracteatus clone 50906d microsatellite sequence
GlaUn078735	2.05	0.01937	2.22	0.00025	Ananas bracteatus clone 50906d microsatellite sequence
GlaUn078852	2.01	0.02907	4.18	4.7E-13	Gladiolus hybrid cultivar protein phosphatase 2C (PP2C1) mRNA, complete cds
GlaUn079002	0.35	0.02263	0.13	0.00033	-
GlaUn079233	2.06	0.00015	0.18	8.7E-20	-
GlaUn079235	2.45	8.4E-07	0.19	1.3E-20	-
GlaUn079337	3.42	0.00022	0.07	4.2E-15	Cucumis melo genomic chromosome, chr_9
GlaUn079971	4.38	0.0018	2.3	8.9E-05	PREDICTED: Elaeis guineensis UDP-glucose 6-dehydrogenase 5-like (LOC105058153), mRNA
GlaUn079972	4.04	0.00366	2.27	0.00014	PREDICTED: Zea mays LOC100282847 (gpm540b), transcript variant X1, mRNA
GlaUn079979	10.67	3E-15	3.95	4.2E-07	PREDICTED: Phoenix dactylifera G-type lectin S-receptor-like serine/threonine-protein kinase SD2-5 (LOC103705662), mRNA
GlaUn080047	4.31	9.8E-18	0.11	5.8E-36	PREDICTED: Malus x domestica transcription factor bHLH63 (LOC103440396), transcript variant X2, mRNA
GlaUn080048	3.4	4.6E-12	0.14	9.4E-28	PREDICTED: Malus x domestica transcription factor bHLH63 (LOC103440396), transcript variant X2, mRNA
GlaUn080049	4.03	2.6E-16	0.11	5.7E-35	PREDICTED: Malus x domestica transcription factor bHLH63 (LOC103440396), transcript variant X2, mRNA
GlaUn080050	4.03	2.6E-16	0.11	5.9E-35	PREDICTED: Malus x domestica transcription factor bHLH63 (LOC103440396), transcript variant X2, mRNA
GlaUn080051	4.45	1.7E-18	0.11	1.5E-36	PREDICTED: Malus x domestica transcription factor bHLH63 (LOC103440396), transcript variant X2, mRNA
GlaUn080052	3.6	8.1E-12	0.11	4.9E-29	PREDICTED: Prunus mume transcription factor bHLH63 (LOC103335662), mRNA

GlaUn080053	4.26	1.6E-17	0.11	2.5E-36	PREDICTED: Malus x domestica transcription factor bHLH63 (LOC103440396), transcript variant X2, mRNA
GlaUn080055	4.62	8.2E-19	0.11	4.2E-36	PREDICTED: Prunus mume transcription factor bHLH63 (LOC103335662), mRNA
GlaUn080056	3.41	4.2E-12	0.14	8.7E-28	PREDICTED: Malus x domestica transcription factor bHLH63 (LOC103440396), transcript variant X2, mRNA
GlaUn080057	3.5	3.6E-13	0.12	5.3E-33	PREDICTED: Malus x domestica transcription factor bHLH63 (LOC103440396), transcript variant X2, mRNA
GlaUn080058	4.13	7.7E-17	0.11	3.4E-35	PREDICTED: Malus x domestica transcription factor bHLH63 (LOC103440396), transcript variant X2, mRNA
GlaUn080128	2.09	0.00045	0.17	1.5E-18	-
GlaUn080129	2.03	0.00071	0.17	3.3E-19	-
GlaUn080849	12.58	0.01067	3.95	4.2E-09	PREDICTED: Brachypodium distachyon protein DETOXIFICATION 49-like (LOC100822136), mRNA
GlaUn080910	0.38	0.02707	0.49	0.04843	-
GlaUn080954	9.05	0.00063	0.39	0.02981	-
GlaUn080959	24.22	0.00251	0.19	0.0145	-
GlaUn081137	12.21	0.00097	5.61	6.7E-10	PREDICTED: Phoenix dactylifera omega-3 fatty acid desaturase, chloroplastic (LOC103709505), mRNA
GlaUn081138	11.84	0.00125	5.36	1.5E-09	PREDICTED: Phoenix dactylifera omega-3 fatty acid desaturase, chloroplastic (LOC103709505), mRNA
GlaUn081217	4	0.00668	2.63	0.00127	PREDICTED: Phoenix dactylifera cleavage stimulation factor subunit 2-like (LOC103703217), transcript variant X3, mRNA
GlaUn081270	4.5	0.00233	0.07	1.7E-08	-
GlaUn081393	3.06	0.01262	3.81	0.00592	-
GlaUn081532	3.36	0.04932	2.59	1.3E-05	PREDICTED: Nelumbo nucifera uncharacterized serine-rich protein C215.13 (LOC104598892), mRNA
GlaUn081611	6.1	0.00443	2.73	0.00164	PREDICTED: Elaeis guineensis probable receptor-like protein kinase At1g67000 (LOC105036386), partial mRNA
GlaUn081698	2.21	2.5E-05	0.11	1.5E-30	PREDICTED: Elaeis guineensis probable protein phosphatase 2C 75 (LOC105043050), transcript variant X2, mRNA
GlaUn081699	2.15	4.8E-05	0.11	1.1E-30	PREDICTED: Elaeis guineensis probable protein phosphatase 2C 75 (LOC105043050), transcript variant X2, mRNA
GlaUn081700	2.13	6.1E-05	0.11	5.4E-30	PREDICTED: Elaeis guineensis probable protein phosphatase 2C 75 (LOC105043050), transcript variant X2, mRNA
GlaUn081701	2.16	3.7E-05	0.11	1.8E-30	PREDICTED: Elaeis guineensis probable protein phosphatase 2C 75 (LOC105043050), transcript variant X2, mRNA
GlaUn081702	2.16	0.00131	0.13	2.4E-17	PREDICTED: Elaeis guineensis probable protein phosphatase 2C 75 (LOC105053585), transcript variant X6, mRNA
GlaUn081703	2.43	1.7E-05	0.19	2.2E-16	PREDICTED: Elaeis guineensis probable protein phosphatase 2C 75 (LOC105053585), transcript variant X4, mRNA
GlaUn081845	0.3	0.00076	0.08	7E-07	-
GlaUn082334	30.79	0.00011	0.19	0.00514	-
GlaUn082335	30.71	2.8E-05	0.19	0.0024	-
GlaUn082426	7.03	0.00427	4.99	6.2E-05	-
GlaUn082717	2.58	0.00408	0.08	8.2E-16	PREDICTED: Musa acuminata subsp. malaccensis uncharacterized LOC103974645 (LOC103974645), mRNA
GlaUn082747	0.47	0.03268	0.45	0.00083	PREDICTED: Elaeis guineensis probable tetraacyldisaccharide 4'-kinase, mitochondrial (LOC105046825), transcript variant X3, mRNA
GlaUn082748	0.44	0.023	0.44	0.00883	-
GlaUn082899	2.34	0.00038	0.09	4.7E-21	-
GlaUn083070	0.23	0.00121	13.99	1.1E-24	-
GlaUn083600	6.82	0.00727	13.48	4.7E-19	PREDICTED: Eucalyptus grandis uncharacterized LOC104428535 (LOC104428535), mRNA
GlaUn083633	0.09	0.02227	69.14	1.2E-19	Sandersonia aurantiaca papain-like cysteine protease (PRT5) mRNA, complete cds
GlaUn083802	39.81	0.01584	0.12	0.03067	-
GlaUn084355	4.07	0.00405	11.29	0.00165	PREDICTED: Elaeis guineensis mitochondrial chaperone BCS1-like (LOC105056311), mRNA
GlaUn084356	3.91	0.00621	11.77	0.00177	PREDICTED: Elaeis guineensis mitochondrial chaperone BCS1-like (LOC105056311), mRNA
GlaUn084412	4.07	0.00043	4.21	2.5E-08	PREDICTED: Phoenix dactylifera 1-aminocyclopropane-1-carboxylate oxidase homolog 3-like (LOC103717337), transcript variant X4, mRNA
GlaUn084483	0.34	0.00027	0.15	1.5E-08	-
GlaUn084701	0.31	0.00389	0.21	0.00079	-
GlaUn084702	0.3	0.007	0.18	0.00109	-

GlaUn084750	0.39	0.02598	0.47	0.03646	PREDICTED: Solanum lycopersicum E3 ubiquitin ligase BIG BROTHER-like (LOC101266907), transcript variant X4, mRNA
GlaUn084967	12.59	5.2E-05	0.27	0.00227	-
GlaUn085006	6.39	0.00706	8.01	0.02245	PREDICTED: Elaeis guineensis putative receptor protein kinase ZmPK1 (LOC105050315), mRNA
GlaUn085008	5.61	1.2E-06	4.61	0.0375	PREDICTED: Elaeis guineensis putative receptor protein kinase ZmPK1 (LOC105044537), mRNA
GlaUn085168	0.47	0.03825	0.41	5.2E-05	-
GlaUn085203	18.31	0.00068	3.77	0.02216	-
GlaUn085204	11.26	0.00179	4.42	0.00053	-
GlaUn085265	3.31	0.00258	3.04	3.6E-08	PREDICTED: Nelumbo nucifera U-box domain-containing protein 17 (LOC104586555), mRNA
GlaUn085459	3.41	0.00056	6.26	1.6E-13	PREDICTED: Brachypodium distachyon putative histone-lysine N-methyltransferase PRDM6 (LOC100842321), mRNA
GlaUn085756	0.46	0.00704	0.12	4.8E-11	-
GlaUn086047	13.03	0.01874	4.77	0.00662	PREDICTED: Elaeis guineensis receptor-like protein kinase 5 (LOC105041917), mRNA
GlaUn086163	0.37	0.00073	0.27	1.6E-07	-
GlaUn086164	0.3	0.02242	0.33	0.04592	-
GlaUn086165	0.37	0.00055	0.37	2.4E-05	-
GlaUn086166	0.4	0.00114	0.29	4.4E-08	-
GlaUn086221	2.87	0.01863	2.67	1.6E-05	PREDICTED: Phoenix dactylifera arginine decarboxylase-like (LOC103705463), transcript variant X2, mRNA
GlaUn086226	11.99	0.00045	2.75	0.01848	PREDICTED: Musa acuminata subsp. malaccensis receptor-like protein kinase HSL1 (LOC103969398), mRNA
GlaUn086892	2.58	8.7E-08	0.14	4.9E-22	-
GlaUn086893	2.46	2.7E-06	0.13	6.7E-17	-
GlaUn086936	2.56	0.00029	0.06	2.6E-23	-
GlaUn086938	2.13	0.03369	0.16	4.4E-09	Daucus carota Top1beta gene, promoter region
GlaUn086941	2.2	0.00061	0.16	8.7E-17	Eriobotrya japonica clone ssrEJ092 microsatellite sequence
GlaUn087126	3.49	0.00949	11.4	3.2E-15	PREDICTED: Phoenix dactylifera probable alpha,alpha-trehalose-phosphate synthase [UDP-forming] 9 (LOC103699387), transcript variant X2, misc_RNA
GlaUn087127	3.14	0.01937	13.11	1.5E-16	PREDICTED: Phoenix dactylifera probable alpha,alpha-trehalose-phosphate synthase [UDP-forming] 9 (LOC103699387), transcript variant X2, misc_RNA
GlaUn087128	4.95	0.00804	10.61	5.9E-27	-
GlaUn087130	3.71	0.04467	12.68	5.4E-15	-
GlaUn087243	0.4	0.00041	0.48	0.00423	Arabidopsis thaliana chromosome 3, complete sequence
GlaUn087407	53.57	0.03424	5.48	2.4E-07	PREDICTED: Elaeis guineensis L-ascorbate oxidase homolog (LOC105042624), mRNA
GlaUn087409	3.14	0.00503	2.19	0.01741	PREDICTED: Elaeis guineensis glutathione S-transferase U17-like (LOC105043366), transcript variant X2, mRNA
GlaUn087586	2.52	0.0296	19.08	1.2E-20	PREDICTED: Phoenix dactylifera anthocyanidin 3-O-glucosyltransferase 4-like (LOC103710378), mRNA
GlaUn087587	2.4	0.01317	20.26	1E-16	PREDICTED: Phoenix dactylifera anthocyanidin 3-O-glucosyltransferase 4-like (LOC103710378), mRNA
GlaUn087657	0.22	0.02413	11.61	1.5E-09	-
GlaUn088256	61.05	0.00123	3.64	3.2E-06	PREDICTED: Musa acuminata subsp. malaccensis uncharacterized LOC103992886 (LOC103992886), mRNA
GlaUn088259	26.03	0.03528	3.95	0.00292	PREDICTED: Musa acuminata subsp. malaccensis uncharacterized LOC103992886 (LOC103992886), mRNA
GlaUn088301	3.11	0.00214	10.78	2.3E-19	PREDICTED: Musa acuminata subsp. malaccensis cytochrome P450 71A1-like (LOC103985373), mRNA
GlaUn088377	43.21	0.00121	0.22	0.03595	PREDICTED: Musa acuminata subsp. malaccensis probable lipoxygenase 6 (LOC103968512), mRNA
GlaUn088384	0.49	0.01211	0.35	6.4E-09	PREDICTED: Nelumbo nucifera uncharacterized LOC104592353 (LOC104592353), mRNA
GlaUn088540	2.67	0.03373	2.27	0.01642	-
GlaUn088610	0.16	4.3E-06	34.03	3.4E-19	Vigna angularis var. angularis DNA, chromosome 1, almost complete sequence, cultivar: Shumari
GlaUn088906	0.43	0.04401	0.4	0.00114	-
GlaUn088914	7.85	2.6E-16	8.09	1.1E-24	PREDICTED: Elaeis guineensis glycerol-3-phosphate 2-O-acyltransferase 6 (LOC105046257), mRNA
GlaUn088916	6.69	2.7E-12	8.33	9.5E-25	PREDICTED: Prunus mume glycerol-3-phosphate 2-O-acyltransferase 6 (LOC103338081), mRNA
GlaUn088917	3.99	0.00022	8.54	8.2E-21	PREDICTED: Elaeis guineensis glycerol-3-phosphate 2-O-acyltransferase 6 (LOC105046257), mRNA

GlaUn089118	3.87	0.00084	3.56	4.5E-07	PREDICTED: Musa acuminata subsp. malaccensis anthocyanin 3'-O-beta-glucosyltransferase-like (LOC103989223), mRNA
GlaUn089215	6.87	3.6E-13	0.43	1.7E-05	PREDICTED: Phoenix dactylifera proline-rich receptor-like protein kinase PERK3 (LOC103717896), mRNA
GlaUn089269	5.92	0.0005	3.96	0.00505	-
GlaUn089717	30.72	2.2E-10	0.25	0.00016	-
GlaUn089718	21.19	4.5E-10	0.28	0.00013	-
GlaUn089728	3.05	0.00693	2.15	0.00047	-
GlaUn089733	17.53	4.3E-06	0.29	0.00121	-
GlaUn089758	2	0.00036	0.18	5.4E-21	-
GlaUn089759	2.19	1.5E-05	0.2	3.8E-16	-
GlaUn089760	2.06	5.7E-05	0.2	1.7E-15	-
GlaUn089762	2.21	1.7E-05	0.18	2.6E-22	-
GlaUn089763	2.09	0.00011	0.18	7.7E-21	-
GlaUn090024	2.11	0.0008	0.15	9.7E-19	-
GlaUn090026	2.02	0.00017	0.15	1.7E-25	-
GlaUn090038	2.5	0.04415	0.1	1.9E-08	-
GlaUn090039	2.79	0.0214	0.03	7.9E-11	-
GlaUn090040	2.71	0.00083	0.09	1E-14	-
GlaUn090234	0.4	0.04928	0.47	0.03372	-
GlaUn090994	0.42	0.04367	0.11	2.5E-08	-
GlaUn091310	2.28	0.03636	2.73	0.00068	PREDICTED: Elaeis guineensis pentatricopeptide repeat-containing protein At2g02980 (LOC105041736), mRNA
GlaUn091423	0.26	3E-05	29.41	9.4E-52	PREDICTED: Phoenix dactylifera probable LRR receptor-like serine/threonine-protein kinase At4g37250 (LOC103716219), mRNA
GlaUn091424	0.29	0.0001	12.9	9E-33	PREDICTED: Elaeis guineensis probable LRR receptor-like serine/threonine-protein kinase At4g37250 (LOC105050162), mRNA
GlaUn091425	0.24	2.7E-05	43.49	1.7E-59	PREDICTED: Phoenix dactylifera probable LRR receptor-like serine/threonine-protein kinase At4g37250 (LOC103716219), mRNA
GlaUn091426	0.34	0.00389	9.7	4.7E-16	PREDICTED: Elaeis guineensis probable LRR receptor-like serine/threonine-protein kinase At4g37250 (LOC105050162), mRNA
GlaUn091427	0.25	1.7E-06	27.84	3.4E-51	PREDICTED: Phoenix dactylifera probable LRR receptor-like serine/threonine-protein kinase At4g37250 (LOC103716219), mRNA
GlaUn091428	0.27	8.3E-06	16.44	3.5E-38	PREDICTED: Phoenix dactylifera probable LRR receptor-like serine/threonine-protein kinase At4g37250 (LOC103716219), mRNA
GlaUn091574	4.61	0.01366	25.75	0.00663	PREDICTED: Musa acuminata subsp. malaccensis phenylpropanoylacetyl-CoA synthase (LOC103975557), mRNA
GlaUn091575	2	0.02638	35.81	8.6E-05	Aquilaria sinensis chalcone synthase (CHS2) mRNA, complete cds
GlaUn091962	0.14	0.02076	30.55	1.2E-08	PREDICTED: Phoenix dactylifera leucine-rich repeat receptor-like serine/threonine-protein kinase BAM1 (LOC103704810),
GlaUn092001	0.46	0.02585	0.46	0.00013	PREDICTED: Elaeis guineensis uncharacterized LOC105039883 (LOC105039883), transcript variant X2, mRNA
GlaUn092303	6.32	0.01516	4.13	8.1E-08	-
GlaUn092304	6.25	0.01663	3.25	9.9E-05	Cocos nucifera serine-threonine kinase STK6 mRNA, complete cds
GlaUn092305	6.24	0.0001	3.59	3.4E-06	-
GlaUn092355	26.46	0.00186	0.2	0.01393	-
GlaUn092356	40.32	0.00366	0.19	0.03331	-
GlaUn092357	25.45	0.00175	0.16	0.00874	-
GlaUn092478	10.27	0.00205	2.38	0.04135	Oryza sativa Japonica Group DNA, chromosome 5, cultivar: Nipponbare, complete sequence
GlaUn092515	5.48	0.02424	4.58	3.1E-12	PREDICTED: Pyrus x bretschneideri receptor-like protein kinase HAIKU2 (LOC103947969), mRNA
GlaUn092516	4.88	0.02726	4.6	4.4E-10	PREDICTED: Elaeis guineensis receptor-like protein kinase HAIKU2 (LOC105061016), mRNA
GlaUn092644	0.31	3.8E-05	0.14	2.5E-07	-
GlaUn093084	0.12	0.01289	72.4	0.0004	PREDICTED: Musa acuminata subsp. malaccensis auxin transporter-like protein 1 (LOC103990196), mRNA
GlaUn093087	63.43	6.1E-45	0.08	4.3E-29	-
GlaUn093252	2.66	1E-06	2.33	9.4E-05	PREDICTED: Eucalyptus grandis chaperone protein ClpB3, chloroplastic (LOC104435988), mRNA

GlaUn093253	3.13	3E-07	2.05	0.0027	PREDICTED: Sesamum indicum uncharacterized LOC105155881 (LOC105155881), mRNA
GlaUn093295	2.12	0.04722	2.43	0.00267	Vitis vinifera contig VV78X195327.12, whole genome shotgun sequence
GlaUn093540	4.92	0.02812	3.68	2.2E-07	Gladiolus hybrid cultivar cultivar Rose Supreme ABA 8'-hydroxylase 1 (CYP707A1) mRNA, partial cds
GlaUn093620	2.72	0.00597	0.13	1.4E-09	-
GlaUn097131	4.42	0.02206	9.77	2.7E-06	-
GlaUn098857	0.25	0.00046	0.02	0.00282	-
GlaUn100459	2.07	0.02427	0.2	4.5E-09	-
GlaUn101375	2.2	0.01586	0.11	4E-12	PREDICTED: Phoenix dactylifera L-ascorbate oxidase homolog (LOC103702183), mRNA
GlaUn101712	4.93	0.03916	3.12	0.00496	-
GlaUn104526	5.5	0.04463	3.4	0.00317	-
GlaUn105669	7.86	0.00702	0.38	0.03635	-
GlaUn106028	2.03	0.00033	0.23	5E-12	-
GlaUn106259	0.14	6.6E-11	0.4	0.01122	-
GlaUn106640	7.23	0.03434	2.39	0.02498	-
GlaUn111155	0.47	0.04572	0.15	1.4E-14	-
GlaUn111575	4.16	0.01874	3.28	0.04869	-
GlaUn111911	0.4	0.00012	0.03	3.9E-17	-
GlaUn113783	0.27	0.01332	0.12	0.00611	-
GlaUn115355	0.36	0.00443	0.12	5.8E-08	-
GlaUn116606	6.92	8.6E-06	2.6	0.02756	-
GlaUn117191	4.87	1E-04	0.05	1.8E-10	-
GlaUn117239	6.1	0.0287	14.5	2.8E-14	-
GlaUn117439	68.71	0.03564	6.06	1.8E-05	-
GlaUn117844	3.17	0.01858	0	9.2E-15	-
GlaUn117845	3.21	0.01991	0	6.9E-15	-
