# Supplementary material for: GhNAC83 inhibits corm dormancy release by regulating ABA signaling and cytokinin biosynthesis in Gladiolus hybridus
Source: J Exp Bot. 2018 Dec 5;70(4):1221–37. doi: 10.1093/jxb/ery428 (PMC6382327; doi:10.1093/jxb/ery428)
Supplement: Supplementary Table S3 [file ery428_suppl_supplementary_table_s3.pdf]

Table S3. List of differentially expressed cytokinins- and ABA- related genes

Gene ID	KEGG ID	putative function	WD VS DD		ED VS WD	
			Fold	Log_2FC	Fold	Log_2FC
Zeatin metabolism						
GlaUn027415	K13495	CISZOG	21.67	0.809970186	20.86	4.22E-07
GlaUn049027	K13495	CISZOG	0.88	1	3.83	0.003108116
GlaUn011664	K13495	CISZOG	0.72	0.891772388	3.27	3.66E-05
GlaUn091771	K13495	CISZOG	1.38	0.731124316	5.62	5.67E-09
GlaUn033550	K13495	CISZOG	2.1	0.872435129	25.96	2.94E-37
GlaUn074244	K13495	CISZOG	2.67	0.684488876	27.39	3.59E-58
GlaUn049030	K13495	CISZOG	0.94	1	2.2	0.046310478
GlaUn049028	K13495	CISZOG	0.98	1	4.32	9.28E-06
GlaUn027416	K13495	CISZOG	2.1	1	35.67	9.63E-07
GlaUn091770	K13495	CISZOG	6	0.553867568	16.62	1.99E-12
GlaUn091769	K13495	CISZOG	1.24	0.416584357	5.86	3.10E-19
GlaUn065024	K13495	CISZOG	0.54	0.640582769	64.25	1.25E-76
GlaUn108585	K13495	CISZOG	9.67	0.998003285	26.72	0.001835285
GlaUn057969	K13493	UGT76C	0.26	0.257540159	4.58	0.025443248
GlaUn003748	K13493	UGT76C	0.91	1	5.65	3.84E-18
GlaUn063553	K13493	UGT76C	0.43	0.049841553	2.37	0.009952164
GlaUn071508	K13493	UGT76C	4.51	9.20E-12	16.12	8.47E-11
GlaUn083354	K13493	UGT76C	0.83	1	5.92	2.22E-16
GlaUn071511	K13493	UGT76C	4.58	0.227672272	5.32	6.04E-12
GlaUn071509	K13493	UGT76C	1.36	0.938977157	72.92	2.96E-17
GlaUn031858	K13493	UGT76C	0.52	1	60.87	5.89E-06
GlaUn079045	K13493	UGT76C	1.72	0.456880791	6.49	1.11E-08
GlaUn048661	K13493	UGT76C	1.52	0.078265988	0.45	1.14E-05
GlaUn020036	K13493	UGT76C	1.77	0.978487881	11.03	3.27E-05
GlaUn084925	K13493	UGT76C	0.42	0.150345729	2.64	0.015203105
GlaUn063555	K13493	UGT76C	0.79	1	4.28	3.78E-05
GlaUn071512	K13493	UGT76C	5.47	0.239485393	5.62	6.15E-08
GlaUn071510	K13493	UGT76C	1.36	0.215378969	7.82	7.75E-17
GlaUn054456	K10760	AtIPT	0.29	1	181.5	9.52E-19
GlaUn006126	K10760	AtIPT	0.07	0.959717144	87	0.140267289
GlaUn027324	K00791	TRIT1	2.32	0.802169037	2.35	0.275355808
GlaUn052173	K10717	CYP735A	1.04	0.90078541	1.14	1
GlaUn052168	K10717	CYP735A	1.05	0.891772388	1.12	0.972862766
GlaUn048874	K10717	CYP735A	0.75	1	1	NA
GlaUn067098	K10717	CYP735A	4.47	0.598939194	9.33	0.021857261
GlaUn052172	K10717	CYP735A	1.04	0.912205011	0.95	0.485893671
GlaUn067100	K10717	CYP735A	5	1	14.6	0.211383408
GlaUn069615	K10717	CYP735A	0.65	0.733963262	2.25	0.000211978
GlaUn116903	K00279	CKX	0.21	1	28	0.413403935
GlaUn068688	K00279	CKX	2.51	0.000176893	0.97	0.587995997
GlaUn003773	K00279	CKX	1.92	0.004098349	0.61	0.006077591
GlaUn083844	K00279	CKX	1.88	0.005372696	0.57	0.002085457
GlaUn056220	K00279	CKX	1.75	0.699687815	0.46	0.212034331
GlaUn011167	K00279	CKX	0.12	0.640817398	23	0.316277335
GlaUn073350	K00279	CKX	0.3	0.587123745	36.9	2.26E-05
GlaUn073352	K00279	CKX	3.33	1	16.3	0.094611398
GlaUn034211	K00279	CKX	1	NA	25	0.148592335
GlaUn073351	K00279	CKX	3.33	1	18.3	0.080985632
ABA metabolism						
GlaUn054029	K09838	ABA1	0.76	0.945475832	11.26	4.94E-27
GlaUn064550	K09838	ABA1	0.92	1	18.71	1.10E-10
GlaUn029851	K09838	ABA1	0.53	1	153.78	0.00267479
GlaUn000657	K09838	ABA1	0.83	1	29.74	3.33E-08
GlaUn064550	K09838	ABA1	0.92	1	18.71	1.10E-10
GlaUn029851	K09838	ABA1	0.53	1	153.78	0.00267479
GlaUn054029	K09838	ABA1	0.76	0.945475832	11.26	4.94E-27
GlaUn000657	K09838	ABA1	0.83	1	29.74	3.33E-08
GlaUn074529	K09839	NPQ1	0.79	0.963809723	0.34	5.66E-06
GlaUn057783	K09840	NCED	0.86	1	0.28	7.44E-10
GlaUn057782	K09840	NCED	0.94	1	0.26	5.83E-11
GlaUn057784	K09840	NCED	0.79	0.988664655	0.21	1.95E-06
GlaUn093539	K09843	CYP707A	4.85	0.058192747	3.53	5.71E-06
GlaUn093540	K09843	CYP707A	4.92	0.028124268	3.68	2.17E-07
GlaUn093541	K09843	CYP707A	5.07	0.059292817	3.29	2.64E-05
GlaUn061060	K09843	CYP707A	5.41	0.013403302	3.6	3.86E-08
GlaUn037869	K09843	CYP707A	16.63	0.081004548	5.28	2.28E-07
GlaUn061059	K09843	CYP707A	14.38	0.148000509	4.79	3.95E-07
GlaUn061061	K09843	CYP707A	14.38	0.146081177	4.91	6.88E-08
GlaUn093542	K09843	CYP707A	4.75	0.083734984	3.62	1.33E-06

GlaUn018811	K14595	AOG	1.65	0.786475581	5.03	0.000153964
GlaUn087588	K14595	AOG	1.67	0.629341063	24.79	1.08E-38
GlaUn018812	K14595	AOG	1.11	1	6.11	1.30E-05
GlaUn050755	K14595	AOG	1.15	1	3.75	0.008583359
GlaUn087589	K14595	AOG	2.72	0.126094085	22.07	8.02E-20
GlaUn021852	K14595	AOG	2.42	0.938382443	10.75	1.46E-06
GlaUn002060	K14595	AOG	1.14	0.901459652	3.74	1.65E-08
GlaUn001239	K14595	AOG	1.95	0.544832236	8.78	9.74E-08
GlaUn002059	K14595	AOG	1.22	0.849941492	6.33	1.86E-16
GlaUn089119	K14595	AOG	1.68	0.044148362	2.15	0.00087865
GlaUn089118	K14595	AOG	3.87	0.000837127	3.56	4.51E-07
GlaUn072422	K14595	AOG	1.33	0.839340688	40.7	4.16E-58
GlaUn072424	K14595	AOG	1.1	0.981508673	10.93	5.32E-18
GlaUn081393	K14595	AOG	3.06	0.012618162	3.81	0.005923883
GlaUn072421	K14595	AOG	1.43	0.790567311	38.38	3.46E-56
Zeatin signaling						
GlaUn048853	K14489	CRE1	0.69	0.8134011	0.18	1.68E-06
GlaUn067216	K14489	CRE1	0.81	0.936101126	0.19	2.01E-21
GlaUn052261	K14489	CRE1	2	1	149	1.61E-06
GlaUn096053	K14489	CRE1	1.77	0.563476694	0.22	0.005744931
GlaUn067215	K14489	CRE1	0.81	0.933582723	0.19	2.82E-21
GlaUn071118	K14489	CRE1	1.29	0.768971803	0.34	0.003471881
GlaUn002631	K14489	CRE1	0.81	0.934078844	0.2	3.69E-21
GlaUn048852	K14489	CRE1	0.71	0.874182829	0.16	5.37E-06
GlaUn076331	K14494	ARR-B	0.99	1	0.43	0.002655296
GlaUn076330	K14494	ARR-B	1.01	1	0.46	0.009067909
GlaUn071707	K14494	ARR-B	1.53	0.708152256	2.21	0.006919047
GlaUn076742	K14494	ARR-B	1	0.958958794	3.96	6.23E-12
GlaUn071709	K14494	ARR-B	1.01	0.955189701	3.6	2.30E-10
GlaUn031176	K14494	ARR-B	1.79	0.594139216	2.11	0.030920306
GlaUn050605	K14494	ARR-B	1.03	0.912228299	3.47	6.85E-09
GlaUn076736	K14494	ARR-B	1.01	0.950534623	3.51	1.69E-09
GlaUn092328	K14494	ARR-B	0.74	0.717194209	0.47	2.34E-06
GlaUn009047	K14494	ARR-B	2.1	1	13.52	0.043662317
GlaUn071710	K14494	ARR-B	1.04	0.897920974	3.44	1.86E-09
GlaUn076740	K14494	ARR-B	1.02	0.93323267	3.51	1.69E-09
GlaUn025694	K14494	ARR-B	2.11	0.147460299	0.14	2.63E-06
GlaUn076738	K14494	ARR-B	1	0.969103804	3.93	3.53E-11
GlaUn071708	K14494	ARR-B	1.08	0.791436562	3.58	2.40E-10
GlaUn063865	K14492	ARR-A	0.63	0.756494277	3.11	0.012767719
GlaUn063867	K14492	ARR-A	0.68	0.936101126	3.17	0.028899304
GlaUn032468	K14492	ARR-A	0.18	0.245284503	9.78	0.020051737
GlaUn002436	K14492	ARR-A	0.7	0.894493541	2.82	0.007625923
GlaUn032466	K14492	ARR-A	0.18	0.165523149	9.31	0.001669473
GlaUn063866	K14492	ARR-A	0.67	0.830749691	2.99	0.005669633
GlaUn032469	K14492	ARR-A	0.16	0.194378676	9.88	0.027788561
GlaUn010457	K14492	ARR-A	1.23	0.90804015	3.17	0.003705919
GlaUn051122	K14492	ARR-A	2.47	0.01464517	0.38	9.44E-06
GlaUn032467	K14492	ARR-A	0.17	0.129552098	9.12	0.00201856
GlaUn051121	K14492	ARR-A	1.89	0.236966948	0.34	5.09E-06
ABA signaling						
GlaUn025488	K14496	PYL	0.57	0.125515422	0.44	0.000512511
GlaUn030679	K14497	PP2C	5.23	2.59E-16	2.74	0.00000348
GlaUn052869	K14497	PP2C	4.05	3.88E-11	2.28	0.000453825
GlaUn072818	K14497	PP2C	2.36	7.0588E-07	0.32	1.12729E-12
GlaUn081699	K14497	PP2C	2.15	4.78E-05	0.11	1.07E-30
GlaUn081704	K14497	PP2C	3.78	0.00219374	0.44	0.003994378
GlaUn072819	K14497	PP2C	2.99	1.83E-09	0.31	3.92E-13
GlaUn072820	K14497	PP2C	3.11	4.40E-09	0.31	3.32E-13
GlaUn081707	K14497	PP2C	4.17	0.015785708	0.47	0.039210772
GlaUn081701	K14497	PP2C	2.16	3.74E-05	0.11	1.75E-30
GlaUn081702	K14497	PP2C	2.16	0.001313211	0.13	2.43E-17
GlaUn026473	K14497	PP2C	1.8	0.001747729	0.38	4.20E-09
GlaUn081703	K14497	PP2C	2.43	1.69E-05	0.19	2.16E-16
GlaUn078852	K14497	PP2C	2.01	0.029069561	4.18	4.70E-13
GlaUn072816	K14497	PP2C	2.95	1.01E-10	0.3	1.12E-13
GlaUn072817	K14497	PP2C	3.14	9.03E-09	0.31	2.27E-13
GlaUn081705	K14497	PP2C	4.14	0.018886338	0.38	0.014655617
GlaUn081706	K14497	PP2C	4.04	0.017524497	0.38	0.012921162
GlaUn081700	K14497	PP2C	2.13	6.08E-05	0.11	5.43E-30
GlaUn081698	K14497	PP2C	2.21	2.54E-05	0.11	1.47E-30
GlaUn026955	K14498	SnRK2	0.93	1	3.71	2.53E-09
GlaUn015304	K14498	SnRK2	0.54	0.87711972	4.39	0.013446056
GlaUn064508	K14498	SnRK2	0.9	1	2.01	0.005181329

GlaUn069238	K14498	SnRK2	1.02	0.942194783	2.34	0.00033931
GlaUn031116	K14498	SnRK2	0.81	0.934058657	7.83	3.34E-26
GlaUn030699	K14498	SnRK2	0.92	1	2.2	0.001777291
GlaUn069237	K14498	SnRK2	1.02	0.916521467	2.6	7.34E-06
GlaUn015862	K14498	SnRK2	1.32	0.351505314	0.22	1.40E-12
GlaUn028627	K14498	SnRK2	0.82	1	5.53	0.000470854
GlaUn035378	K14432	ABF	0.46	0.981508673	16.47	2.66E-05
GlaUn087511	K14432	ABF	0.86	1	3.06	5.65E-07
GlaUn077778	K14432	ABF	0.89	1	3.79	1.04E-09
GlaUn073732	K14432	ABF	0.8	0.953310969	2.8	3.39E-05
GlaUn067955	K14432	ABF	2.81	0.157776702	5.71	2.30E-09
GlaUn087510	K14432	ABF	0.92	1	2.98	6.38E-07
